# Efficient data labeling strategies for automated muscle segmentation in lower leg MRIs of Charcot-Marie-Tooth disease patients

**DOI:** 10.1371/journal.pone.0310203

**Published:** 2024-09-06

**Authors:** Seung-Ah Lee, Hyun Su Kim, Ehwa Yang, Young Cheol Yoon, Ji Hyun Lee, Byung-Ok Choi, Jae-Hun Kim

**Affiliations:** 1 Research Institute for Future Medicine, Samsung Medical Center, Sungkyunkwan University School of Medicine, Seoul, South Korea; 2 Department of Radiology, Samsung Medical Center, Sungkyunkwan University School of Medicine, Seoul, South Korea; 3 Sungkyunkwan University School of Medicine, Seoul, South Korea; 4 Department of Neurology, Samsung Medical Center, Sungkyunkwan University School of Medicine, Seoul, South Korea; Fondazione Policlinico Universitario Gemelli IRCCS, ITALY

## Abstract

We aimed to develop efficient data labeling strategies for ground truth segmentation in lower-leg magnetic resonance imaging (MRI) of patients with Charcot-Marie-Tooth disease (CMT) and to develop an automated muscle segmentation model using different labeling approaches. The impact of using unlabeled data on model performance was further examined. Using axial T1-weighted MRIs of 120 patients with CMT (60 each with mild and severe intramuscular fat infiltration), we compared the performance of segmentation models obtained using several different labeling strategies. The effect of leveraging unlabeled data on segmentation performance was evaluated by comparing the performances of few-supervised, semi-supervised (mean teacher model), and fully-supervised learning models. We employed a 2D U-Net architecture and assessed its performance by comparing the average Dice coefficients (ADC) using paired t-tests with Bonferroni correction. Among few-supervised models utilizing 10% labeled data, labeling three slices (the uppermost, central, and lowermost slices) per subject exhibited a significantly higher ADC (90.84±3.46%) compared with other strategies using a single image slice per subject (uppermost, 87.79±4.41%; central, 89.42±4.07%; lowermost, 89.29±4.71%, *p* < 0.0001) or all slices per subject (85.97±9.82%, *p* < 0.0001). Moreover, semi-supervised learning significantly enhanced the segmentation performance. The semi-supervised model using the three-slices strategy showed the highest segmentation performance (91.03±3.67%) among 10% labeled set models. Fully-supervised model showed an ADC of 91.39±3.76. A three-slice-based labeling strategy for ground truth segmentation is the most efficient method for developing automated muscle segmentation models of CMT lower leg MRI. Additionally, semi-supervised learning with unlabeled data significantly enhances segmentation performance.

## Introduction

Charcot–Marie–Tooth disease (CMT) is the most common hereditary neuromuscular disorder globally and is associated with numerous gene mutations linked to axonal degeneration [1]. CMT is characterized by distal-predominant limb muscle wasting and sensory loss [1], making careful assessment and monitoring of affected muscles crucial for proper patient management. Magnetic resonance imaging (MRI) plays a significant role in the evaluation of patients with CMT, allowing an objective assessment of limb muscle volume and quality [2–5]. Muscle denervation leads to the degeneration of fibers and their replacement by intramuscular fat cells, identifiable as volume loss and increased intramuscular fat extent on MRI [6, 7]. MRI biomarkers, including muscle volume, fat fraction, and pattern of muscle denervation with specific regard to muscle compartments in the lower legs, are clinically important for prognosis estimation, clinical assessment, and identification of certain genetic types in patients [8–12].

Extracting MRI biomarkers necessitates muscle segmentation with regions of interest (ROI) lying within the muscle compartments, excluding other structures such as the subcutaneous fat tissue or bones. However, manual segmentation is both time-consuming and labor-intensive, as well as subject to intra- and interobserver variances [13]. Several deep-learning-based models have recently emerged as promising tools for automated muscle segmentation, which could provide effective solutions for MRI analyses that currently rely on manual segmentation [7, 14–18]. However, developing such automated segmentation requires access to training data comprising manually segmented images [7, 14]. Muscle segmentation requires meticulous handling to define the boundary between the muscles and subcutaneous fat tissue, particularly in CMT patients with intramuscular fat infiltration and volume loss on MRI [3]. Thus, manual ground truth segmentation of MRIs scans of patients with CMT can be substantially labor-intensive, and data on the most efficient method for this process are limited.

Although there is currently no cure for CMT, numerous clinical and preclinical studies involving therapeutic agents have shown promising results [19–21]. Consequently, it is imperative to develop and establish an objective and reproducible method for evaluating patients with CMT, including assessing their response to treatment. To our knowledge, most previous studies have focused on developing automated segmentation models for thigh muscles on MRI [7, 14, 22, 23]. This focus might be attributed to the less complex anatomy and larger muscle volume of the thighs, making segmentation less challenging compared to that of the lower legs. However, lower leg muscles are more severely affected in CMT due to the characteristic distal-predominant limb muscle wasting. Therefore, it would be more beneficial to develop a model that can accurately segment muscles in the lower legs. In this context, we assume that the development of a robust and reliable automated muscle segmentation model for the evaluation of lower leg muscles on MRI represents a challenging yet highly desirable long-term objective.

Herein, we investigated the most efficient data labeling strategy for ground truth segmentation of the lower leg muscles in the MRIs of patients with CMT to develop an automated muscle segmentation model. We compared the performance of the segmentation models obtained using several different labeling strategies for the training data. Additionally, we investigated the effect of utilizing unlabeled data to enhance muscle segmentation performance by comparing the fully-supervised, few-supervised, and semi-supervised learning models.

## Materials and methods

### Patient selection

This study was approved by the Institutional Review Board of our institution (Samsung medical center, IRB File No. 2022-11-073-001), and the requirement for informed consent was waived owing to its retrospective nature. Further, this study was conducted in accordance with the principles of the Declaration of Helsinki. Initially, we identified 273 patients diagnosed with CMT confirmed to have a PMP22 duplication through genetic analysis at our neurology department and who underwent lower extremity MRI between September 2013 and December 2022. A radiologist with 6 years of experience in musculoskeletal radiology (BLINDED) reviewed and categorized patients as either ’mild’ or ’severe’ based on the degree of lower leg muscle fat infiltration using the five-point semi-quantitative scale described by Goutallier et al. [15] Cases with Goutallier grade 3 or 4 fat infiltration on T1-weighted images, indicating 50% or higher fatty degeneration, were categorized as ’severe.’ The MRI scans of 120 patients (60 with mild and 60 with severe fat infiltration) were used as a training set. Data were initially accessed for research purpose on May 2^nd^ 2023. Authors did not have access to information that could identify individual participants during or after data collection.

### MRI protocol

Images were obtained using a 3.0-T MRI system (Ingenia; Philips Healthcare, Best, Netherlands) with a 16-channel anterior coil and a posterior built-in coil. MRI protocols including the following sequences were obtained for the pelvic girdle, bilateral thighs, and lower legs to evaluate the lower extremity muscles: axial and coronal T1-weighted turbo spin echo sequences and axial T2 -weighted Dixon sequence. To acquire the axial T1-weighted turbo spin-echo sequence of the lower legs, the following parameters were used: repetition time, 612 ms; echo time, 11 ms; flip angle, 90°; number of signals averaged, 1; field of view, 284 × 379 mm; section thickness, 3 mm; gap, 11 mm; matrix size, 448 × 269; echo train length, 3; and number of slices, 32.

### Ground truth segmentation

A radiologist (BLINDED) first selected the axial T1-weighted image of the proximal lower leg at a level just inferior to the inferior margin of the popliteus muscle, where the muscle was no longer visualized. The axial image of the distal lower leg was selected at the level of the uppermost part of the gastrocnemius tendon, where the gastrocnemius muscle was no longer visualized. The axial images selected for the proximal and distal lower leg, based on anatomical landmarks, have been used in numerous studies employing MRI to evaluate lower leg muscles in patients with CMT [9, 24–26]. These two axial images, along with others at intermediate levels, were used to analyze each patient. A single rater (BLINDED) manually delineated the boundaries of the bilateral lower leg muscle compartments on each slice using ITK-SNAP software (http://www.itksnap.org, accessed December 30, 2020) under a radiologist’s supervision. The muscle compartments were categorized as follows: anterior (tibialis anterior and extensor digitorum longus), lateral (peroneus longus), deep posterior (tibialis posterior), and superficial (gastrocnemius and soleus). Boundaries were drawn to avoid the extramuscular fat tissue, bony cortex, and neurovascular bundles.

### Comparisons of models using different training data labeling strategies

**Fig 1:**
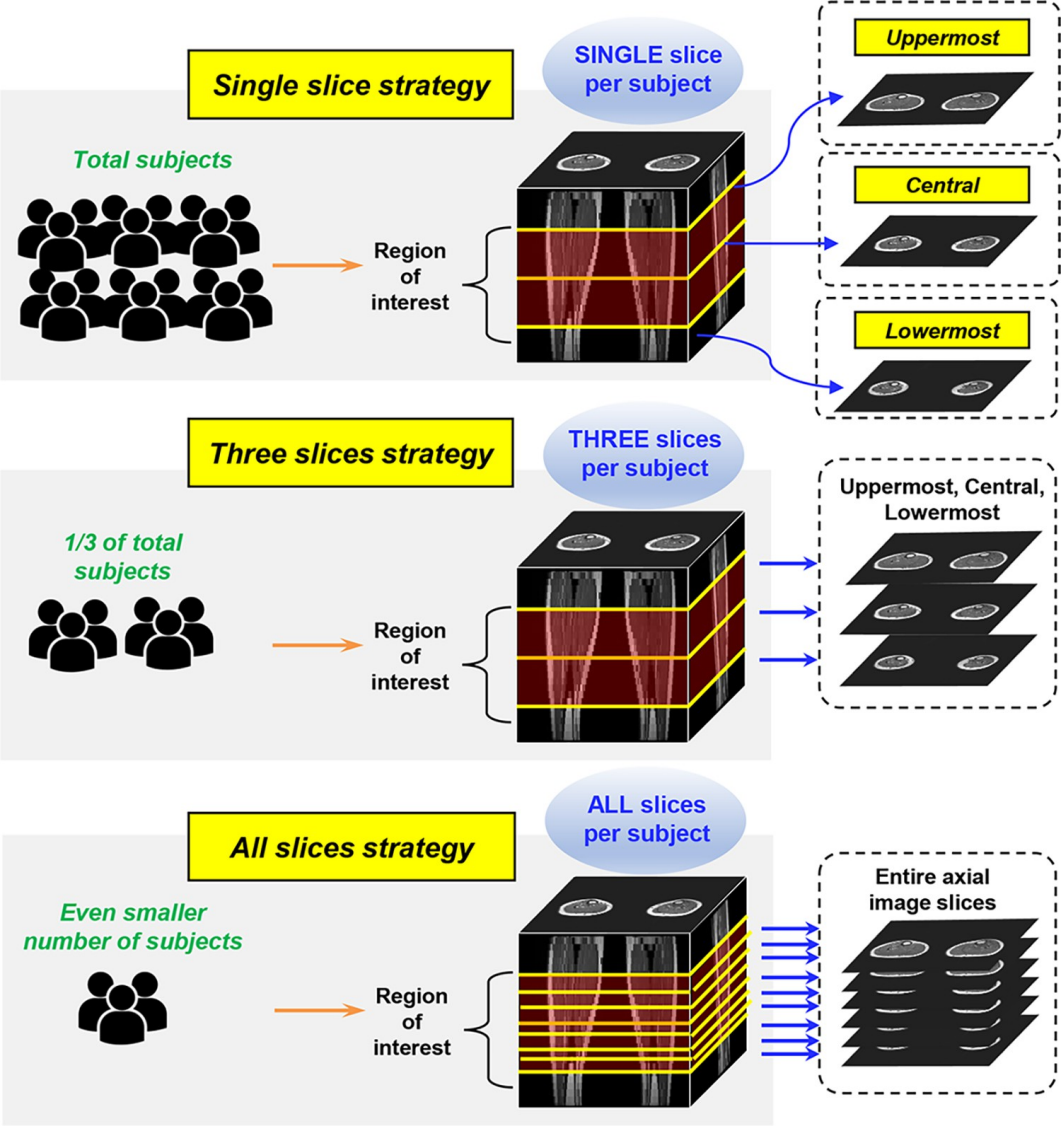
Data labeling strategies for ground truth segmentation of the lower leg MRI data. For “single slice strategy”, single image slice per subject was used to train the model to maximize the number of subjects included for training. The single image provided was chosen as either the uppermost, the central, or the lowermost axial T1-weighted images among segmented lower leg images, of which the strategy was named as “uppermost,” “central,” and “lowermost strategy,” respectively. The “three-slices strategy” utilized the uppermost, central, and lowermost image slices of each subject. The number of subjects used in this strategy was nearly one-third of that of the “single slice strategy,” given the same amount of data were used for training. The “all-slices strategy” utilized all the axial images of the lower legs per subject. Thus, the number of subjects used in this strategy was even smaller than that of the “three-slices strategy”.

We devised several strategies for labeling the MRI data for training models, considering inter-subject and inter-slice variations of the data. Intersubject variation is related to anatomical variation, as well as the degree of intramuscular fat infiltration in each subject, which is linked to the severity of denervation in patients with CMT. Interslice variation is related to variations in the substantially differing lower leg muscle compartment anatomy, depending on the proximal-to-distal level [27]. Specifically, we categorized the strategies as “single slice,” “three-slices,” and “all-slices strategies” (Fig 1). For the “single slice strategy,” a single image slice per subject was used to train the model to account for intersubject variation by maximizing the number of subjects that were included for training. The single image provided was chosen either as the uppermost, the central, or the lowermost axial T1-weighted images among segmented lower leg images, of which the strategy was named as “uppermost,” “central,” and “lowermost strategy,” respectively. The “three-slices strategy” utilized the uppermost, central, and lowermost image slices of each subject. The number of subjects used in this strategy was nearly one-third of that of the “single slice strategy,” given that the same amount of data were used for training. The “all-slices strategy” utilized all the axial images of the lower legs per subject. Thus, the number of subjects used in this strategy was smaller than that of the “three-slices strategy.” This strategy was devised to account for inter-slice variation by maximizing the number of proximal-to-distal levels of the axial images within the subjects.

### Comparison of models using different learning methods and different amount of training data

**Fig 2:**
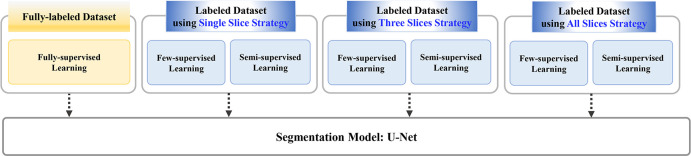
Outline of the research methodology. We compared muscle segmentation performances of fully-supervised learning models and few-supervised and semi-supervised learning models of three different data labeling strategies. The fully-supervised learning employed all labeled data for training, while few-supervised learning employed specific proportions of the labeled data. The semi-supervised learning model utilized a combination of labeled and unlabeled data during training.

We compared the muscle segmentation performance of the fully-supervised learning model and the few- and semi-supervised learning models of the three different data labeling strategies (Fig 2). Fully-supervised learning employs all the labeled data for training, whereas few-supervised learning employs specific proportions (2%, 5%, and 10%) of the labeled data. The semisupervised learning model utilizes a combination of labeled and unlabeled data during training; this method has been reported to demonstrate results comparable to those of fully supervised learning [28, 29]. After model training, we compared the muscle segmentation performances of the fully supervised learning model and the few-supervised and semi-supervised learning models of three different data labeling strategies.

### Image preprocessing

MRIs were converted into the TensorFlow TFRecords format for dataset loading. The 3D images were separated into 2D images and were subsequently cropped to 448 × 672 pixels along the y-axis and resized to half of the cropped size to accelerate computation and the effect of data augmentation. The intensities of the MRIs scans were normalized using mean and standard deviation of each slice, standardizing pixel intensities across images to have a mean of 0 and a standard deviation of 1.

### 2D U-net: 2D segmentation model

**Fig 3:**
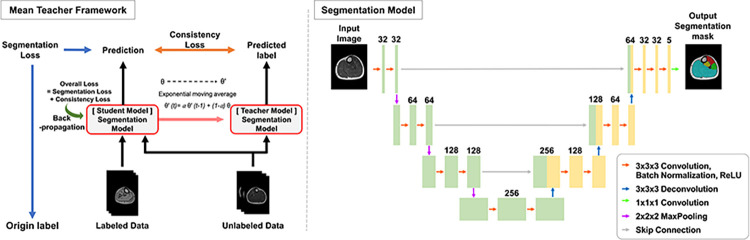
Mean teacher framework for semi-supervised model and the architecture of the U-net for lower leg muscle segmentation.

We used a 2D U-Net to segment the four muscle compartments on MRIs, as U-Net has been shown to provide promising medical image segmentation performance [30, 31]. U-Net is a fully convolutional network that consists of an encoder, a bottleneck, and a decoder (Fig 3). The encoder downsamples the input image to extract high-level features, while the decoder upsamples these features to match the original image resolution. Skip connections between the encoder and decoder enable the model to access low-level semantic information from the encoder, which again facilitates the generation of desired features by the decoder (S1 File).

### Semi-supervised deep learning model

We used the mean teacher classification model for semi-supervised learning [32, 33]. The mean-teacher model uses two neural networks, a student network, and a teacher network, to train on both labeled and unlabeled data. During training in the supervised phase, the student network was updated with both labeled and unlabeled data using gradient descent, whereas the teacher network’s weights remained fixed. In the unsupervised phase, both networks predict unlabeled data, and the predictions of the student network were compared with those of the teacher network. The goal was to minimize the difference between the two predictions, which helped the student network learn from the unlabeled data. At each step, the student network’s weights are updated with the teacher network’s weights, called exponential moving average (EMA) weight sharing [32, 34]. The EMA stabilizes training, maintains consistency, and aids the student model in better generalization.

### Implementation details

The dataset was divided into training and testing sets (80%:20%), assuming an equal number of labeled slices for each labeling strategy. We randomly sampled 2, 5%, and 10% of the labeled data as labeled sets and used the remaining labeled data as an unlabeled set (Table 1). The experiments were implemented using TensorFlow 2.6.0 and trained for 1000 epochs with a batch size 32 on two GTX 1080 Ti GPUs. Data augmentation using the albumentation library involved horizontal flipping, rotation within (-30, 30) degrees, scaling with a factor in the range (-0.5~0.3) for both height and width, and shifting with a factor in the range (-0.2, 0.2) [13].

**Table 1:** Number of images used for the training and testing of the models with different labeling strategies.

**Training set**	**Learning method**	**Fully-supervised**	**Few-supervised and Semi-supervised**								
	Labeling strategy	NA	Single slice strategy (Uppermost/Central/Lowermost)			Three-slices			All-slices		
	Percentage of the labeled data used for training	100%	2%	5%	10%	2%	5%	10%	2%	5%	10%
	Labeled data	966 (Mild 49/ Severe 49)	19 (Mild 9/ Severe 10)	47 (Mild 23/ Severe 24)	98 (Mild 49/ Severe 49)	18 (Mild 3/ Severe 3)	48 (Mild 8/ Severe 9)	99 (Mild 16/ Severe 17)	21 (Mild 1/ Severe 1)	43 (Mild 2/ Severe 2)	98 (Mild 4/ Severe 6)
	Unlabeled data	0	947	919	868	948	918	867	945	923	868
Testing set		224 (Mild 12/ Severe 13)									

Values represent the number of axial images used for the training and testing sets, which included bilateral lower legs.

Values in parentheses represent the number of subjects with mild or severe intramuscular fat infiltration.

NA, Not applicable.

The segmentation performance of each model was measured using the Dice coefficient [13], which is the spatial overlap between the predicted mask and ground truth:

$$Dice=\frac{2\times TP}{\left(TP+FP)+(TP+FN\right)}$$ (1)

In Eq 1, TP represents true positive predictions, FP represents false positive predictions, and FN represents false negative predictions. During training, we utilized the Dice loss function (1-Dice) and Adam optimizer with a learning rate of 1e-3.

The mean squared error was employed as the consistency loss to minimize the difference between the predictions of the student and teacher models in unsupervised learning.


$$L_{Total}=L_{supervised}+\lambda ∙L_{unsuperivsed}$$ (2)

$$\theta^{\prime }(t)=\alpha \theta^{\prime }(t-1)+(1-\alpha )\theta$$ (3)

In semi-supervised learning, the hyper-parameters comprised the unsupervised loss weight factor λ, and the EMA decay rate α. The unsupervised loss weight factor *γ* followed a sigmoid curve from 0 to 1. The model updated teacher model weights *θ*′ at training step *t* using Eq 3, with the updating rate governed by the EMA decay parameter α set to 0.999.

### Statistical analyses

We compared the performances of the segmentation models obtained using different labeling strategies by comparing the average Dice coefficients (ADC) of the four muscle compartments. First, pairwise comparisons were conducted among the few supervised learning models trained with a 10% labeled set with different labeling strategies (single slice [uppermost, central, or lowermost], three-slices, and all-slices). Pairwise comparisons between few-supervised and semi-supervised learning models of different strategies were performed to verify the benefits of the semi-supervised methods. Further comparisons were performed based on the proportion of labeled data used for training (2%, 5%, and 10%). Paired t-tests were employed, and the Bonferroni correction was applied for multiple comparisons. Statistical analyses were performed using SAS version 9.4 (SAS Institute, Cary, NC, USA), considering a *P*-value < 0.05 as statistically significant.

## Results

### Comparisons of models using different training data labeling strategies

**Fig 4:**
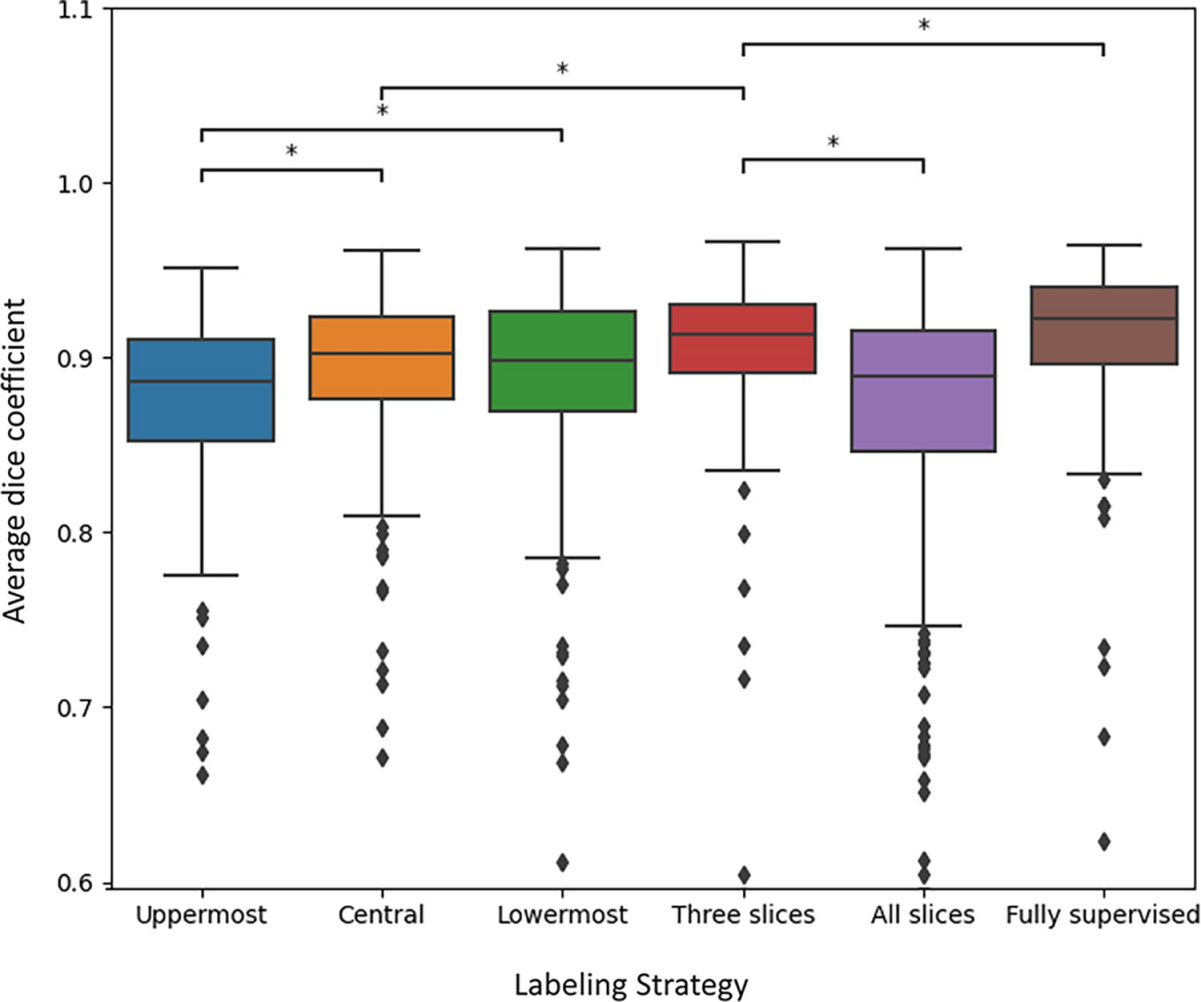
Boxplots for the comparisons of average dice coefficients among segmentation models using different training data labeling strategies. Each model was trained using 10% of the labeled data except for the fully-supervised model. * indicates statistical significance.

**Fig 5:**
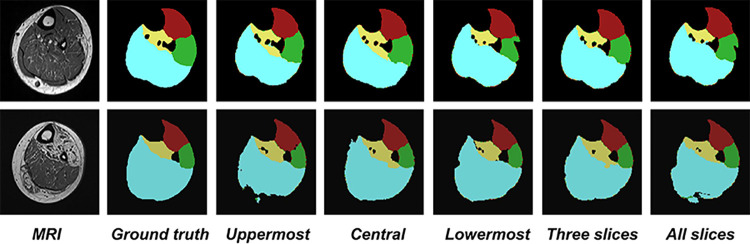
Examples of MRI, ground truth, and segmentation results of few-supervised learning models with 10% labeled data using different labeling strategies in mild (upper row) and severe (lower row) intramuscular fat infiltration in the lower leg. Red, green, yellow, and aqua colored regions represent anterior, lateral, deep, and superficial posterior compartments of the lower leg, respectively.

ADCs of the few-supervised learning models trained with 10% labeled set with single slice strategies were uppermost, 87.79±4.41%; central, 89.42±4.07%; and lowermost, 89.29±4.71% (mean ± standard deviation). Pairwise comparisons among the models using different single-slice strategies revealed that the ADCs of the central and lowermost slice strategies were significantly higher than those of the uppermost slice strategy (*p* < 0.0001; Figs 4 and 5). The ADCs of the three-slices strategy and all-slices strategy were 90.84±3.46% and 85.97±9.82%, respectively. Pairwise comparisons between the two models showed that the model trained using the three-slices strategy had a significantly higher ADC than that trained using the all-slices strategy (*p* < 0.0001). The model using the three-slices strategy also showed a significantly higher ADC than that using the central-slice strategy (*p* < 0.0001).

### Comparison of models using different learning methods and different amount of training data

Table 2 shows the results of pairwise comparison of the ADCs of few-supervised learning models using 2%, 5%, and 10% of the labeled data and semi-supervised learning models using 10% of the labeled data for training. Comparisons among few-supervised learning models using 2%, 5%, and 10% data sets revealed significantly improved segmentation performances as the amount of the labeled data for training increased, except for the comparison between models using central slice strategy with 5% and 10% data set for training. Pairwise comparisons between few-supervised and semi-supervised learning models using 10% of the labeled data showed significantly better segmentation performances of the semi-supervised models using slice central (Few- vs. Semi-supervised, 89.42±4.07% vs. 90.34±3.54%; *p* < 0.0001), lowermost slice strategy (Few- vs. Semi-supervised, 89.29±4.71% vs. 89.68±4.47%; *p* = 0.0081), three-slices strategy (Few- vs. Semi-supervised, 90.84±3.46% vs. 91.03±3.67%; *p* = 0.0293) and all-slices strategy (Few- vs Semi-supervised, 85.97±9.82% vs 89.71±4.23%; *p* < 0.0001). In contrast, the ADC of the few-supervised model using the uppermost slice strategy was significantly higher than that of the semi-supervised model (Few- vs Semi-supervised, 87.79±4.41% vs 91.03±3.67%; *p* = 0.0293). Further, the ADC of the fully-supervised model was 91.39±3.76%, which was significantly higher than that of the semi-supervised model using the three-slices strategy with 10% labeled set, which was the model that showed the highest segmentation performance (91.03±3.67%, *p* = 0.0246).

**Table 2:** Average dice coefficients of the few-supervised learning models using 2%, 5%, and 10% of the labeled data, and semi-supervised learning models using 10% of the labeled data for training.

Percentage of the data used for training	Single slice strategy						Three-slices strategy		All-slices strategy	
	Uppermost		Central		Lowermost					
	ADC	*p*	ADC	*p*	ADC	*p*	ADC	*p*	ADC	*p*
2% Few-supervised	81.68±8.82	2% vs 5% <0.0001*	82.63±10.57	2% vs 5% <0.0001*	81.69±8.90	2% vs 5% <0.0001*	79.71±13.00	2% vs 5% <0.0001*	53.87±21.72	2% vs 5% <0.0001*
5% Few-supervised	85.15±7.32	2% vs 10% <0.0001*	89.17±4.90	2% vs 10% <0.0001*	85.91±8.92	2% vs 10% <0.0001*	89.09±4.79	2% vs 10% <0.0001*	84.53±5.19	2% vs 10% <0.0001*
10% Few-supervised	87.79±4.41	5% vs 10% <0.0001*	89.42±4.07	5% vs 10% 0.6199	89.29±4.71	5% vs 10% <0.0001*	90.84±3.46	5% vs 10% <0.0001*	85.97±9.82	5% vs 10% 0.0004*
10% Semi-supervised	87.14±5.06	10% vs 10%^†^ <0.0001*	90.34±3.54	10% vs 10%^†^ <0.0001*	89.68±4.47	10% vs 10%^†^ <0.0081*	91.03±3.67	10% vs 10%^†^ <0.0293*	89.71±4.23	10% vs 10%^†^ <0.0001*

ADC, average Dice coefficients.

Values are the mean ± standard deviation (%).

* Statistically significant.

^†^10% Few-supervised model vs 10% Semi-supervised model.

## Discussion

Herein, we investigated the efficiency of several different training data labeling strategies and learning models to develop an automated segmentation model of the lower leg muscles in patients with CMT. Pairwise comparisons of ADCs were conducted for a few supervised learning models trained with 10% of labeled datasets using different labeling strategies. The results showed that the model using the three-slices strategy had segmentation performances significantly superior to those of the models using other labeling strategies. For the three-slices strategy, labeling was performed on the three axial image slices per subject: the uppermost, central, and lowermost image slices within the designated boundaries of the lower legs. The superior segmentation performance of the three-slices strategy compared with that of the single-slice strategies may be attributable to the fact that the three-slices strategy better accounts for interslice variation. Regardless of the specific level of images provided for training, a single image slice may not be sufficient to achieve optimal muscle segmentation performance in the lower legs, owing to the substantial differences in muscle compartment anatomy depending on the proximal-to-distal level [27]. The superior segmentation performance of the three-slices strategy compared with the all-slices strategy, which was trained with images from more variable proximal-to-distal levels and fewer subjects, might be due to the three-slices strategy’s better accounting for intersubject variation. It might therefore be necessary for the training dataset of the automated segmentation model to have a certain degree of inter-subject variation to obtain optimal segmentation performance by adjusting to the anatomical variation as well as the varying patterns and degrees of intramuscular fat infiltration of the lower leg muscles.

We evaluated the effect of unlabeled data utilization on the enhancement of muscle segmentation performance by comparing the performance of the models using few-supervised and semisupervised learning. Pairwise comparisons between the two models, for which we used the mean teacher model, showed significantly improved segmentation performance when semisupervised learning was used in most models. Leveraging unlabeled data in semi-supervised learning benefits the model in terms of learning the data distribution and improving the generalization and predictions of labeled data [35]. Training solely on labeled data may increase sensitivity to data noise, whereas leveraging unlabeled data enables the model to learn diverse patterns and variations, making it more robust to data noise [35]. With its potential to boost performance, the semisupervised model has shown promising results in image-segmentation tasks [33, 36]. Our results further suggest that the use of unlabeled data for training can substantially improve the network performance of lower leg muscle segmentation in patients with CMT. A semi-supervised model using the three-slices strategy with a 10% labeled set showed ADC of 91.03±3.67%, which we believe is comparable to the performance of the fully-supervised model (91.39±3.76%).

Our study has several limitations. First, the muscle segmentation models developed in our study were trained and tested on MRIs from a single institution, necessitating validation with MRIs from different acquisition parameters. We acknowledge that variability in MRI protocols across institutions may influence the generalizability of the model. Second, models were developed using two-dimensional T1-weighted spin-echo images. A three-dimensional gradient echo Dixon-based MRI sequence with a larger number of axial images and thinner slices may be more valuable for automated muscle segmentation [3, 4]. However, using such a sequence would require significantly more time and effort in the ground truth segmentation process because of the higher number of axial images per designated anatomical region compared to two-dimensional T1-weighted spin-echo images. We believe that our results can guide the design of a strategy for ground-truth segmentation using a three-dimensional MRI sequence. Third, we did not evaluate more advanced deep learning models, such as the nnU-net or residual U-Net [37, 38], which might have the potential to enhance the segmentation performance of the lower leg muscles in patients with CMT. Finally, potential bias may be present due to manual segmentations being performed by a single rater.

In conclusion, we identified that performing ground truth segmentation using a three-slice-based strategy, with labeling of the uppermost, central, and lowermost image slices of a subset of subjects within the designated anatomical volume of the lower legs in patients with CMT, was the most efficient strategy for building an automated muscle segmentation of axial T1-weighted MRI. In addition, our results indicate that a semi-supervised model using unlabeled data with a mean teacher model significantly improves the performance of the model. Our findings may enhance the efficiency of the ground truth segmentation process in future studies on automated muscle segmentation, addressing a significant challenge in developing an automated segmentation model. Based on the conclusions drawn about the most efficient method for ground truth segmentation in this study, we aim to develop an automated segmentation model of axial three-dimensional gradient echo Dixon-based MRI sequences in the lower legs of patients with CMT in future studies.

## Supporting information

S1 File 2D U-Net for segmentation of the four muscle compartments on MRI. (DOCX)

S2 File Raw data of the segmentation models. (PDF)

## Abbreviations

CMT: Charcot-Marie-Tooth disease
MRI: magnetic resonance imaging
ADC: average dice coefficient
EMA: exponential moving average
