# Supplementary material for: Efficient data labeling strategies for automated muscle segmentation in lower leg MRIs of Charcot-Marie-Tooth disease patients
Source: PLoS One. 2024 Sep 6;19(9):e0310203. doi: 10.1371/journal.pone.0310203 (PMC11379393; doi:10.1371/journal.pone.0310203)
Supplement: S1 File — (DOCX) [file pone.0310203.s001.docx]

**Supplementary Material:**

We used a 2D U-Net to segment the four muscle compartments in the MRI scans. This is a fully convolutional network containing an encoder, bottleneck module, and decoder. The encoder downsamples the input image to extract high-level features, while the decoder upsamples these features to match the original image resolution. Furthermore, the decoder concatenates the up-sampled features with the corresponding low-level features from the encoder using skip connections. These skip connections enable the model to access essential low-level semantic information from the encoder, thus facilitating the generation of the desired features by the decoder. The encoder comprised four convolution blocks, each featuring repeated convolutional layers, followed by batch normalization (BN), rectified linear units (ReLU), and a max-pooling layer. The decoder is composed of four deconvolution blocks, each with repeated deconvolutional layers, followed by the BN and ReLU. The output of the last decoder block was passed to a 1×1 convolution layer to map each component feature vector to the desired number of classes.
