# Supplementary material for: Efficient data labeling strategies for automated muscle segmentation in lower leg MRIs of Charcot-Marie-Tooth disease patients
Source: PLoS One. 2024 Sep 6;19(9):e0310203. doi: 10.1371/journal.pone.0310203 (PMC11379393; doi:10.1371/journal.pone.0310203)
Supplement: S2 File — (PDF) [file pone.0310203.s002.pdf]

Dice Score

Image	Models	Model A	Model B_Slice				
			Upper 2%	Upper 5%	Upper 10%	Center 2%	Center 5%
1		86.104%	78.445%	84.106%	84.154%	70.146%	75.653%
2		86.995%	83.635%	90.075%	83.656%	48.414%	83.836%
3		87.005%	82.214%	85.977%	86.190%	76.854%	83.432%
4		89.091%	87.453%	88.890%	86.784%	66.224%	85.474%
5		89.662%	82.421%	89.096%	88.734%	73.463%	87.185%
6		92.695%	86.534%	92.636%	90.237%	71.125%	91.249%
7		91.685%	82.163%	87.561%	87.700%	85.557%	89.295%
8		93.264%	84.585%	90.925%	90.079%	64.463%	90.109%
9		87.172%	82.891%	84.775%	88.372%	83.311%	87.178%
10		88.627%	88.542%	87.486%	90.385%	67.151%	87.146%
11		91.958%	84.085%	88.522%	89.734%	87.059%	90.004%
12		92.013%	86.268%	89.076%	90.032%	71.996%	90.713%
13		92.581%	85.176%	89.838%	89.413%	90.728%	91.055%
14		92.126%	85.762%	80.116%	89.316%	65.013%	91.435%
15		94.029%	82.748%	90.914%	89.659%	86.373%	94.570%
16		93.572%	84.872%	82.748%	91.476%	72.549%	94.370%
17		93.515%	82.444%	90.298%	88.172%	87.678%	93.427%
18		95.541%	87.957%	87.669%	90.900%	75.959%	92.872%
19		94.393%	79.639%	87.560%	84.211%	82.942%	93.768%
20		95.012%	87.552%	90.245%	89.199%	83.158%	94.210%
21		91.931%	86.818%	87.180%	90.308%	89.777%	90.824%
22		94.213%	86.912%	93.380%	93.332%	81.989%	92.900%
23		91.779%	73.831%	86.146%	86.968%	89.167%	91.807%
24		93.622%	85.885%	89.091%	88.764%	75.506%	91.593%
25		94.836%	75.075%	86.163%	87.125%	89.156%	92.823%
26		91.803%	83.021%	86.702%	85.149%	70.767%	92.349%
27		93.489%	78.650%	84.537%	88.225%	91.578%	91.963%
28		93.088%	80.781%	83.097%	87.121%	67.609%	90.355%
29		94.899%	69.780%	69.154%	86.739%	74.458%	90.303%
30		93.311%	79.241%	80.418%	84.818%	68.084%	91.795%
31		93.976%	70.961%	70.138%	83.048%	79.086%	87.645%
32		93.319%	79.104%	76.385%	83.223%	72.170%	90.373%
33		84.098%	78.929%	87.092%	84.945%	76.013%	82.007%
34		87.724%	82.391%	90.704%	81.863%	76.525%	86.041%
35		86.318%	76.872%	90.984%	88.111%	80.183%	88.451%
36		91.325%	81.517%	89.752%	84.147%	80.684%	89.301%
37		89.715%	81.772%	89.752%	86.485%	81.920%	90.335%
38		93.301%	86.285%	92.138%	83.811%	83.504%	91.611%
39		88.910%	75.911%	87.201%	83.663%	77.796%	88.540%
40		90.366%	86.282%	89.513%	86.438%	83.729%	90.583%

41	94.715%	88.009%	92.665%	90.958%	88.329%	94.468%
42	93.988%	90.451%	90.872%	87.990%	88.107%	93.809%
43	93.946%	89.582%	90.775%	88.382%	88.435%	94.152%
44	94.121%	88.791%	88.082%	83.778%	86.494%	93.547%
45	94.031%	87.012%	92.729%	90.641%	90.249%	93.181%
46	87.461%	83.158%	84.483%	83.892%	87.074%	89.141%
47	92.616%	84.597%	90.586%	90.249%	93.529%	93.618%
48	92.069%	86.542%	90.045%	91.016%	90.603%	91.528%
49	91.646%	86.531%	89.811%	90.043%	91.644%	90.877%
50	92.250%	84.437%	89.996%	91.753%	89.791%	90.497%
51	94.406%	87.810%	90.861%	89.995%	93.176%	92.517%
52	93.807%	81.504%	87.131%	91.871%	91.300%	89.231%
53	95.891%	88.421%	93.704%	92.531%	92.947%	94.537%
54	92.997%	85.805%	87.823%	92.903%	89.730%	89.870%
55	95.665%	86.875%	87.948%	89.947%	92.054%	93.382%
56	95.296%	81.875%	86.629%	92.844%	91.768%	94.722%
57	95.045%	78.351%	88.081%	88.746%	90.463%	92.060%
58	94.209%	84.272%	86.447%	92.514%	90.512%	92.891%
59	85.672%	84.217%	87.232%	85.860%	80.088%	87.354%
60	85.359%	77.172%	85.378%	84.609%	82.549%	88.905%
61	85.536%	80.739%	85.910%	86.117%	84.709%	88.890%
62	86.229%	80.133%	86.005%	86.143%	85.675%	90.415%
63	85.583%	78.962%	83.208%	82.186%	84.721%	88.944%
64	87.676%	79.042%	85.749%	86.490%	84.916%	91.503%
65	85.581%	75.038%	78.445%	79.294%	84.048%	88.981%
66	87.446%	77.235%	82.412%	84.190%	82.790%	91.134%
67	88.123%	76.538%	78.216%	81.064%	89.568%	89.780%
68	90.052%	78.470%	78.035%	83.176%	89.575%	93.138%
69	88.099%	75.081%	78.813%	79.671%	89.730%	91.463%
70	90.357%	80.088%	75.930%	85.451%	88.521%	92.963%
71	90.325%	73.082%	82.480%	82.514%	90.955%	93.194%
72	91.919%	83.533%	77.727%	86.613%	89.618%	91.419%
73	91.387%	70.458%	82.840%	85.230%	91.925%	92.937%
74	93.157%	80.795%	82.178%	83.529%	90.150%	91.497%
75	90.987%	74.396%	80.006%	82.944%	92.202%	93.863%
76	92.768%	80.653%	81.099%	85.624%	90.684%	91.689%
77	91.169%	79.738%	79.058%	87.294%	90.030%	91.630%
78	91.872%	44.565%	63.721%	87.142%	61.464%	84.149%
79	92.788%	82.969%	82.017%	89.744%	87.903%	91.878%
80	89.980%	40.085%	60.351%	84.485%	53.495%	79.430%
81	93.001%	71.454%	84.089%	86.526%	88.561%	89.531%
82	88.529%	44.361%	51.595%	82.154%	62.905%	82.816%
83	93.526%	71.359%	78.891%	86.573%	89.352%	88.727%

84	89.629%	45.546%	67.230%	81.065%	79.625%	85.336%
85	92.177%	60.069%	76.891%	84.056%	87.192%	86.631%
86	89.187%	45.996%	58.337%	75.584%	74.269%	86.772%
87	90.749%	83.888%	86.274%	89.133%	84.168%	90.586%
88	80.814%	78.764%	76.544%	75.118%	80.875%	83.211%
89	89.613%	86.109%	81.481%	89.965%	89.873%	88.514%
90	81.553%	82.500%	77.595%	78.421%	76.854%	85.833%
91	89.315%	88.737%	78.818%	88.696%	89.054%	91.319%
92	84.677%	78.294%	78.781%	83.124%	44.041%	86.915%
93	89.806%	89.253%	80.341%	83.927%	90.226%	91.950%
94	85.715%	79.668%	78.486%	81.190%	83.578%	86.767%
95	89.794%	86.631%	83.810%	84.412%	93.703%	90.601%
96	86.796%	78.862%	82.978%	80.929%	67.720%	89.338%
97	92.842%	86.385%	91.632%	92.287%	81.957%	88.313%
98	92.480%	81.249%	90.788%	91.478%	77.580%	90.618%
99	95.406%	86.662%	93.876%	94.159%	81.728%	91.068%
100	92.719%	82.082%	91.894%	91.633%	78.654%	90.133%
101	90.920%	86.553%	92.236%	90.981%	84.482%	89.083%
102	91.324%	87.601%	90.000%	89.499%	85.455%	90.997%
103	91.881%	85.000%	91.468%	90.942%	86.503%	92.586%
104	93.813%	86.000%	90.533%	90.529%	85.085%	93.383%
105	92.210%	86.662%	91.612%	91.859%	88.056%	92.963%
106	93.802%	88.358%	90.549%	89.493%	88.913%	92.244%
107	91.801%	75.945%	88.388%	89.624%	89.031%	92.252%
108	90.479%	91.570%	89.841%	88.287%	90.861%	92.348%
109	92.382%	81.197%	89.475%	90.250%	89.437%	92.627%
110	93.387%	90.121%	86.767%	88.195%	91.995%	91.133%
111	94.242%	79.142%	89.911%	90.374%	92.509%	92.609%
112	92.003%	88.340%	83.800%	86.904%	88.961%	87.759%
113	94.947%	79.793%	86.300%	88.646%	91.685%	90.980%
114	95.308%	88.569%	83.393%	88.090%	92.066%	88.071%
115	93.645%	78.184%	83.485%	84.726%	89.948%	91.318%
116	94.253%	86.296%	79.893%	85.478%	88.886%	87.893%
117	96.009%	73.600%	83.281%	83.325%	91.214%	91.895%
118	95.345%	83.170%	83.299%	82.185%	89.194%	86.397%
119	91.703%	85.746%	90.309%	90.836%	84.702%	92.142%
120	92.550%	86.972%	93.115%	93.441%	83.018%	89.861%
121	91.889%	86.805%	90.727%	90.078%	82.097%	90.847%
122	94.046%	85.314%	93.632%	93.887%	82.642%	90.193%
123	90.795%	86.849%	87.413%	88.123%	86.773%	90.381%
124	91.760%	83.193%	90.827%	92.401%	82.772%	91.086%
125	90.163%	85.372%	88.423%	88.953%	90.573%	92.631%
126	91.281%	87.687%	89.943%	91.901%	87.132%	91.661%

127	90.759%	88.490%	89.367%	91.231%	92.782%	94.256%
128	93.214%	87.931%	91.166%	93.485%	89.497%	93.630%
129	92.408%	90.135%	88.799%	87.852%	94.644%	94.132%
130	91.331%	86.157%	88.365%	93.019%	88.479%	89.363%
131	93.469%	85.630%	91.677%	91.577%	91.945%	93.513%
132	94.004%	88.504%	86.451%	91.518%	92.054%	91.630%
133	96.231%	83.142%	88.638%	92.115%	93.196%	92.507%
134	93.800%	88.855%	84.404%	87.792%	91.254%	91.911%
135	95.853%	79.981%	89.218%	92.519%	93.097%	94.394%
136	94.067%	87.443%	84.278%	88.689%	90.849%	90.740%
137	95.903%	81.888%	86.527%	92.435%	93.411%	92.344%
138	95.643%	86.673%	83.305%	87.715%	91.384%	91.720%
139	94.509%	64.156%	85.275%	90.616%	64.394%	71.534%
140	94.263%	80.974%	88.362%	91.720%	74.598%	85.862%
141	90.590%	70.603%	86.523%	88.250%	64.855%	76.037%
142	94.903%	81.889%	88.790%	89.000%	75.075%	84.551%
143	93.122%	75.773%	90.578%	92.114%	72.949%	87.421%
144	96.130%	84.423%	88.983%	89.678%	80.827%	88.280%
145	92.525%	77.536%	91.049%	91.582%	75.687%	84.832%
146	94.734%	84.284%	88.203%	91.217%	82.544%	88.281%
147	93.142%	82.360%	92.685%	92.121%	82.776%	92.310%
148	94.791%	85.963%	90.159%	91.302%	85.236%	89.606%
149	92.095%	79.938%	92.256%	89.354%	84.395%	92.585%
150	94.984%	85.735%	89.026%	90.419%	86.110%	90.085%
151	92.670%	84.836%	91.078%	88.535%	85.099%	92.676%
152	92.505%	86.875%	90.895%	89.260%	88.140%	92.070%
153	93.320%	86.927%	87.624%	89.104%	89.585%	91.740%
154	92.873%	89.968%	88.949%	90.250%	88.219%	91.538%
155	90.502%	83.451%	84.136%	87.110%	89.731%	91.452%
156	94.076%	87.287%	90.053%	90.756%	87.981%	91.698%
157	92.132%	84.549%	85.997%	86.342%	89.839%	91.355%
158	92.759%	84.333%	86.694%	88.598%	88.531%	89.929%
159	90.087%	79.757%	85.666%	88.437%	90.538%	90.858%
160	93.244%	82.139%	83.288%	88.603%	87.622%	91.280%
161	95.825%	85.825%	84.004%	81.680%	94.284%	94.832%
162	95.383%	82.925%	79.322%	83.699%	87.944%	87.789%
163	87.676%	76.866%	87.291%	88.098%	81.354%	88.269%
164	91.515%	83.501%	89.948%	88.930%	87.469%	90.946%
165	89.448%	79.578%	87.276%	90.005%	83.507%	89.951%
166	90.737%	85.502%	87.888%	86.265%	87.894%	89.609%
167	91.229%	73.321%	91.573%	89.888%	83.556%	91.607%
168	90.815%	84.314%	85.862%	91.026%	89.820%	89.714%
169	91.757%	70.611%	89.356%	90.072%	84.233%	92.415%

170	90.327%	87.179%	81.504%	86.420%	92.129%	89.846%
171	93.430%	69.680%	90.260%	87.996%	83.925%	91.748%
172	90.543%	87.035%	80.979%	88.345%	90.824%	92.698%
173	93.745%	71.438%	90.039%	88.020%	85.015%	92.416%
174	93.065%	87.110%	86.019%	88.817%	90.309%	91.316%
175	93.295%	81.602%	92.245%	92.438%	80.252%	85.784%
176	92.173%	84.389%	93.256%	92.966%	79.481%	87.586%
177	93.639%	82.956%	92.145%	90.455%	84.045%	89.001%
178	91.517%	85.949%	91.351%	92.149%	84.583%	91.686%
179	92.482%	84.876%	92.802%	92.265%	85.015%	90.720%
180	94.086%	88.157%	93.591%	93.709%	86.535%	94.291%
181	92.539%	84.141%	90.588%	90.854%	85.313%	90.492%
182	94.501%	87.415%	93.077%	94.365%	88.870%	93.831%
183	91.552%	87.397%	91.178%	91.252%	88.615%	92.378%
184	95.931%	89.355%	92.793%	94.356%	92.046%	94.264%
185	93.185%	87.357%	91.855%	90.263%	91.786%	92.481%
186	94.832%	91.653%	88.429%	94.417%	92.787%	93.935%
187	92.147%	84.835%	90.173%	89.214%	92.405%	94.003%
188	94.530%	91.754%	93.085%	94.578%	93.326%	94.759%
189	94.398%	83.608%	89.714%	92.525%	93.569%	94.565%
190	95.072%	92.657%	91.264%	94.093%	93.454%	95.345%
191	95.280%	81.658%	87.874%	89.760%	93.227%	95.148%
192	95.824%	89.334%	90.423%	93.649%	95.407%	95.941%
193	95.440%	79.198%	86.000%	85.578%	92.568%	94.574%
194	95.887%	87.593%	86.883%	92.089%	94.521%	96.105%
195	85.793%	86.065%	85.278%	85.923%	77.529%	82.594%
196	86.554%	91.882%	92.362%	84.254%	68.258%	82.658%
197	88.360%	85.086%	85.342%	82.689%	78.326%	80.689%
198	88.528%	88.693%	92.910%	83.440%	79.127%	84.269%
199	88.302%	81.949%	88.068%	84.429%	80.068%	79.832%
200	91.888%	82.438%	90.774%	85.925%	68.404%	89.257%
201	89.577%	75.652%	90.156%	81.723%	73.478%	59.390%
202	91.508%	84.601%	91.663%	84.503%	71.017%	90.927%
203	88.353%	79.996%	83.680%	80.404%	84.775%	86.102%
204	87.378%	87.589%	89.790%	86.094%	84.212%	90.588%
205	87.943%	79.884%	80.265%	82.320%	86.810%	86.712%
206	91.131%	87.298%	91.621%	86.962%	74.588%	89.281%
207	91.002%	62.890%	79.809%	86.359%	75.157%	85.195%
208	91.628%	87.300%	89.731%	89.130%	74.002%	91.897%
209	90.197%	82.401%	88.093%	87.914%	76.798%	83.297%
210	91.089%	84.125%	87.333%	89.583%	84.911%	89.760%
211	87.903%	85.215%	86.229%	85.912%	81.542%	88.163%
212	88.798%	84.797%	85.188%	88.682%	80.467%	87.072%

213	87.017%	83.157%	89.186%	89.108%	82.802%	86.643%
214	85.957%	86.251%	84.847%	85.730%	79.974%	89.013%
215	85.654%	88.042%	88.831%	86.868%	89.376%	88.920%
216	81.538%	84.951%	81.635%	82.495%	83.614%	85.683%
217	88.007%	86.848%	88.399%	88.977%	83.683%	88.371%
218	84.492%	87.906%	85.961%	82.791%	87.702%	91.218%
219	88.963%	84.851%	87.605%	85.756%	86.202%	84.279%
220	86.951%	90.609%	84.996%	85.048%	91.106%	92.879%
221	90.777%	89.195%	89.434%	89.402%	90.402%	91.960%
222	85.125%	89.266%	80.270%	86.567%	90.829%	91.604%
223	89.860%	87.347%	87.307%	85.848%	89.968%	91.127%
224	86.914%	91.750%	82.041%	86.424%	93.375%	93.138%
225	89.318%	88.001%	85.762%	88.736%	89.985%	90.341%
226	87.277%	90.521%	79.879%	85.233%	92.457%	92.521%
227	89.360%	89.675%	84.244%	84.519%	87.515%	89.482%
228	90.260%	90.050%	80.116%	83.074%	93.349%	90.194%
229	93.593%	84.614%	84.467%	83.308%	92.916%	94.045%
230	94.238%	88.338%	81.587%	81.481%	92.315%	85.207%
231	87.094%	79.120%	82.790%	83.879%	91.184%	91.958%
232	94.700%	84.119%	83.796%	81.203%	90.753%	91.033%
233	62.349%	62.783%	66.696%	66.148%	70.218%	73.616%
234	68.335%	78.651%	74.788%	70.477%	57.733%	78.255%
235	72.299%	70.771%	76.255%	73.555%	82.849%	78.486%
236	73.449%	82.555%	81.651%	78.134%	60.861%	81.864%
237	84.026%	77.607%	87.202%	83.048%	79.917%	86.790%
238	81.507%	88.468%	89.937%	81.326%	74.080%	90.045%
239	84.502%	80.785%	88.909%	83.058%	80.968%	86.904%
240	86.051%	87.189%	91.257%	86.945%	75.762%	91.507%
241	89.470%	81.386%	90.098%	89.046%	82.096%	90.885%
242	88.696%	86.814%	90.895%	87.734%	74.550%	90.233%
243	88.690%	79.408%	83.909%	83.906%	85.228%	90.085%
244	91.559%	87.209%	90.800%	90.213%	73.967%	89.343%
245	90.741%	84.420%	86.182%	87.692%	86.089%	91.798%
246	93.075%	88.592%	91.856%	92.550%	87.184%	91.968%
247	87.829%	78.903%	83.919%	84.398%	84.541%	89.176%
248	91.967%	90.163%	86.169%	90.398%	88.687%	91.847%
249	92.096%	84.758%	86.004%	86.338%	87.658%	91.014%
250	92.481%	87.919%	92.160%	93.752%	89.160%	92.432%
251	94.750%	86.783%	87.298%	84.474%	92.814%	94.374%
252	94.917%	91.125%	92.304%	92.559%	92.688%	94.923%
253	94.538%	85.718%	83.559%	86.872%	92.099%	94.011%
254	94.633%	90.423%	88.000%	91.019%	89.111%	94.035%
255	95.039%	85.986%	82.723%	85.973%	91.353%	93.894%

256	94.666%	91.906%	88.822%	88.369%	92.054%	93.981%
257	88.026%	87.560%	87.522%	88.817%	78.555%	85.297%
258	89.967%	87.469%	88.832%	86.287%	53.992%	80.309%
259	85.316%	85.579%	82.046%	87.856%	68.973%	77.751%
260	91.835%	85.388%	91.359%	90.411%	68.009%	85.695%
261	83.662%	85.533%	78.503%	82.066%	73.976%	82.089%
262	92.389%	90.076%	91.729%	90.053%	77.802%	88.841%
263	91.229%	84.887%	87.518%	90.308%	85.490%	89.998%
264	91.458%	85.558%	84.145%	88.013%	79.146%	88.344%
265	89.066%	88.091%	88.542%	90.743%	85.993%	89.356%
266	94.105%	89.638%	89.497%	86.454%	76.936%	91.583%
267	91.513%	83.722%	82.972%	85.610%	86.935%	91.063%
268	92.843%	85.003%	86.563%	87.467%	85.137%	90.897%
269	91.649%	81.651%	82.585%	87.274%	84.683%	86.573%
270	91.770%	82.719%	84.500%	83.181%	85.475%	90.316%
271	89.565%	79.710%	76.166%	81.676%	84.099%	86.180%
272	89.114%	76.695%	76.388%	77.485%	82.218%	85.582%
273	90.342%	76.283%	76.305%	81.937%	82.566%	87.435%
274	93.447%	76.377%	80.265%	83.934%	74.376%	88.541%
275	92.672%	76.993%	81.646%	86.831%	87.088%	87.301%
276	93.519%	74.838%	78.089%	85.800%	82.983%	85.976%
277	92.808%	79.306%	90.172%	91.598%	73.708%	83.898%
278	93.946%	86.435%	92.063%	90.810%	80.860%	86.221%
279	92.365%	82.862%	91.158%	91.451%	77.853%	86.328%
280	94.971%	88.343%	93.274%	93.632%	85.338%	88.757%
281	92.839%	78.233%	90.508%	91.213%	76.659%	84.547%
282	94.571%	86.414%	90.691%	92.735%	83.173%	85.572%
283	93.420%	83.767%	90.181%	91.354%	79.670%	87.975%
284	92.106%	89.130%	93.162%	92.848%	86.270%	87.147%
285	94.094%	71.418%	92.062%	92.350%	83.006%	85.676%
286	94.366%	88.911%	93.218%	92.198%	87.294%	90.429%
287	94.030%	81.529%	90.616%	90.154%	88.915%	92.763%
288	93.467%	92.856%	92.735%	93.436%	91.667%	91.098%
289	94.052%	82.605%	88.272%	88.969%	89.899%	95.125%
290	94.184%	82.378%	92.444%	90.856%	91.813%	93.077%
291	91.755%	87.637%	85.080%	83.793%	91.784%	92.110%
292	94.857%	78.886%	88.778%	87.123%	92.175%	93.392%
293	89.610%	82.394%	88.177%	87.004%	83.558%	92.114%
294	87.276%	90.857%	89.084%	86.142%	89.457%	90.546%
295	90.260%	81.613%	88.614%	88.689%	81.793%	90.529%
296	90.801%	91.832%	91.499%	91.418%	90.198%	93.063%
297	93.203%	77.377%	89.103%	90.009%	83.551%	92.251%
298	92.829%	92.685%	91.621%	91.294%	91.472%	93.753%

299	93.856%	77.025%	87.188%	91.241%	84.249%	92.262%
300	93.040%	90.402%	82.553%	90.987%	87.053%	94.008%
301	94.164%	82.272%	84.887%	89.103%	88.692%	91.567%
302	92.844%	88.772%	89.314%	90.012%	88.856%	93.145%
303	94.730%	74.835%	81.461%	87.297%	90.080%	92.251%
304	92.778%	86.008%	86.092%	90.363%	89.794%	92.947%
305	93.555%	78.449%	82.661%	90.611%	88.775%	91.952%
306	92.124%	86.274%	82.933%	92.608%	88.354%	93.582%
307	94.342%	84.456%	81.807%	88.839%	91.139%	94.878%
308	94.201%	88.556%	85.948%	93.060%	90.511%	93.908%
309	93.978%	82.773%	73.518%	90.191%	89.820%	93.806%
310	93.449%	83.928%	81.115%	91.417%	84.447%	88.391%
311	94.139%	80.552%	77.733%	89.931%	87.502%	93.368%
312	94.227%	81.288%	80.042%	87.533%	84.280%	91.599%
313	84.712%	84.891%	86.361%	85.110%	82.267%	83.213%
314	85.742%	83.608%	84.778%	86.080%	75.420%	87.728%
315	85.640%	86.564%	84.589%	85.430%	83.151%	88.708%
316	86.260%	81.978%	83.377%	85.013%	79.786%	88.009%
317	84.357%	81.967%	82.919%	86.850%	87.013%	91.005%
318	85.958%	82.708%	81.571%	83.141%	85.353%	88.539%
319	83.004%	81.290%	76.463%	81.156%	84.270%	88.332%
320	86.759%	82.044%	80.018%	83.643%	85.491%	88.587%
321	86.700%	81.197%	71.326%	81.275%	87.301%	88.811%
322	86.371%	75.708%	78.378%	83.838%	82.439%	85.060%
323	91.305%	76.131%	72.245%	85.121%	89.620%	90.123%
324	88.525%	78.900%	79.677%	83.796%	78.886%	88.855%
325	92.766%	70.482%	71.428%	80.336%	84.921%	88.886%
326	93.739%	86.407%	77.828%	89.543%	87.028%	91.550%
327	91.004%	63.490%	70.362%	78.168%	83.837%	87.371%
328	94.959%	80.106%	80.985%	86.478%	82.843%	86.631%
329	91.972%	66.818%	84.475%	89.487%	75.872%	78.744%
330	91.487%	81.074%	85.917%	88.517%	81.321%	86.923%
331	92.129%	71.122%	84.447%	88.928%	75.166%	81.360%
332	92.713%	83.061%	90.039%	90.973%	79.752%	91.200%
333	95.085%	68.132%	87.996%	92.596%	73.351%	77.806%
334	94.344%	80.210%	86.414%	90.787%	82.790%	90.249%
335	95.502%	63.398%	78.927%	90.978%	70.763%	89.189%
336	94.302%	76.906%	87.915%	90.299%	79.263%	91.208%
337	95.449%	54.594%	77.176%	90.176%	72.000%	90.072%
338	92.899%	77.304%	85.034%	79.459%	68.378%	92.866%
339	93.810%	59.566%	79.148%	85.460%	70.523%	91.447%
340	93.726%	76.294%	78.733%	78.413%	67.777%	93.054%
341	91.910%	85.004%	92.054%	91.693%	76.664%	78.770%

342	92.599%	89.217%	92.897%	91.735%	83.388%	83.606%
343	93.007%	84.874%	92.910%	93.039%	78.941%	81.226%
344	94.348%	91.024%	94.327%	92.514%	84.531%	83.965%
345	94.166%	86.186%	94.021%	94.375%	82.375%	86.031%
346	94.832%	90.909%	94.367%	92.773%	88.135%	86.517%
347	93.045%	85.989%	93.666%	92.363%	80.140%	87.159%
348	93.768%	93.152%	93.048%	93.904%	88.080%	85.665%
349	93.920%	87.162%	93.985%	92.854%	87.806%	89.124%
350	94.743%	91.487%	94.638%	92.809%	90.468%	88.637%
351	93.000%	86.802%	89.502%	90.576%	86.962%	89.298%
352	92.543%	88.102%	90.890%	93.468%	88.810%	89.694%
353	95.799%	88.450%	85.823%	91.769%	90.441%	93.049%
354	94.584%	84.575%	86.825%	91.987%	87.495%	91.654%
355	92.803%	84.868%	87.154%	90.417%	90.976%	91.234%
356	93.616%	84.447%	86.495%	92.049%	90.911%	92.000%
357	92.080%	86.390%	83.519%	91.904%	90.158%	92.109%
358	93.212%	80.643%	82.006%	89.199%	83.531%	90.027%
359	87.655%	82.132%	74.233%	88.393%	89.460%	90.651%
360	94.662%	77.395%	83.061%	88.667%	88.186%	91.103%
361	92.292%	81.837%	67.481%	84.650%	91.279%	90.461%
362	93.336%	84.036%	76.993%	86.501%	86.387%	89.960%
363	88.466%	76.066%	71.840%	80.615%	88.255%	90.471%
364	92.928%	78.933%	75.320%	87.204%	85.343%	87.561%
365	93.902%	83.067%	74.241%	82.620%	89.481%	90.367%
366	89.981%	59.796%	68.785%	81.407%	78.322%	83.788%
367	94.403%	72.375%	66.932%	80.799%	89.508%	86.980%
368	92.837%	61.915%	67.149%	68.217%	74.088%	76.625%
369	85.863%	51.258%	56.495%	78.445%	80.724%	79.770%
370	85.057%	56.219%	67.872%	67.443%	68.654%	76.698%
371	89.589%	67.593%	88.232%	89.986%	56.152%	60.565%
372	92.546%	79.388%	87.854%	86.531%	73.395%	75.732%
373	92.806%	72.111%	91.007%	92.922%	62.649%	72.198%
374	91.683%	81.802%	92.244%	90.840%	77.686%	82.511%
375	92.066%	75.910%	92.657%	94.472%	64.213%	75.121%
376	92.001%	80.129%	90.696%	90.801%	77.331%	81.774%
377	93.717%	79.788%	91.988%	93.914%	68.756%	79.832%
378	91.723%	85.511%	92.600%	91.333%	78.492%	81.057%
379	94.038%	83.579%	91.878%	92.257%	77.562%	84.999%
380	94.323%	86.476%	93.133%	92.756%	83.062%	83.913%
381	91.512%	81.228%	90.792%	91.795%	80.238%	84.393%
382	94.283%	88.981%	91.352%	93.705%	84.922%	86.882%
383	95.302%	86.362%	90.116%	94.465%	84.528%	89.813%
384	93.661%	87.256%	90.383%	90.592%	85.456%	86.038%

385	92.416%	84.335%	88.072%	91.906%	86.681%	91.562%
386	92.212%	86.007%	87.572%	90.314%	89.425%	90.093%
387	91.940%	86.696%	86.137%	88.375%	89.249%	92.128%
388	91.425%	85.273%	82.885%	88.866%	86.811%	89.809%
389	89.193%	85.718%	82.252%	85.710%	86.184%	90.364%
390	88.947%	80.651%	82.984%	86.325%	89.166%	89.599%
391	88.556%	78.600%	72.549%	82.480%	86.739%	87.717%
392	88.263%	78.476%	80.396%	85.142%	87.479%	88.919%
393	83.316%	75.418%	69.663%	80.083%	84.470%	86.940%
394	87.877%	75.353%	77.129%	82.796%	85.957%	89.491%
395	91.231%	79.984%	71.687%	82.360%	89.361%	90.782%
396	89.434%	70.895%	74.774%	83.531%	81.947%	86.656%
397	91.285%	75.338%	70.029%	77.820%	88.731%	88.191%
398	86.751%	65.019%	70.494%	80.091%	77.410%	81.992%
399	94.490%	90.690%	94.041%	94.925%	78.607%	93.688%
400	89.321%	92.742%	90.960%	88.473%	83.373%	92.474%
401	94.952%	91.006%	94.601%	95.093%	78.606%	95.577%
402	93.700%	92.784%	94.728%	92.541%	88.259%	95.558%
403	94.070%	91.605%	94.298%	95.095%	83.525%	95.967%
404	94.475%	94.582%	94.182%	92.978%	90.864%	96.443%
405	94.112%	93.867%	92.160%	93.906%	90.095%	95.746%
406	95.272%	92.053%	93.013%	94.491%	92.885%	96.141%
407	95.325%	93.835%	90.846%	91.488%	91.154%	93.233%
408	96.176%	95.208%	93.807%	95.076%	94.126%	96.354%
409	96.392%	91.324%	90.923%	91.523%	92.698%	95.169%
410	96.180%	93.712%	91.240%	94.616%	95.634%	96.000%
411	95.612%	88.872%	91.106%	91.695%	89.456%	95.776%
412	96.107%	91.468%	90.290%	92.897%	95.105%	95.277%
413	96.194%	87.667%	88.413%	86.987%	94.116%	95.535%
414	96.256%	89.607%	89.541%	91.627%	94.295%	95.013%
415	88.770%	83.286%	86.964%	89.184%	83.428%	87.693%
416	89.356%	85.908%	85.640%	89.506%	84.789%	87.962%
417	90.418%	87.871%	85.267%	88.805%	83.507%	86.081%
418	91.152%	83.331%	86.068%	90.166%	87.011%	87.859%
419	91.299%	88.557%	83.405%	89.832%	77.272%	87.752%
420	90.765%	85.196%	84.301%	89.169%	84.048%	88.074%
421	92.963%	85.215%	84.129%	88.644%	87.166%	88.144%
422	93.192%	79.451%	84.433%	90.767%	78.448%	87.891%
423	94.243%	87.883%	76.332%	89.613%	90.355%	89.879%
424	91.302%	69.614%	78.088%	87.138%	80.605%	87.809%
425	93.542%	86.832%	79.611%	91.435%	89.410%	90.374%
426	91.321%	67.260%	68.691%	83.594%	77.296%	85.813%
427	93.059%	78.537%	81.809%	89.055%	89.005%	89.823%

428	91.613%	67.999%	76.517%	86.651%	68.148%	88.276%
429	93.833%	73.026%	87.340%	88.974%	90.823%	90.322%
430	93.179%	68.803%	70.041%	91.060%	36.275%	90.749%
431	94.846%	69.964%	84.256%	92.529%	82.990%	90.401%
432	94.743%	60.745%	71.272%	86.775%	37.865%	87.894%
433	94.561%	57.450%	81.713%	87.781%	81.085%	87.573%
434	94.465%	60.187%	62.419%	80.470%	33.354%	84.228%
435	86.512%	87.711%	85.218%	84.538%	68.593%	82.306%
436	84.225%	68.085%	63.786%	81.584%	28.161%	59.532%
437	86.327%	84.538%	85.178%	82.009%	45.535%	75.640%
438	88.924%	68.489%	80.797%	85.806%	39.731%	84.838%
439	90.199%	82.874%	85.879%	85.869%	65.911%	76.329%
440	88.364%	51.214%	80.450%	84.278%	41.551%	88.250%
441	88.164%	80.174%	78.097%	88.913%	54.326%	86.595%
442	89.569%	56.964%	79.093%	85.576%	47.162%	85.803%
443	88.089%	83.128%	80.372%	85.295%	85.259%	89.228%
444	89.621%	61.039%	70.570%	86.364%	57.418%	89.014%
445	90.120%	76.706%	84.399%	88.248%	70.964%	91.166%
446	91.012%	31.313%	49.957%	84.525%	45.464%	88.542%
447	90.628%	76.008%	81.272%	86.836%	56.224%	92.421%
448	89.873%	48.046%	43.370%	77.678%	44.371%	83.978%

							M
**Center 10%**	**Lower 2%**	**Lower 5%**	**Lower 10%**	**Upper 2%**	**Upper 5%**	**Upper 10%**	**Center 2%**
79.901%	68.955%	77.925%	85.446%	84.679%	87.470%	82.864%	75.057%
85.477%	61.677%	84.722%	86.439%	88.673%	92.247%	85.277%	78.979%
82.468%	69.912%	81.224%	89.335%	83.513%	87.017%	84.717%	69.958%
85.447%	74.335%	84.247%	84.743%	88.234%	91.889%	87.644%	79.181%
87.981%	68.557%	76.326%	88.694%	84.801%	89.388%	88.469%	70.708%
89.318%	72.995%	84.868%	88.756%	88.451%	94.084%	90.966%	85.946%
87.088%	73.908%	76.249%	88.441%	85.162%	89.844%	89.620%	78.014%
87.354%	77.206%	85.781%	87.744%	87.384%	93.286%	91.258%	85.268%
86.961%	75.814%	80.195%	88.539%	86.753%	90.687%	90.195%	82.064%
86.806%	79.906%	88.305%	88.844%	88.344%	92.167%	91.397%	89.533%
88.367%	77.359%	87.478%	89.749%	85.528%	92.013%	90.501%	84.310%
88.475%	81.187%	91.051%	91.263%	88.468%	92.491%	92.116%	90.372%
92.026%	82.976%	87.904%	92.426%	84.082%	92.402%	91.523%	86.568%
88.479%	84.483%	91.035%	91.975%	85.462%	88.496%	90.807%	91.162%
93.654%	91.108%	89.377%	94.782%	87.008%	92.592%	91.515%	90.298%
90.232%	89.002%	94.116%	94.447%	82.673%	92.030%	92.340%	91.098%
90.981%	92.721%	93.786%	93.851%	85.303%	91.898%	91.329%	91.693%
91.713%	92.083%	94.133%	94.467%	83.050%	91.981%	91.107%	91.680%
90.199%	93.048%	94.394%	95.647%	84.401%	88.882%	90.471%	90.954%
91.109%	88.907%	94.091%	94.515%	81.723%	91.767%	90.314%	92.368%
90.594%	81.942%	84.512%	90.162%	83.977%	89.410%	91.193%	90.356%
93.808%	83.865%	83.963%	87.867%	86.588%	93.043%	92.993%	83.338%
90.950%	87.793%	87.932%	89.576%	76.251%	85.988%	88.026%	91.751%
91.611%	84.144%	85.617%	86.265%	84.534%	89.493%	92.192%	85.030%
91.827%	92.285%	91.873%	91.703%	76.584%	85.717%	88.244%	90.068%
91.446%	85.957%	87.317%	90.272%	84.094%	90.325%	89.179%	85.058%
92.661%	90.849%	93.105%	92.455%	74.279%	87.476%	86.296%	91.251%
86.872%	83.218%	94.359%	94.834%	84.429%	85.683%	85.872%	88.201%
89.064%	82.768%	93.558%	92.643%	69.506%	78.831%	83.687%	88.880%
86.421%	78.833%	94.855%	93.652%	77.744%	80.515%	85.900%	90.116%
87.534%	89.515%	92.688%	91.468%	64.759%	75.902%	81.553%	86.466%
82.791%	82.098%	93.089%	93.989%	72.156%	73.931%	83.961%	86.849%
84.377%	73.107%	72.049%	88.582%	87.128%	90.842%	81.292%	86.676%
85.721%	77.429%	78.908%	85.561%	85.707%	90.452%	88.988%	90.001%
84.910%	76.303%	83.222%	88.192%	88.633%	92.247%	85.629%	87.837%
88.571%	80.571%	86.043%	86.489%	88.187%	91.078%	89.316%	90.361%
93.990%	77.957%	80.692%	88.260%	88.120%	89.503%	85.823%	90.619%
88.644%	82.392%	88.550%	88.037%	89.192%	91.049%	82.377%	91.900%
89.919%	81.499%	83.664%	86.132%	83.796%	86.338%	84.545%	87.863%
84.355%	84.377%	89.507%	88.532%	87.437%	89.538%	85.505%	89.933%

92.674%	90.982%	94.204%	93.850%	87.526%	88.183%	86.959%	93.571%
90.028%	90.615%	93.505%	93.419%	90.725%	92.036%	86.687%	93.951%
93.429%	90.311%	93.511%	93.526%	84.722%	88.480%	82.724%	92.782%
85.442%	90.849%	93.664%	93.530%	88.077%	90.058%	81.491%	91.931%
95.361%	85.292%	89.457%	91.158%	90.302%	90.595%	92.339%	92.433%
89.045%	83.217%	83.962%	89.856%	82.672%	85.485%	86.166%	90.711%
93.636%	87.816%	89.263%	90.473%	90.450%	89.279%	90.853%	93.094%
91.399%	88.305%	88.657%	91.296%	86.536%	89.822%	89.385%	92.441%
91.532%	86.605%	89.963%	89.281%	89.472%	88.513%	88.816%	93.338%
91.310%	86.306%	90.452%	92.674%	84.969%	87.019%	90.173%	90.264%
93.078%	85.899%	91.121%	90.824%	90.306%	89.560%	90.865%	94.489%
88.517%	90.421%	89.088%	92.227%	85.431%	85.042%	91.206%	92.657%
94.098%	89.328%	91.184%	91.547%	91.154%	88.605%	92.144%	93.650%
90.335%	87.825%	91.541%	92.368%	87.245%	85.332%	89.763%	91.867%
92.876%	90.336%	95.019%	94.587%	88.595%	85.795%	88.841%	92.706%
89.239%	92.562%	94.662%	94.566%	85.419%	79.623%	91.121%	91.482%
92.284%	89.975%	94.670%	94.357%	84.217%	82.447%	88.539%	91.351%
89.081%	90.474%	94.302%	93.862%	85.419%	82.742%	88.079%	91.372%
90.587%	70.399%	56.467%	83.002%	82.076%	87.298%	86.559%	86.578%
81.683%	68.008%	46.308%	81.698%	85.826%	86.667%	84.946%	89.894%
91.459%	76.698%	66.557%	85.369%	78.349%	85.732%	86.432%	90.189%
86.323%	73.894%	58.854%	84.903%	85.997%	87.784%	85.605%	91.552%
89.357%	78.132%	72.810%	88.623%	72.010%	81.308%	85.113%	92.614%
88.314%	72.248%	55.282%	84.384%	83.424%	85.691%	85.679%	91.113%
86.833%	79.236%	75.264%	87.491%	73.207%	78.021%	82.473%	91.620%
87.692%	74.236%	65.532%	85.918%	75.159%	83.401%	84.481%	89.415%
88.136%	80.378%	87.177%	89.899%	72.609%	79.478%	84.491%	91.575%
88.696%	80.259%	76.268%	89.681%	78.074%	85.003%	85.767%	91.815%
88.351%	84.539%	88.615%	91.092%	69.043%	78.805%	83.142%	91.684%
88.060%	85.293%	83.263%	90.670%	76.387%	85.258%	86.118%	92.189%
89.147%	86.323%	90.750%	93.136%	66.755%	78.244%	83.978%	93.385%
88.770%	89.569%	90.753%	93.574%	75.608%	85.811%	84.288%	92.023%
90.561%	89.337%	91.006%	93.481%	68.454%	81.956%	86.319%	93.121%
88.966%	92.538%	94.094%	93.160%	75.846%	84.341%	83.279%	92.569%
85.487%	90.958%	93.532%	92.287%	70.949%	82.086%	83.909%	91.023%
88.712%	92.246%	93.894%	93.189%	75.908%	85.568%	82.968%	92.662%
90.261%	89.701%	91.862%	92.654%	78.649%	83.603%	85.571%	87.256%
84.360%	80.072%	90.618%	89.950%	71.632%	61.288%	83.897%	83.206%
90.010%	88.914%	90.880%	92.235%	83.878%	85.673%	88.721%	86.951%
84.088%	79.389%	93.454%	92.531%	68.036%	61.011%	81.921%	80.404%
89.309%	88.787%	90.811%	91.101%	82.225%	82.667%	87.354%	87.640%
80.923%	85.425%	92.004%	89.661%	68.039%	51.047%	80.455%	81.281%
89.151%	90.370%	92.209%	92.254%	77.330%	74.969%	84.478%	89.048%

81.671%	85.789%	90.516%	90.722%	68.397%	68.226%	80.763%	82.163%
84.906%	89.818%	91.012%	91.413%	71.229%	70.787%	80.715%	86.713%
81.121%	88.122%	90.654%	89.825%	71.601%	63.153%	80.485%	84.754%
89.259%	73.060%	72.987%	84.394%	81.827%	88.891%	89.929%	83.422%
80.354%	77.811%	87.855%	88.456%	81.219%	80.435%	72.663%	88.668%
89.110%	85.054%	87.508%	84.369%	77.364%	83.813%	84.258%	84.951%
81.602%	84.219%	86.454%	88.923%	82.103%	80.949%	73.369%	89.315%
86.609%	78.740%	89.084%	89.552%	79.761%	84.710%	84.711%	87.875%
82.144%	81.948%	87.167%	89.489%	79.011%	84.159%	77.411%	90.129%
88.017%	86.263%	91.361%	88.390%	79.170%	84.437%	79.813%	87.604%
82.304%	78.965%	89.686%	89.923%	77.482%	81.824%	77.690%	90.517%
85.124%	88.473%	89.493%	91.191%	80.692%	85.113%	79.380%	90.883%
87.067%	67.746%	90.695%	91.337%	79.528%	84.478%	78.565%	89.577%
91.962%	84.992%	83.739%	89.601%	86.881%	93.258%	91.793%	86.330%
90.312%	73.029%	81.305%	83.479%	84.097%	89.559%	91.821%	82.274%
94.702%	82.123%	79.156%	89.429%	86.291%	94.310%	92.663%	87.811%
90.914%	76.455%	83.277%	78.612%	83.621%	88.268%	93.001%	83.282%
91.870%	84.214%	86.238%	88.779%	87.519%	92.571%	90.641%	89.273%
90.425%	81.609%	85.059%	86.019%	87.977%	92.218%	90.702%	88.165%
92.616%	87.551%	87.358%	89.768%	88.424%	92.497%	92.419%	91.018%
92.962%	84.355%	85.828%	86.771%	89.009%	92.770%	92.897%	90.661%
91.675%	88.081%	89.600%	91.899%	87.844%	93.005%	91.263%	91.450%
91.439%	87.635%	87.807%	89.284%	90.724%	90.555%	87.692%	92.095%
90.330%	90.745%	92.319%	92.533%	88.279%	90.890%	92.135%	93.300%
88.795%	90.788%	91.443%	90.991%	90.466%	87.356%	86.674%	93.801%
90.160%	88.676%	91.764%	92.616%	91.041%	88.945%	91.260%	92.965%
89.728%	90.616%	93.098%	92.339%	90.948%	85.751%	88.195%	93.944%
89.978%	93.137%	93.222%	93.602%	92.131%	89.293%	90.743%	94.465%
86.855%	91.089%	92.658%	92.509%	88.243%	83.433%	86.389%	89.405%
90.608%	92.974%	93.839%	94.250%	87.937%	84.676%	83.998%	89.708%
85.137%	94.523%	94.145%	94.110%	87.337%	84.249%	87.316%	88.182%
88.549%	95.269%	94.056%	93.913%	87.647%	86.152%	85.571%	90.064%
81.764%	92.517%	93.192%	94.136%	84.807%	81.987%	83.274%	85.486%
92.726%	93.343%	94.942%	94.192%	84.090%	83.127%	82.488%	88.463%
84.104%	91.965%	94.809%	95.013%	82.790%	84.448%	87.055%	83.769%
91.836%	83.265%	81.281%	89.350%	87.149%	89.653%	90.421%	87.515%
90.998%	79.640%	83.107%	85.596%	86.886%	91.359%	92.063%	85.188%
90.447%	79.708%	84.053%	89.278%	86.623%	90.885%	91.031%	87.518%
91.624%	74.893%	82.897%	84.606%	86.585%	91.126%	93.548%	84.932%
88.368%	82.434%	87.372%	90.028%	88.179%	88.866%	89.319%	90.341%
91.059%	76.026%	82.871%	83.704%	87.445%	90.421%	92.298%	87.065%
89.890%	84.970%	88.809%	91.208%	88.380%	89.702%	90.106%	91.412%
90.345%	82.246%	84.449%	87.770%	89.728%	88.726%	92.428%	90.379%

91.540%	90.380%	93.079%	93.801%	91.581%	90.792%	91.378%	92.980%
92.997%	86.548%	90.201%	91.440%	90.296%	90.579%	93.857%	90.815%
91.489%	91.226%	93.096%	93.352%	92.268%	89.875%	91.080%	95.376%
89.924%	85.621%	89.637%	89.480%	89.525%	88.221%	91.679%	89.926%
90.794%	89.569%	92.667%	92.867%	93.126%	89.324%	92.689%	93.404%
91.598%	89.602%	92.081%	92.048%	92.376%	90.323%	93.898%	92.659%
92.280%	91.741%	95.660%	95.731%	90.542%	87.191%	93.582%	94.827%
92.532%	93.223%	94.490%	94.314%	89.985%	88.061%	90.652%	91.293%
93.799%	85.789%	95.283%	95.080%	88.315%	88.215%	92.639%	94.042%
92.765%	93.765%	94.623%	93.906%	90.077%	88.763%	91.363%	91.953%
92.593%	91.953%	94.930%	94.861%	86.685%	86.328%	91.242%	93.587%
92.601%	94.277%	95.043%	95.140%	88.179%	87.841%	89.805%	90.757%
86.377%	63.455%	58.114%	78.206%	75.574%	84.105%	93.132%	73.613%
93.174%	60.661%	75.958%	77.975%	83.437%	88.129%	93.800%	78.202%
86.256%	65.928%	61.928%	80.405%	80.286%	86.765%	93.808%	74.622%
89.333%	64.184%	77.560%	83.238%	83.242%	89.241%	93.836%	80.322%
91.304%	68.187%	70.182%	84.027%	82.453%	91.305%	93.352%	81.153%
93.722%	76.602%	81.669%	84.170%	88.081%	90.270%	93.802%	85.939%
89.977%	69.995%	70.433%	86.098%	83.214%	90.224%	93.509%	81.426%
90.682%	75.632%	81.912%	87.138%	87.203%	89.906%	93.460%	86.871%
91.100%	75.078%	84.404%	88.827%	88.260%	92.365%	91.373%	87.085%
91.573%	80.909%	82.422%	86.244%	88.385%	90.926%	91.639%	88.925%
91.157%	77.206%	86.195%	89.148%	88.976%	91.069%	92.279%	88.003%
93.667%	83.778%	86.822%	89.001%	87.945%	90.816%	93.427%	89.015%
91.376%	81.877%	90.207%	89.529%	89.104%	91.035%	93.385%	87.838%
93.866%	88.963%	88.946%	89.984%	90.430%	90.787%	91.493%	91.036%
92.958%	88.809%	76.550%	89.942%	87.767%	90.408%	91.152%	90.333%
91.663%	87.179%	91.064%	91.528%	89.357%	86.555%	87.268%	91.642%
91.176%	87.722%	88.671%	88.579%	87.104%	87.317%	89.986%	89.847%
91.210%	90.384%	89.848%	90.131%	85.515%	84.266%	88.738%	90.758%
91.343%	87.369%	88.800%	89.422%	84.422%	86.568%	89.165%	89.528%
89.422%	90.286%	91.125%	91.297%	82.076%	85.709%	87.838%	89.111%
91.212%	88.572%	88.718%	89.704%	83.402%	84.523%	88.971%	90.123%
88.815%	89.481%	92.577%	92.914%	81.009%	83.026%	85.627%	88.236%
94.072%	93.951%	95.053%	95.354%	85.464%	86.995%	81.760%	94.217%
88.685%	90.841%	94.836%	94.917%	81.090%	79.481%	82.546%	87.845%
88.987%	74.926%	79.709%	85.814%	88.676%	91.370%	89.200%	85.962%
89.517%	84.522%	85.343%	84.447%	85.635%	91.647%	92.038%	88.457%
87.762%	78.048%	82.406%	87.047%	87.722%	90.305%	87.419%	86.304%
87.439%	86.951%	88.519%	88.701%	88.534%	91.729%	88.794%	90.779%
89.331%	81.535%	92.189%	91.676%	79.667%	91.370%	90.111%	81.409%
89.305%	88.611%	91.254%	89.932%	89.549%	92.383%	90.427%	90.350%
89.646%	79.038%	94.559%	94.287%	81.704%	91.960%	89.640%	91.273%

87.311%	89.816%	94.290%	91.848%	90.310%	90.439%	88.818%	91.728%
90.173%	78.332%	94.582%	94.357%	80.073%	92.295%	87.862%	91.002%
87.775%	92.027%	94.530%	94.568%	87.186%	90.122%	87.420%	92.770%
90.796%	78.500%	95.254%	95.086%	82.746%	91.330%	87.531%	90.873%
88.662%	91.219%	95.083%	95.255%	90.034%	91.455%	86.999%	91.680%
93.381%	79.781%	80.851%	84.142%	86.068%	90.571%	92.121%	87.161%
92.828%	68.088%	85.325%	86.308%	88.593%	93.235%	91.257%	90.648%
93.437%	79.706%	82.504%	86.390%	88.878%	92.386%	92.725%	90.713%
91.957%	69.644%	85.366%	88.030%	87.583%	92.447%	92.330%	90.752%
93.789%	83.922%	85.057%	85.916%	88.506%	92.729%	93.000%	90.874%
94.566%	75.872%	87.176%	88.091%	89.543%	93.787%	94.634%	92.580%
92.820%	85.416%	87.329%	87.288%	88.292%	90.863%	91.393%	92.536%
93.923%	81.462%	85.576%	87.754%	89.173%	93.415%	93.852%	95.401%
91.970%	89.673%	89.315%	88.829%	89.906%	90.556%	91.362%	93.462%
94.572%	87.673%	90.163%	91.347%	90.216%	93.152%	94.436%	95.599%
93.608%	90.193%	90.949%	90.441%	86.038%	90.712%	90.593%	92.129%
94.441%	89.300%	93.351%	93.072%	91.157%	91.144%	94.323%	94.969%
93.019%	91.810%	92.106%	91.643%	86.483%	86.816%	92.307%	93.981%
94.878%	91.166%	93.042%	93.055%	91.528%	91.103%	94.357%	94.621%
93.940%	94.554%	94.048%	94.179%	83.801%	86.222%	91.793%	93.465%
94.442%	91.372%	93.803%	94.122%	90.825%	89.843%	93.237%	94.358%
92.124%	94.555%	95.109%	94.110%	80.400%	83.200%	89.011%	93.638%
95.779%	94.147%	95.382%	95.385%	89.852%	91.999%	89.977%	95.469%
91.864%	94.250%	93.820%	93.908%	80.549%	78.452%	85.515%	92.419%
95.701%	92.798%	94.232%	95.288%	87.043%	90.031%	90.436%	93.159%
92.147%	59.794%	81.381%	87.530%	86.241%	88.033%	82.865%	78.511%
92.283%	70.820%	74.495%	78.930%	87.444%	92.462%	82.629%	80.423%
92.276%	61.176%	81.184%	85.206%	86.884%	89.017%	82.465%	82.796%
92.990%	67.351%	76.883%	76.990%	87.551%	91.808%	81.732%	80.262%
87.965%	70.944%	72.843%	87.122%	87.225%	90.301%	81.460%	82.269%
93.507%	62.127%	68.802%	71.492%	88.573%	91.352%	82.288%	83.564%
91.080%	69.472%	61.614%	86.939%	84.676%	92.719%	81.760%	83.432%
91.578%	67.975%	79.293%	86.421%	88.685%	90.096%	81.774%	87.716%
92.581%	78.765%	77.468%	89.619%	81.861%	89.512%	82.245%	85.411%
91.714%	74.707%	88.342%	89.182%	89.003%	91.582%	82.609%	87.137%
89.058%	85.851%	87.426%	87.804%	78.001%	88.787%	83.793%	87.551%
92.357%	77.801%	79.431%	86.498%	89.769%	90.664%	84.546%	86.040%
92.341%	72.906%	65.660%	86.869%	83.765%	89.627%	85.414%	88.186%
94.149%	79.391%	90.318%	91.507%	87.502%	89.992%	86.248%	90.084%
91.140%	76.342%	76.851%	86.776%	86.971%	91.039%	89.041%	75.517%
88.904%	74.951%	85.678%	84.062%	87.656%	90.715%	88.750%	87.072%
90.205%	73.552%	74.784%	85.884%	89.030%	89.075%	86.425%	81.678%
88.602%	75.037%	84.899%	83.086%	86.424%	90.250%	87.761%	84.064%

90.820%	77.578%	79.929%	84.607%	90.572%	90.317%	90.398%	81.603%
85.609%	75.662%	84.542%	85.287%	88.243%	87.716%	84.696%	84.377%
89.543%	80.137%	82.681%	86.614%	88.643%	88.223%	88.033%	87.480%
82.675%	71.600%	83.284%	82.934%	85.647%	83.601%	83.182%	85.575%
90.663%	84.486%	84.718%	87.838%	88.937%	86.655%	90.990%	85.075%
83.745%	74.736%	86.508%	86.510%	88.293%	85.101%	84.147%	86.663%
90.088%	86.896%	87.452%	88.104%	87.933%	88.754%	89.342%	86.239%
87.859%	79.055%	86.260%	86.704%	88.082%	83.542%	85.085%	89.758%
90.478%	87.662%	90.784%	89.695%	88.595%	90.385%	90.553%	87.966%
85.542%	75.110%	86.685%	88.192%	87.293%	83.603%	80.332%	88.861%
91.461%	88.347%	90.284%	87.469%	88.076%	88.031%	88.416%	88.788%
85.163%	84.087%	91.980%	92.072%	90.381%	83.612%	81.239%	92.212%
88.640%	89.293%	88.843%	87.629%	84.422%	86.626%	87.722%	90.686%
87.627%	85.078%	92.151%	91.543%	90.888%	84.023%	80.614%	91.699%
88.457%	84.607%	89.993%	88.273%	88.198%	89.735%	86.501%	89.329%
89.236%	89.397%	94.496%	93.330%	88.187%	83.427%	81.576%	92.438%
93.556%	92.547%	94.783%	91.706%	87.105%	82.916%	86.808%	93.086%
89.328%	85.235%	93.534%	93.571%	85.917%	85.068%	83.667%	88.289%
88.745%	94.137%	92.298%	90.411%	83.880%	81.661%	84.006%	91.264%
83.697%	91.646%	94.317%	93.144%	86.190%	84.529%	87.002%	88.715%
67.114%	67.243%	74.061%	70.420%	79.881%	75.271%	58.807%	80.081%
71.343%	63.632%	78.502%	66.851%	83.906%	78.596%	66.856%	82.670%
73.190%	66.780%	83.310%	80.100%	84.835%	82.402%	67.977%	86.351%
76.604%	62.987%	76.691%	82.934%	87.145%	83.370%	73.582%	82.744%
82.395%	82.072%	83.080%	90.719%	90.908%	90.830%	81.449%	89.052%
81.114%	74.875%	85.440%	86.796%	86.772%	89.140%	79.478%	85.525%
84.610%	76.885%	82.559%	86.838%	86.869%	88.963%	84.148%	86.991%
91.058%	80.595%	86.556%	86.752%	86.972%	90.027%	84.425%	88.101%
85.891%	80.878%	83.363%	89.299%	90.464%	90.777%	88.579%	89.424%
88.631%	76.649%	87.113%	87.099%	87.305%	90.822%	85.586%	89.492%
85.345%	86.376%	86.275%	87.275%	87.659%	84.571%	85.804%	91.431%
89.199%	75.486%	87.307%	86.531%	86.354%	89.524%	88.908%	89.394%
88.360%	86.362%	88.508%	88.584%	89.157%	86.977%	88.316%	92.252%
90.951%	84.594%	89.634%	90.078%	90.226%	90.319%	91.447%	90.821%
86.888%	85.757%	88.349%	86.042%	89.013%	86.940%	87.821%	90.746%
91.590%	88.776%	89.935%	90.177%	91.700%	89.499%	90.997%	91.286%
90.504%	87.973%	90.757%	89.857%	89.933%	88.268%	89.287%	91.763%
90.850%	89.426%	91.856%	90.737%	92.454%	92.309%	92.689%	92.203%
90.486%	91.845%	94.747%	93.702%	90.668%	89.349%	88.259%	94.330%
92.484%	94.040%	94.631%	93.615%	92.552%	93.120%	92.171%	94.153%
88.688%	91.318%	94.396%	93.669%	85.981%	85.952%	84.494%	92.322%
93.059%	93.692%	94.834%	93.836%	89.509%	91.190%	88.781%	93.263%
90.040%	92.995%	94.467%	94.065%	85.139%	86.527%	82.671%	92.848%

93.053%	93.978%	94.652%	93.702%	88.479%	90.841%	84.784%	92.259%
82.649%	71.511%	63.951%	78.541%	88.762%	86.412%	79.963%	87.073%
86.827%	62.157%	62.271%	61.159%	86.844%	87.294%	85.397%	87.482%
85.689%	64.481%	55.860%	81.720%	88.280%	84.559%	78.883%	87.229%
91.353%	64.575%	63.036%	73.563%	87.816%	88.976%	86.746%	88.105%
83.599%	68.370%	74.150%	73.107%	87.396%	82.418%	77.846%	85.361%
91.599%	67.006%	76.718%	67.877%	87.694%	91.373%	89.017%	89.376%
90.368%	83.636%	63.804%	83.514%	85.982%	84.343%	85.130%	88.475%
89.427%	68.304%	78.710%	84.522%	86.027%	87.423%	85.648%	92.040%
91.506%	82.893%	87.880%	86.938%	84.867%	81.847%	84.588%	89.726%
92.918%	68.578%	88.569%	88.459%	87.532%	89.791%	89.204%	92.669%
90.641%	88.268%	89.987%	84.143%	80.911%	80.249%	83.927%	88.479%
91.241%	75.206%	85.461%	89.276%	86.010%	86.841%	85.627%	90.183%
89.822%	83.588%	87.164%	88.228%	79.108%	77.827%	85.005%	82.323%
89.371%	84.944%	89.235%	91.987%	83.537%	86.219%	79.805%	91.479%
88.938%	84.676%	90.308%	87.580%	71.172%	71.991%	78.997%	81.542%
81.594%	81.728%	89.803%	90.156%	77.626%	81.474%	77.596%	86.867%
87.145%	86.644%	90.471%	86.286%	72.628%	73.474%	80.142%	83.905%
85.333%	81.276%	92.771%	93.193%	76.716%	79.888%	82.063%	87.810%
88.066%	87.390%	92.881%	91.178%	73.727%	77.977%	84.068%	82.534%
86.839%	86.389%	92.797%	92.032%	73.952%	79.211%	81.479%	87.640%
92.311%	75.166%	70.600%	84.226%	85.436%	92.523%	92.734%	72.653%
93.988%	73.030%	76.583%	81.990%	86.384%	92.754%	92.866%	77.421%
92.623%	72.018%	75.085%	85.301%	85.646%	92.500%	92.455%	77.020%
94.141%	78.796%	78.558%	84.446%	89.134%	93.144%	92.587%	78.530%
92.590%	72.213%	77.508%	86.037%	85.922%	92.181%	92.112%	77.053%
93.148%	79.585%	81.331%	80.979%	84.783%	90.325%	92.721%	79.299%
92.113%	78.758%	78.307%	89.328%	87.971%	92.247%	91.875%	79.820%
92.710%	84.884%	86.687%	84.122%	88.763%	92.654%	93.139%	82.042%
90.503%	74.571%	78.436%	88.354%	85.189%	92.822%	91.001%	81.543%
92.823%	85.343%	88.279%	88.880%	91.121%	93.166%	93.011%	88.948%
92.306%	84.874%	91.683%	94.777%	86.664%	88.475%	88.944%	88.484%
91.708%	89.261%	92.831%	93.123%	91.924%	91.170%	89.256%	88.671%
93.557%	86.514%	91.161%	96.181%	90.989%	89.517%	89.008%	90.323%
95.023%	91.103%	95.219%	93.566%	90.798%	92.207%	87.675%	88.736%
91.481%	88.472%	91.819%	93.230%	83.998%	86.627%	84.204%	89.747%
92.678%	82.261%	94.422%	93.418%	87.680%	90.529%	80.171%	91.479%
88.940%	74.667%	76.681%	90.827%	91.240%	87.780%	86.307%	89.921%
84.851%	66.768%	89.805%	90.674%	92.673%	89.694%	83.952%	90.531%
90.283%	71.974%	83.245%	91.761%	87.226%	89.450%	87.175%	87.495%
91.270%	76.062%	90.371%	90.605%	91.087%	90.964%	87.556%	90.236%
93.183%	75.181%	83.440%	92.421%	88.108%	87.308%	88.852%	88.300%
92.614%	79.048%	91.318%	91.254%	91.123%	90.811%	88.253%	92.066%

92.892%	78.215%	83.718%	93.033%	88.373%	88.631%	88.847%	90.061%
92.768%	79.550%	90.947%	91.955%	87.504%	89.491%	89.042%	87.468%
90.911%	80.572%	89.145%	91.692%	90.339%	88.870%	88.113%	91.573%
92.084%	80.440%	91.749%	92.576%	84.352%	89.139%	88.894%	82.291%
92.559%	86.814%	92.921%	92.637%	89.543%	89.457%	88.785%	93.688%
91.542%	86.377%	92.049%	92.869%	87.022%	84.489%	89.352%	90.129%
93.014%	83.973%	86.405%	91.936%	89.018%	86.289%	88.145%	89.633%
92.435%	86.108%	93.244%	92.935%	86.425%	85.706%	89.185%	87.695%
92.778%	90.405%	94.752%	93.766%	90.363%	86.413%	89.604%	92.951%
93.686%	89.591%	95.161%	94.004%	88.090%	86.200%	88.291%	90.898%
92.702%	91.361%	94.571%	93.464%	85.370%	80.786%	86.699%	91.061%
92.028%	84.080%	94.365%	93.993%	85.352%	81.065%	86.661%	86.409%
91.637%	93.629%	94.311%	93.198%	87.268%	79.643%	84.628%	91.372%
90.872%	90.785%	94.876%	94.234%	85.750%	78.620%	84.500%	82.652%
86.011%	64.280%	76.191%	80.896%	87.390%	87.934%	85.169%	89.106%
86.750%	64.384%	76.174%	80.762%	87.646%	87.509%	84.623%	86.076%
86.907%	60.012%	82.044%	84.757%	85.537%	85.862%	83.984%	90.782%
85.779%	69.155%	82.762%	82.682%	84.647%	85.996%	84.750%	86.781%
86.642%	63.529%	82.265%	85.501%	81.147%	83.658%	82.859%	90.816%
82.893%	75.075%	84.652%	86.765%	85.206%	84.733%	84.160%	85.850%
85.229%	68.370%	84.553%	86.607%	76.181%	81.234%	80.917%	92.044%
84.334%	75.146%	82.559%	83.428%	83.774%	84.303%	82.976%	87.887%
89.269%	73.620%	87.091%	88.398%	76.902%	81.159%	83.216%	88.834%
84.672%	77.916%	85.317%	86.476%	79.870%	82.916%	82.054%	85.366%
90.328%	85.033%	92.718%	93.127%	75.397%	82.527%	84.216%	92.996%
86.220%	82.791%	87.421%	92.434%	76.558%	82.875%	78.812%	86.487%
87.476%	85.169%	91.950%	92.083%	76.611%	82.890%	79.694%	90.305%
90.488%	86.877%	94.719%	94.246%	78.702%	86.303%	81.292%	88.956%
84.140%	77.575%	91.668%	91.387%	72.472%	79.919%	80.740%	84.849%
90.909%	88.906%	92.546%	95.190%	80.302%	84.198%	84.234%	88.061%
88.126%	61.387%	60.652%	84.037%	76.403%	87.923%	92.376%	74.576%
88.202%	75.524%	77.419%	83.316%	76.023%	87.939%	89.866%	79.552%
89.880%	67.401%	69.732%	86.284%	78.650%	88.769%	92.827%	78.193%
92.692%	70.865%	82.488%	85.337%	81.933%	90.124%	91.996%	83.306%
91.447%	68.261%	63.493%	90.179%	77.205%	92.187%	93.176%	77.433%
92.317%	79.337%	85.154%	88.977%	78.789%	91.182%	92.440%	88.081%
93.919%	71.314%	82.511%	91.874%	75.160%	91.659%	92.098%	80.350%
94.302%	72.693%	86.317%	89.818%	74.686%	89.765%	92.435%	88.964%
94.654%	73.494%	73.082%	88.170%	75.966%	91.137%	90.703%	82.710%
92.328%	76.519%	87.872%	89.352%	70.285%	82.879%	90.593%	86.389%
93.570%	72.953%	80.331%	89.769%	76.491%	90.396%	90.103%	80.272%
88.661%	79.882%	90.494%	92.539%	77.962%	78.955%	91.554%	86.739%
86.700%	73.591%	66.538%	82.740%	84.499%	92.384%	91.427%	76.838%

88.479%	67.596%	69.802%	83.911%	87.909%	92.584%	91.156%	72.527%
89.136%	74.862%	65.999%	82.045%	86.151%	93.654%	92.150%	76.120%
89.341%	66.896%	72.691%	84.129%	89.778%	93.627%	92.529%	76.758%
91.406%	78.473%	71.119%	86.350%	86.192%	93.703%	92.298%	79.751%
91.935%	75.223%	81.188%	88.019%	90.950%	92.922%	92.859%	80.511%
90.434%	76.752%	72.930%	86.629%	85.994%	92.300%	92.349%	83.236%
92.407%	79.459%	77.960%	87.302%	90.244%	93.242%	93.136%	82.633%
92.453%	79.400%	78.455%	89.196%	87.198%	91.551%	92.769%	84.503%
93.381%	79.197%	82.287%	89.944%	87.421%	93.589%	93.362%	84.429%
90.636%	82.193%	82.680%	88.163%	85.563%	88.877%	91.712%	85.178%
92.447%	80.749%	81.056%	90.863%	82.418%	88.866%	91.261%	86.855%
94.484%	87.980%	86.612%	92.170%	76.382%	84.183%	92.342%	89.490%
92.498%	83.838%	84.149%	91.398%	84.327%	88.352%	90.158%	88.806%
93.156%	84.812%	82.506%	91.293%	76.439%	83.070%	89.721%	88.200%
92.003%	89.817%	88.406%	92.991%	73.866%	83.788%	89.995%	90.408%
90.562%	87.970%	87.643%	90.779%	76.803%	77.261%	86.818%	88.174%
90.441%	88.088%	90.172%	92.930%	71.506%	81.711%	85.774%	89.935%
89.790%	90.198%	89.310%	88.421%	72.100%	76.442%	81.564%	86.750%
89.046%	90.599%	92.871%	92.480%	75.108%	74.764%	83.880%	88.694%
91.272%	86.498%	88.956%	91.624%	72.482%	75.814%	81.623%	87.252%
89.364%	89.717%	92.514%	92.934%	73.906%	75.380%	81.368%	86.654%
88.118%	88.720%	89.380%	87.887%	72.306%	74.112%	77.295%	84.726%
89.784%	91.526%	92.392%	92.431%	67.623%	70.939%	80.460%	83.622%
90.758%	93.204%	92.449%	95.179%	73.677%	73.728%	78.375%	85.085%
81.119%	86.818%	92.769%	91.952%	55.089%	63.092%	70.997%	78.655%
87.499%	93.569%	94.444%	92.700%	65.457%	71.813%	75.733%	83.400%
78.708%	88.959%	94.644%	92.045%	54.212%	63.750%	69.917%	76.649%
78.604%	81.739%	85.033%	80.891%	51.505%	65.299%	75.295%	71.950%
72.112%	81.857%	92.278%	89.507%	53.769%	64.980%	73.535%	73.245%
68.853%	61.664%	47.880%	72.911%	78.271%	74.581%	88.158%	67.225%
90.493%	56.811%	63.867%	71.250%	83.067%	89.465%	89.702%	70.655%
76.809%	64.189%	54.217%	84.793%	82.715%	77.070%	90.799%	71.971%
89.480%	58.921%	73.590%	82.074%	85.711%	90.627%	90.124%	75.009%
81.410%	70.964%	61.743%	81.582%	84.063%	82.934%	91.817%	75.807%
89.947%	64.109%	71.894%	83.253%	86.866%	90.150%	90.575%	76.179%
87.617%	67.960%	62.038%	87.122%	87.648%	89.033%	92.513%	78.881%
90.647%	74.631%	75.745%	84.942%	88.829%	92.085%	92.673%	77.670%
86.284%	74.112%	71.594%	89.420%	86.599%	91.293%	93.159%	81.819%
93.382%	75.612%	79.695%	87.305%	88.933%	91.652%	91.875%	83.745%
86.815%	74.329%	74.852%	90.097%	88.120%	84.377%	91.949%	84.291%
92.719%	76.840%	84.316%	88.356%	88.616%	89.790%	91.176%	84.365%
93.813%	81.293%	81.489%	90.964%	87.713%	85.594%	92.984%	87.284%
92.163%	80.202%	82.777%	87.991%	87.620%	89.658%	90.778%	86.346%

91.880%	87.315%	86.064%	90.834%	85.736%	84.504%	89.172%	89.164%
91.507%	86.914%	88.424%	89.618%	84.849%	85.909%	88.112%	91.458%
91.213%	89.325%	88.016%	89.500%	84.296%	81.237%	86.402%	90.769%
90.388%	87.946%	88.535%	89.356%	80.953%	82.562%	85.247%	89.998%
89.222%	88.719%	87.745%	88.524%	81.297%	79.222%	84.075%	90.221%
86.762%	88.745%	90.876%	88.473%	77.357%	81.138%	83.374%	91.567%
85.204%	87.822%	87.814%	86.183%	75.392%	77.886%	79.144%	87.499%
83.761%	88.758%	89.631%	86.884%	74.873%	79.842%	81.934%	89.200%
82.550%	86.798%	88.176%	85.432%	65.003%	75.494%	77.384%	82.489%
84.987%	90.697%	90.354%	86.924%	71.180%	77.367%	78.235%	86.661%
86.211%	93.924%	92.852%	90.516%	65.643%	76.431%	79.948%	86.158%
81.859%	92.260%	93.139%	92.196%	65.027%	74.090%	75.832%	83.055%
83.173%	91.994%	92.762%	87.913%	53.403%	70.078%	77.618%	80.286%
79.069%	89.504%	92.754%	89.733%	56.741%	72.918%	74.532%	78.735%
92.260%	72.866%	77.491%	94.667%	91.408%	91.450%	91.433%	82.435%
92.076%	73.299%	89.465%	92.494%	92.668%	90.282%	87.042%	89.958%
95.298%	80.496%	89.280%	95.015%	91.351%	93.443%	91.829%	87.181%
95.107%	88.819%	93.348%	94.258%	93.014%	93.127%	90.819%	90.816%
94.386%	81.811%	94.487%	94.858%	90.507%	93.717%	92.721%	90.920%
95.851%	90.891%	94.227%	94.125%	92.217%	92.126%	90.721%	94.358%
95.723%	83.407%	95.557%	94.636%	90.942%	92.708%	91.691%	93.867%
95.389%	93.872%	95.464%	93.807%	91.418%	93.721%	92.974%	94.928%
94.312%	92.319%	95.794%	93.973%	91.302%	92.317%	90.570%	94.651%
95.126%	94.441%	95.724%	96.083%	92.080%	92.617%	90.912%	94.942%
94.929%	93.427%	96.029%	95.587%	91.541%	92.079%	90.115%	95.553%
96.080%	94.904%	95.399%	95.918%	91.842%	91.720%	91.404%	95.771%
94.470%	94.779%	95.640%	95.428%	90.932%	91.689%	89.588%	95.523%
94.621%	94.196%	96.053%	95.668%	87.997%	88.801%	88.570%	94.311%
93.079%	95.964%	96.218%	95.995%	88.149%	89.228%	86.312%	95.337%
94.549%	95.849%	95.768%	95.712%	85.163%	87.585%	86.615%	94.555%
85.233%	84.436%	84.702%	90.560%	92.029%	88.054%	90.638%	87.124%
89.291%	74.051%	84.019%	87.632%	88.712%	90.317%	88.586%	84.971%
84.525%	81.930%	87.021%	89.342%	82.653%	86.879%	89.880%	86.093%
88.221%	75.709%	86.266%	88.795%	86.988%	89.561%	90.414%	83.813%
88.625%	88.488%	88.823%	91.955%	86.998%	85.683%	91.347%	88.199%
88.443%	74.316%	86.167%	90.087%	84.092%	87.888%	88.211%	84.124%
88.673%	86.887%	89.218%	91.970%	83.101%	88.075%	89.916%	90.630%
87.083%	69.422%	88.906%	90.091%	81.979%	88.408%	89.870%	85.410%
89.705%	89.564%	89.948%	91.841%	81.536%	87.656%	90.436%	89.471%
86.382%	77.602%	84.770%	89.249%	77.491%	85.015%	85.644%	86.491%
91.300%	89.223%	92.154%	92.499%	82.694%	87.913%	91.471%	92.117%
84.587%	77.513%	85.547%	89.980%	74.294%	78.634%	83.843%	85.113%
90.757%	88.143%	91.524%	90.363%	79.884%	88.848%	89.702%	90.839%

86.280%	84.101%	88.367%	91.408%	80.558%	78.973%	86.752%	87.124%
93.084%	94.154%	94.441%	92.314%	79.229%	87.524%	89.499%	91.216%
90.245%	87.255%	92.074%	94.953%	74.796%	77.327%	87.853%	87.052%
92.813%	91.115%	94.212%	93.810%	77.615%	86.609%	87.993%	89.123%
86.242%	81.811%	91.535%	93.635%	69.776%	72.038%	85.149%	83.653%
91.138%	93.288%	92.443%	93.546%	75.249%	77.984%	86.525%	86.420%
86.338%	73.177%	92.410%	93.540%	56.356%	73.442%	87.024%	75.969%
84.723%	70.710%	80.037%	88.390%	83.466%	87.056%	82.480%	78.833%
82.809%	60.894%	66.899%	84.129%	80.839%	85.724%	79.237%	72.534%
86.861%	60.668%	60.652%	89.465%	80.753%	89.445%	84.712%	83.910%
89.114%	67.160%	85.330%	88.276%	81.475%	90.230%	80.779%	81.342%
89.958%	63.444%	60.991%	86.622%	80.970%	89.302%	87.202%	85.575%
85.365%	69.610%	85.086%	86.261%	76.708%	83.232%	78.079%	87.967%
89.754%	66.801%	77.953%	88.755%	80.216%	87.860%	86.677%	89.186%
88.077%	64.781%	87.716%	86.183%	76.973%	87.880%	81.654%	91.509%
91.448%	81.380%	86.290%	89.496%	71.816%	84.612%	86.479%	90.874%
86.313%	68.096%	89.613%	91.972%	76.973%	84.858%	81.501%	87.678%
89.630%	83.800%	88.534%	92.136%	74.555%	83.602%	88.160%	90.538%
89.755%	69.352%	91.173%	91.426%	68.993%	86.088%	82.821%	88.448%
89.094%	81.362%	89.020%	89.998%	70.522%	81.006%	87.014%	89.779%
87.983%	68.294%	83.723%	90.873%	65.037%	80.093%	75.017%	86.611%

odel C_Slice					Mod		
**Center 5%**	**Center 10%**	**Lower 2%**	**Lower 5%**	**Lower 10%**	**3slice_2%**	**3slice_5%**	**3slice_10%**
88.701%	84.674%	51.695%	75.205%	81.989%	73.232%	78.206%	86.960%
89.544%	89.337%	63.238%	67.278%	88.153%	49.656%	83.617%	91.691%
88.878%	86.456%	61.906%	75.758%	87.018%	72.411%	79.398%	86.835%
91.781%	88.064%	72.477%	81.798%	89.170%	55.454%	88.058%	91.990%
89.663%	89.706%	62.211%	76.578%	86.782%	70.148%	80.438%	89.053%
93.343%	90.561%	80.009%	85.417%	91.186%	72.503%	90.540%	93.397%
87.987%	88.380%	76.228%	76.896%	88.793%	82.281%	82.137%	87.906%
91.094%	89.491%	81.690%	86.538%	91.090%	62.706%	88.987%	90.917%
90.976%	89.693%	76.635%	77.411%	89.937%	80.642%	82.086%	86.633%
93.613%	87.848%	87.073%	86.012%	92.684%	85.308%	91.022%	92.057%
91.190%	90.228%	81.035%	80.902%	90.390%	83.652%	86.869%	91.549%
94.375%	92.154%	90.181%	89.373%	93.840%	71.595%	91.208%	93.916%
92.504%	91.072%	81.219%	85.119%	91.827%	84.884%	89.539%	91.008%
92.934%	89.435%	90.261%	89.981%	93.506%	65.565%	91.014%	92.451%
93.405%	91.600%	89.019%	89.866%	95.515%	74.845%	92.361%	93.832%
94.805%	90.669%	93.044%	92.486%	95.796%	67.782%	94.096%	95.422%
93.010%	91.699%	93.350%	90.655%	95.134%	85.581%	88.394%	92.029%
95.441%	92.873%	94.533%	93.646%	95.818%	66.000%	92.885%	95.582%
91.347%	91.562%	94.738%	92.423%	95.193%	86.174%	91.854%	92.842%
95.891%	93.037%	95.212%	94.314%	96.608%	62.377%	93.455%	95.088%
91.524%	92.086%	80.424%	92.291%	91.809%	86.890%	88.673%	90.795%
95.619%	94.348%	73.227%	91.426%	89.074%	73.587%	88.363%	91.628%
91.468%	90.843%	83.510%	92.047%	90.182%	83.053%	86.874%	90.488%
94.592%	94.522%	74.801%	90.340%	89.506%	74.625%	87.079%	88.052%
92.997%	92.078%	88.273%	92.804%	92.416%	86.803%	90.767%	90.357%
94.256%	91.607%	77.274%	92.549%	91.301%	73.190%	88.059%	91.395%
92.598%	92.583%	88.549%	93.333%	91.753%	86.979%	91.139%	93.113%
91.558%	88.621%	80.075%	94.672%	94.482%	71.981%	90.538%	92.811%
86.977%	89.366%	87.604%	92.115%	91.864%	76.402%	93.112%	90.765%
91.809%	87.751%	87.268%	93.393%	94.845%	70.700%	93.239%	94.111%
85.178%	86.313%	87.091%	91.213%	93.162%	65.677%	94.226%	93.019%
90.250%	87.141%	92.314%	90.454%	93.227%	72.961%	92.562%	91.618%
91.824%	82.439%	74.332%	85.169%	82.770%	68.467%	85.616%	88.736%
89.815%	83.940%	80.822%	84.986%	86.058%	80.602%	86.086%	90.998%
91.229%	85.378%	78.234%	87.203%	83.174%	84.041%	82.947%	90.282%
92.848%	86.989%	81.783%	87.102%	88.243%	83.516%	85.528%	91.114%
93.408%	87.933%	81.767%	87.425%	86.687%	84.112%	87.052%	90.702%
94.288%	88.795%	85.321%	91.038%	88.849%	86.875%	90.236%	92.950%
90.277%	86.331%	81.808%	88.361%	86.184%	83.881%	83.160%	89.184%
92.279%	84.078%	86.137%	92.267%	89.700%	88.351%	88.182%	91.513%

94.621%	86.899%	91.763%	91.983%	94.352%	91.222%	92.968%	93.731%
94.938%	85.569%	92.539%	94.278%	94.094%	91.611%	93.453%	93.561%
94.398%	88.656%	88.728%	91.654%	94.012%	91.473%	93.331%	94.201%
94.641%	87.521%	92.417%	94.374%	93.929%	90.439%	93.621%	94.037%
93.905%	94.034%	86.087%	89.002%	90.152%	85.602%	91.003%	93.184%
91.478%	89.354%	88.453%	89.298%	89.163%	84.864%	87.974%	88.147%
93.375%	93.723%	88.920%	88.637%	92.178%	85.945%	92.447%	92.553%
94.210%	92.722%	86.270%	92.465%	90.553%	83.662%	92.513%	93.173%
93.686%	93.667%	86.958%	89.377%	91.284%	86.143%	92.130%	93.101%
93.490%	92.447%	89.216%	91.576%	92.799%	85.168%	93.226%	93.909%
94.906%	94.081%	88.379%	90.797%	92.281%	88.170%	93.126%	94.493%
94.686%	93.965%	89.790%	93.847%	92.780%	84.872%	92.627%	93.578%
93.823%	95.053%	89.919%	91.589%	92.694%	86.031%	94.418%	94.329%
94.657%	93.373%	90.871%	91.727%	92.794%	84.853%	91.762%	93.549%
93.874%	94.562%	92.686%	93.484%	95.191%	89.296%	95.639%	95.136%
94.671%	94.510%	94.133%	94.070%	95.274%	85.260%	95.047%	95.381%
94.041%	93.664%	91.303%	92.413%	95.240%	86.887%	92.802%	94.035%
94.486%	94.154%	93.038%	95.605%	94.589%	81.253%	94.412%	95.426%
89.905%	92.001%	61.213%	88.749%	87.854%	86.795%	81.038%	89.676%
90.700%	90.986%	58.253%	85.332%	86.438%	85.624%	90.542%	85.737%
89.043%	92.396%	67.658%	89.421%	89.535%	88.624%	89.911%	90.110%
91.515%	92.491%	61.754%	85.821%	86.294%	88.465%	92.315%	90.941%
88.934%	91.177%	74.219%	90.084%	89.403%	86.236%	88.264%	87.439%
89.861%	91.547%	62.406%	88.686%	86.638%	87.566%	92.632%	89.234%
86.851%	89.015%	77.645%	90.356%	88.613%	84.627%	87.213%	87.347%
86.821%	88.924%	70.030%	89.641%	87.493%	87.132%	92.078%	87.333%
88.252%	90.405%	82.976%	90.943%	90.689%	85.117%	89.374%	87.872%
88.550%	90.994%	76.725%	91.312%	91.468%	90.237%	91.411%	87.408%
88.291%	90.633%	85.007%	92.517%	90.782%	87.561%	89.815%	89.551%
86.926%	89.687%	86.401%	93.146%	90.103%	90.840%	92.143%	88.179%
86.844%	92.650%	88.162%	94.661%	92.742%	86.122%	91.089%	91.494%
85.537%	90.744%	89.103%	94.324%	91.283%	92.246%	92.216%	89.026%
84.989%	90.878%	91.241%	94.896%	94.327%	88.731%	92.320%	94.006%
84.964%	92.048%	89.659%	95.045%	92.128%	92.431%	94.450%	90.729%
81.776%	90.915%	93.908%	94.555%	93.826%	86.270%	92.214%	92.753%
86.519%	93.423%	91.646%	94.683%	94.508%	90.024%	94.961%	90.736%
91.817%	91.697%	77.288%	86.556%	92.927%	84.448%	89.637%	92.718%
87.274%	87.294%	67.688%	85.901%	90.712%	67.471%	77.987%	91.643%
91.496%	92.911%	75.757%	87.700%	90.441%	86.136%	88.428%	92.634%
92.016%	84.831%	76.172%	87.048%	93.259%	66.947%	78.574%	88.481%
92.281%	92.530%	79.650%	88.751%	92.103%	83.708%	86.779%	92.058%
89.059%	83.089%	81.378%	86.515%	90.133%	63.664%	83.513%	87.966%
89.834%	92.052%	84.436%	89.904%	93.377%	82.239%	88.966%	91.754%

87.954%	83.901%	84.562%	85.673%	89.727%	69.130%	87.173%	87.231%
87.959%	90.324%	85.764%	90.807%	92.456%	79.162%	85.764%	91.128%
88.986%	84.102%	87.772%	88.871%	90.845%	58.942%	90.115%	89.928%
89.065%	87.746%	74.490%	82.105%	86.750%	89.991%	86.511%	90.872%
87.070%	82.311%	79.918%	84.499%	90.204%	28.193%	80.408%	79.900%
89.026%	87.178%	80.838%	80.239%	88.493%	83.750%	90.107%	91.760%
88.152%	81.527%	81.686%	85.201%	88.958%	33.000%	85.325%	84.762%
88.499%	87.592%	79.672%	82.956%	90.180%	84.891%	92.291%	91.688%
87.677%	83.868%	81.590%	89.218%	90.287%	31.298%	87.405%	85.654%
89.714%	87.594%	81.646%	82.957%	91.190%	81.515%	90.491%	86.954%
85.781%	82.091%	83.143%	87.040%	88.703%	41.709%	85.605%	85.048%
90.187%	83.738%	81.807%	86.212%	94.446%	80.338%	92.500%	89.829%
90.085%	86.401%	82.038%	90.260%	91.576%	45.591%	88.317%	88.613%
92.108%	92.272%	83.764%	86.821%	86.125%	84.442%	88.400%	91.757%
86.672%	92.719%	75.691%	81.118%	82.220%	85.058%	87.459%	90.870%
94.495%	94.614%	81.463%	87.653%	88.267%	86.679%	89.648%	93.139%
89.003%	92.745%	78.691%	83.428%	78.488%	81.964%	85.819%	91.064%
90.940%	92.302%	85.140%	86.337%	89.262%	87.647%	89.483%	91.298%
91.622%	92.176%	81.530%	88.092%	84.739%	85.398%	89.858%	90.042%
92.552%	94.100%	87.552%	90.677%	89.263%	87.931%	90.704%	90.685%
93.855%	94.286%	82.073%	87.835%	88.466%	86.142%	90.176%	91.418%
92.627%	93.433%	89.247%	90.662%	92.186%	90.649%	91.691%	92.414%
92.968%	92.158%	85.798%	92.296%	90.337%	89.762%	90.539%	90.660%
91.690%	93.529%	91.495%	91.674%	93.931%	88.507%	93.936%	90.143%
92.496%	88.969%	91.332%	93.728%	93.206%	86.089%	91.967%	93.472%
92.821%	91.460%	91.349%	91.687%	92.849%	86.754%	92.574%	90.288%
93.543%	90.325%	91.856%	94.399%	93.991%	77.271%	92.921%	91.853%
93.020%	93.641%	93.170%	94.914%	94.270%	90.572%	94.384%	91.862%
91.372%	87.122%	92.034%	93.927%	93.177%	85.239%	89.968%	91.689%
88.380%	90.277%	94.993%	93.948%	95.373%	86.560%	93.711%	90.840%
90.957%	88.425%	94.968%	94.681%	95.356%	85.594%	93.020%	94.928%
88.263%	91.821%	93.578%	94.130%	94.993%	91.506%	94.323%	93.475%
88.770%	85.707%	94.558%	93.475%	94.052%	85.821%	92.861%	93.549%
88.142%	89.895%	93.902%	95.764%	95.041%	91.960%	94.841%	95.292%
89.179%	82.488%	94.151%	92.120%	94.509%	87.203%	93.783%	95.078%
92.028%	92.156%	84.611%	85.550%	87.405%	85.203%	89.143%	92.206%
90.005%	93.138%	77.589%	85.691%	83.974%	86.338%	87.008%	92.267%
91.422%	92.593%	84.525%	85.415%	88.378%	86.643%	88.520%	92.960%
91.284%	93.739%	78.865%	86.967%	85.829%	83.778%	86.844%	92.202%
92.381%	90.705%	86.954%	88.804%	90.007%	86.064%	88.843%	91.502%
92.897%	92.267%	80.437%	88.568%	88.576%	83.564%	85.579%	91.281%
92.808%	92.000%	88.448%	90.660%	90.836%	86.646%	90.142%	92.527%
92.313%	91.523%	85.419%	89.704%	90.081%	84.549%	87.697%	90.716%

92.442%	92.592%	90.486%	93.329%	93.140%	90.005%	92.918%	94.126%
92.800%	92.474%	87.434%	93.073%	92.295%	89.727%	90.398%	92.535%
92.224%	92.742%	91.962%	94.259%	93.239%	91.719%	94.027%	93.527%
91.601%	91.256%	88.338%	91.213%	90.878%	88.610%	89.749%	91.642%
91.306%	93.818%	90.868%	95.475%	92.275%	90.900%	92.713%	94.019%
92.415%	91.867%	90.137%	94.081%	93.432%	90.532%	91.723%	93.401%
92.006%	93.094%	94.541%	95.453%	95.790%	92.325%	96.312%	95.699%
91.054%	91.870%	93.599%	94.878%	94.495%	92.991%	94.966%	94.552%
90.738%	92.236%	95.042%	94.400%	95.495%	93.724%	95.191%	94.891%
91.165%	91.375%	94.777%	94.929%	94.277%	93.463%	94.616%	94.425%
90.592%	93.215%	95.293%	95.300%	95.222%	93.673%	95.104%	95.212%
90.866%	88.471%	94.368%	95.536%	95.418%	93.446%	95.417%	95.037%
79.639%	88.063%	63.671%	69.872%	65.954%	74.877%	75.276%	83.952%
82.110%	94.250%	67.917%	78.918%	80.979%	86.248%	85.643%	92.165%
82.554%	88.508%	65.441%	73.858%	75.127%	77.795%	80.141%	84.817%
83.790%	93.855%	71.382%	78.670%	83.529%	85.068%	85.775%	91.282%
89.753%	90.922%	72.529%	81.675%	83.404%	80.891%	85.084%	89.936%
88.798%	94.084%	74.239%	85.896%	86.237%	83.521%	86.647%	92.837%
87.914%	91.005%	74.150%	84.931%	83.810%	78.444%	84.663%	89.201%
91.989%	93.636%	78.750%	86.770%	87.985%	79.871%	87.719%	92.452%
92.350%	91.530%	80.621%	87.324%	89.332%	81.501%	88.240%	92.730%
92.014%	93.050%	83.701%	88.201%	87.902%	82.536%	89.239%	91.201%
92.593%	93.589%	82.733%	88.808%	87.931%	82.339%	89.102%	92.175%
92.263%	92.994%	85.618%	89.598%	89.645%	84.096%	87.603%	91.043%
93.726%	92.639%	84.763%	90.770%	89.146%	86.366%	90.229%	91.134%
93.237%	93.363%	88.262%	91.326%	90.471%	87.933%	90.341%	91.253%
92.544%	92.663%	88.670%	90.885%	90.004%	87.017%	89.157%	90.193%
94.146%	92.088%	90.333%	93.754%	90.918%	85.530%	91.596%	92.859%
91.559%	90.920%	88.160%	90.170%	89.538%	87.141%	88.586%	88.990%
92.908%	92.234%	91.131%	92.617%	91.651%	88.615%	91.305%	91.519%
89.267%	91.840%	88.326%	88.470%	89.057%	87.009%	88.011%	89.168%
91.137%	89.640%	90.813%	92.209%	91.722%	88.895%	91.362%	91.791%
88.886%	90.709%	88.625%	89.905%	90.268%	88.073%	88.984%	89.433%
89.583%	90.226%	92.521%	91.628%	92.767%	88.006%	93.595%	92.705%
90.786%	93.388%	95.435%	92.936%	95.709%	93.626%	95.324%	95.008%
88.082%	85.070%	95.317%	90.863%	94.631%	78.821%	94.163%	90.945%
88.505%	91.228%	76.062%	80.128%	88.611%	84.274%	81.439%	87.260%
91.630%	90.092%	81.157%	87.581%	86.947%	86.028%	86.382%	92.882%
90.450%	90.643%	78.622%	84.215%	86.565%	76.378%	85.788%	89.904%
92.686%	88.407%	83.202%	90.147%	91.431%	84.469%	89.898%	89.190%
93.231%	90.705%	85.502%	86.536%	91.300%	73.346%	89.288%	90.482%
92.672%	89.505%	87.284%	89.876%	92.595%	83.971%	90.618%	90.487%
93.921%	90.964%	89.868%	88.983%	93.971%	76.710%	88.895%	92.921%

92.214%	89.143%	92.941%	92.785%	94.443%	82.904%	93.217%	91.625%
94.970%	89.881%	89.383%	90.682%	95.452%	71.965%	90.209%	92.679%
94.202%	91.026%	94.833%	94.945%	95.742%	84.747%	92.879%	92.055%
92.074%	91.190%	90.852%	91.227%	95.382%	72.969%	90.348%	94.697%
93.484%	89.845%	95.245%	95.260%	95.922%	85.358%	94.485%	91.693%
89.823%	92.609%	80.974%	83.329%	82.797%	79.536%	83.664%	90.398%
88.261%	93.585%	84.284%	90.261%	90.110%	77.724%	87.088%	93.520%
93.185%	93.554%	83.758%	84.815%	86.155%	84.279%	85.636%	92.569%
90.881%	91.567%	84.309%	90.552%	90.127%	86.300%	86.246%	90.300%
93.786%	93.445%	84.851%	86.697%	86.438%	82.629%	84.851%	91.330%
94.312%	93.233%	82.997%	90.676%	90.783%	88.156%	88.788%	93.058%
92.871%	93.173%	87.595%	87.403%	87.810%	81.838%	89.088%	91.686%
95.783%	95.097%	86.711%	91.739%	92.355%	87.125%	87.725%	94.323%
93.755%	94.131%	92.109%	87.379%	90.689%	81.705%	91.247%	93.168%
95.836%	95.465%	89.267%	93.021%	92.361%	89.407%	90.256%	94.076%
91.672%	92.483%	90.042%	89.744%	90.964%	87.673%	92.498%	91.498%
95.192%	94.926%	90.306%	94.306%	93.379%	90.443%	91.981%	94.347%
90.989%	94.351%	92.639%	89.537%	92.133%	85.745%	91.458%	93.027%
95.664%	95.232%	90.457%	94.169%	93.612%	91.197%	92.839%	94.445%
91.523%	94.371%	94.521%	91.218%	94.899%	87.169%	93.140%	93.828%
94.097%	94.294%	93.514%	94.432%	94.259%	91.558%	94.865%	94.749%
92.309%	93.580%	93.656%	89.495%	94.955%	87.719%	95.315%	94.072%
94.195%	95.530%	96.033%	95.990%	95.470%	93.259%	96.108%	95.689%
92.181%	92.828%	94.577%	90.231%	95.316%	87.526%	93.708%	94.984%
94.385%	93.174%	96.399%	95.236%	95.672%	91.275%	96.158%	95.293%
86.686%	94.014%	66.657%	81.116%	81.643%	77.894%	90.662%	93.745%
86.279%	92.716%	63.188%	66.414%	85.883%	75.054%	87.672%	92.608%
87.765%	91.749%	62.885%	82.665%	82.268%	76.148%	90.230%	90.882%
87.967%	91.970%	69.003%	74.372%	84.569%	65.702%	87.941%	92.151%
87.915%	89.529%	68.523%	85.119%	85.146%	71.924%	89.107%	88.711%
90.338%	94.604%	68.813%	62.557%	86.324%	64.565%	88.659%	93.470%
87.366%	90.814%	63.858%	85.214%	85.579%	75.935%	90.474%	89.069%
88.586%	91.680%	71.196%	86.679%	86.548%	69.650%	88.658%	93.400%
90.145%	91.313%	72.595%	87.911%	89.213%	74.580%	91.724%	92.482%
89.637%	91.438%	76.787%	88.034%	88.834%	62.255%	89.542%	92.693%
88.932%	92.536%	74.895%	89.013%	89.369%	78.109%	88.898%	89.763%
88.625%	89.308%	74.879%	87.906%	89.158%	62.613%	89.971%	91.599%
89.969%	93.388%	75.762%	89.043%	88.879%	70.022%	89.935%	89.621%
92.080%	89.872%	85.120%	92.586%	92.374%	56.690%	93.608%	94.566%
86.239%	91.336%	78.472%	78.048%	81.912%	82.433%	85.707%	87.005%
88.913%	91.422%	79.944%	84.091%	85.029%	86.800%	90.305%	88.697%
86.803%	90.777%	79.704%	77.253%	85.716%	84.139%	85.136%	87.426%
89.491%	89.469%	76.325%	83.688%	84.834%	86.570%	88.488%	88.314%

87.757%	91.025%	81.064%	76.922%	84.716%	83.255%	86.027%	86.561%
89.249%	86.412%	77.206%	84.681%	86.544%	79.220%	89.164%	87.928%
89.253%	89.075%	82.725%	81.196%	89.859%	84.684%	89.073%	89.116%
87.300%	81.862%	82.886%	82.484%	86.309%	74.828%	85.485%	82.469%
88.842%	89.873%	81.978%	81.469%	87.297%	86.452%	89.172%	89.479%
88.233%	81.116%	86.989%	85.998%	89.368%	76.998%	91.804%	89.994%
91.647%	89.825%	85.388%	80.425%	87.514%	87.257%	89.423%	91.150%
90.515%	84.687%	86.565%	88.028%	89.457%	81.279%	92.395%	89.917%
91.683%	91.844%	87.120%	86.130%	89.569%	88.109%	91.272%	92.652%
87.715%	83.008%	88.286%	88.860%	89.592%	78.677%	92.959%	89.661%
89.829%	89.223%	88.345%	86.491%	88.899%	87.076%	91.139%	92.086%
91.309%	83.193%	90.876%	91.578%	90.906%	80.915%	93.330%	91.586%
87.984%	89.820%	88.104%	86.434%	87.976%	88.283%	92.621%	92.179%
90.932%	87.586%	90.224%	92.652%	90.825%	82.741%	93.050%	93.976%
89.833%	89.033%	86.047%	89.837%	87.233%	89.013%	88.291%	87.204%
89.624%	86.394%	93.811%	94.544%	93.167%	88.104%	94.119%	92.724%
92.102%	88.713%	94.482%	93.584%	94.270%	92.564%	93.852%	93.295%
89.655%	87.515%	92.451%	94.405%	94.142%	85.850%	90.944%	88.567%
89.391%	88.660%	94.321%	89.734%	93.877%	90.293%	92.714%	92.193%
90.563%	85.654%	92.668%	94.719%	93.430%	87.863%	94.224%	93.985%
82.261%	69.561%	69.711%	70.701%	84.849%	64.299%	61.093%	60.594%
83.799%	83.199%	70.999%	82.413%	87.370%	69.238%	74.707%	71.659%
89.976%	77.413%	76.072%	74.743%	87.796%	70.934%	69.821%	73.538%
85.837%	83.402%	72.743%	80.351%	89.132%	76.211%	78.625%	76.812%
93.306%	89.193%	74.483%	85.667%	88.974%	83.042%	87.898%	85.278%
90.810%	87.728%	80.322%	86.054%	91.883%	83.257%	90.351%	83.634%
91.305%	87.871%	74.108%	85.286%	87.234%	80.899%	85.721%	85.695%
91.828%	90.379%	79.227%	89.894%	88.628%	80.267%	88.900%	88.911%
92.254%	91.446%	78.268%	88.092%	89.468%	81.858%	87.815%	88.899%
92.658%	90.044%	81.439%	91.008%	88.361%	83.533%	88.721%	88.061%
90.146%	88.785%	82.142%	86.328%	89.648%	79.363%	87.852%	84.742%
92.067%	89.849%	79.073%	91.137%	89.533%	85.398%	88.960%	89.349%
92.286%	90.583%	85.043%	88.897%	87.759%	81.995%	87.718%	91.524%
93.950%	93.183%	85.017%	94.054%	91.311%	88.364%	89.845%	93.522%
92.049%	88.903%	83.882%	90.818%	87.434%	82.652%	88.072%	88.623%
93.532%	92.503%	86.811%	92.418%	91.387%	86.508%	91.805%	90.998%
92.485%	91.011%	88.632%	90.835%	90.683%	86.021%	89.104%	90.537%
94.043%	94.205%	86.743%	93.984%	91.241%	87.983%	92.127%	93.904%
94.789%	90.869%	91.317%	94.990%	94.112%	91.084%	94.018%	94.267%
95.646%	94.237%	91.892%	95.951%	94.855%	91.411%	94.052%	95.326%
93.873%	88.206%	93.363%	94.628%	94.701%	90.599%	92.211%	93.145%
95.160%	93.980%	92.788%	96.022%	94.952%	92.039%	94.622%	94.466%
93.933%	88.353%	91.586%	93.590%	95.217%	91.111%	93.288%	93.713%

94.919%	93.025%	92.849%	95.347%	95.299%	92.698%	94.303%	94.602%
88.526%	87.314%	73.538%	83.774%	85.468%	80.004%	85.633%	88.205%
89.450%	89.523%	68.029%	57.137%	77.608%	41.351%	88.877%	86.987%
87.988%	86.270%	74.520%	80.799%	84.895%	71.481%	84.691%	86.318%
90.097%	93.137%	68.983%	76.417%	82.460%	38.471%	90.124%	90.154%
86.615%	85.430%	78.027%	80.355%	85.530%	72.339%	82.656%	85.042%
91.533%	94.459%	74.439%	87.315%	86.053%	47.669%	89.633%	90.598%
90.953%	91.426%	81.328%	85.930%	89.604%	73.407%	84.699%	90.177%
92.546%	90.357%	80.482%	86.041%	88.144%	63.267%	92.070%	88.292%
93.350%	90.598%	85.094%	86.134%	90.306%	81.682%	90.218%	91.259%
92.661%	93.161%	83.752%	88.999%	88.518%	54.391%	92.932%	92.428%
90.492%	90.151%	87.970%	83.737%	91.567%	72.205%	90.047%	90.130%
93.479%	91.565%	86.462%	89.558%	91.722%	65.455%	91.084%	91.577%
85.031%	85.831%	82.091%	79.944%	87.903%	82.112%	85.866%	87.147%
94.808%	91.335%	88.225%	89.859%	91.236%	66.704%	90.593%	91.824%
84.621%	81.408%	85.425%	78.502%	89.699%	73.512%	89.661%	88.859%
92.679%	86.164%	91.359%	86.498%	89.959%	65.835%	89.696%	90.687%
87.579%	83.581%	87.289%	81.371%	90.268%	73.303%	88.817%	89.791%
92.981%	89.358%	92.243%	91.692%	93.953%	65.613%	92.139%	93.293%
85.888%	86.889%	87.736%	87.041%	92.081%	78.389%	90.316%	91.157%
88.430%	84.460%	92.129%	88.693%	91.994%	76.278%	90.707%	91.795%
83.040%	93.075%	69.073%	78.775%	73.775%	78.957%	81.912%	83.720%
88.507%	93.672%	64.981%	79.636%	77.019%	84.696%	85.651%	90.478%
87.666%	94.070%	70.644%	81.229%	77.987%	81.077%	80.935%	87.327%
87.979%	94.399%	65.249%	81.773%	82.124%	89.196%	88.645%	91.488%
88.105%	94.273%	72.317%	83.016%	80.591%	82.759%	83.075%	87.070%
83.881%	92.559%	67.767%	80.022%	80.977%	87.308%	87.923%	88.147%
87.147%	94.876%	75.627%	84.588%	80.930%	84.949%	85.660%	89.103%
87.051%	94.200%	72.184%	84.766%	84.027%	80.555%	88.004%	89.681%
88.094%	94.931%	77.350%	84.865%	85.246%	74.258%	86.163%	90.349%
93.479%	93.772%	79.693%	89.502%	89.142%	75.291%	89.344%	90.473%
91.783%	92.269%	84.365%	92.342%	91.616%	86.219%	89.840%	93.666%
92.829%	94.573%	82.126%	92.418%	92.897%	88.199%	93.435%	90.672%
94.329%	92.848%	85.635%	94.365%	93.041%	85.333%	92.344%	93.872%
93.550%	93.902%	83.608%	91.563%	92.556%	68.556%	93.823%	92.346%
91.313%	89.139%	89.179%	90.544%	92.652%	84.046%	90.305%	91.503%
94.895%	91.818%	90.138%	96.097%	94.451%	60.629%	95.980%	92.561%
92.545%	93.297%	68.087%	86.421%	82.236%	83.962%	89.005%	88.194%
91.102%	90.258%	69.230%	85.552%	89.856%	86.827%	90.474%	85.141%
91.356%	92.042%	71.573%	84.925%	80.010%	85.493%	86.164%	91.027%
91.919%	92.728%	74.198%	87.801%	90.182%	88.567%	90.258%	92.913%
92.075%	93.210%	73.244%	80.771%	81.174%	85.098%	90.644%	92.227%
92.749%	92.857%	83.010%	88.072%	91.998%	89.544%	92.869%	92.357%

92.307%	93.855%	78.727%	85.603%	82.502%	87.136%	90.408%	93.215%
93.691%	93.467%	81.164%	90.587%	91.583%	90.849%	92.526%	91.426%
93.149%	92.477%	88.964%	90.721%	89.803%	86.829%	91.572%	91.954%
93.707%	93.430%	85.970%	92.385%	91.896%	90.240%	92.312%	91.854%
93.051%	93.373%	89.734%	94.125%	89.693%	88.324%	92.889%	93.416%
93.722%	93.498%	88.272%	92.854%	91.821%	89.806%	91.442%	92.448%
92.350%	93.575%	86.295%	92.601%	89.953%	89.075%	92.138%	92.236%
94.055%	93.376%	88.978%	92.367%	92.936%	89.774%	92.000%	92.967%
94.673%	93.020%	92.519%	94.586%	94.057%	88.798%	93.609%	93.498%
94.177%	93.611%	89.220%	95.231%	94.365%	91.102%	92.963%	94.121%
92.150%	90.648%	92.550%	95.333%	93.993%	88.588%	92.958%	93.326%
92.289%	89.731%	91.841%	94.154%	93.633%	89.376%	85.324%	93.640%
92.595%	89.838%	93.047%	94.375%	93.396%	88.373%	93.454%	95.228%
91.858%	92.643%	91.585%	94.860%	94.368%	90.369%	92.094%	93.842%
86.772%	87.690%	64.105%	86.478%	84.445%	75.151%	82.834%	84.855%
90.530%	85.713%	66.136%	80.707%	78.880%	45.790%	81.529%	86.416%
88.657%	87.047%	62.426%	87.470%	84.406%	83.209%	83.848%	85.226%
87.821%	85.314%	69.600%	86.678%	83.119%	55.815%	81.163%	84.623%
87.096%	87.647%	65.851%	89.125%	85.923%	71.523%	83.475%	83.491%
88.780%	85.928%	70.935%	88.338%	82.836%	66.514%	83.755%	86.978%
89.252%	88.017%	69.980%	88.997%	87.658%	78.232%	81.986%	83.693%
87.821%	86.081%	73.939%	86.973%	81.691%	68.134%	80.194%	85.056%
89.804%	89.402%	70.127%	88.594%	88.449%	78.316%	87.401%	86.668%
87.240%	85.849%	74.110%	86.331%	82.850%	70.813%	76.265%	88.058%
92.815%	92.533%	77.502%	93.320%	93.546%	83.204%	90.462%	87.056%
90.060%	88.143%	78.023%	87.596%	90.783%	75.054%	76.324%	90.835%
88.767%	89.812%	77.706%	91.895%	91.972%	84.521%	89.604%	88.625%
93.526%	92.818%	81.085%	92.137%	92.772%	81.150%	79.809%	92.348%
86.359%	88.784%	80.190%	90.900%	92.345%	75.799%	89.392%	87.892%
91.432%	90.565%	84.539%	89.815%	94.318%	78.440%	80.544%	86.352%
88.069%	89.502%	58.778%	77.304%	80.226%	60.845%	74.549%	86.003%
90.920%	89.400%	75.559%	80.248%	85.518%	70.941%	83.209%	88.775%
89.294%	91.203%	69.294%	80.466%	86.438%	62.111%	81.960%	89.090%
91.191%	92.167%	78.436%	84.085%	87.791%	71.084%	87.860%	89.875%
92.607%	93.340%	71.074%	84.638%	90.252%	63.196%	78.990%	90.505%
93.715%	93.132%	82.645%	86.679%	89.629%	76.491%	90.211%	88.541%
93.555%	93.896%	76.833%	84.773%	89.691%	59.825%	79.326%	92.592%
94.165%	93.314%	83.745%	84.644%	91.359%	52.791%	88.601%	89.881%
94.100%	92.761%	81.671%	86.085%	88.511%	64.122%	82.017%	91.706%
93.473%	91.522%	83.961%	86.530%	87.852%	61.746%	89.401%	88.030%
94.470%	92.953%	86.290%	84.114%	92.211%	66.745%	82.385%	90.804%
93.961%	92.838%	85.557%	87.745%	91.089%	60.856%	92.956%	91.528%
83.029%	85.052%	62.520%	78.284%	81.623%	88.123%	90.232%	92.125%

79.709%	89.424%	55.891%	68.640%	76.258%	87.953%	88.932%	90.297%
82.513%	86.603%	67.905%	77.024%	80.015%	87.128%	86.426%	90.070%
85.040%	91.266%	60.605%	73.885%	83.831%	89.092%	90.192%	92.490%
87.385%	89.527%	72.098%	81.490%	84.733%	90.040%	88.770%	92.348%
87.006%	93.238%	66.350%	78.393%	82.689%	88.245%	89.561%	91.202%
90.120%	89.948%	74.321%	82.408%	86.826%	90.150%	90.412%	92.223%
87.556%	92.550%	67.053%	79.043%	85.618%	88.753%	91.422%	94.361%
91.773%	91.914%	75.401%	85.268%	89.763%	90.598%	89.254%	91.893%
89.127%	93.401%	73.016%	81.494%	88.753%	82.864%	91.415%	92.484%
91.447%	90.528%	79.567%	85.918%	90.717%	89.057%	88.516%	90.265%
91.481%	93.367%	77.995%	82.689%	90.379%	76.836%	92.814%	90.565%
94.737%	95.101%	82.251%	90.476%	92.002%	90.660%	92.428%	93.748%
92.533%	93.692%	84.117%	86.933%	91.278%	77.836%	91.689%	90.899%
91.706%	92.386%	84.394%	86.608%	90.387%	84.106%	89.337%	89.094%
93.584%	94.060%	86.454%	90.942%	92.466%	83.239%	91.737%	89.212%
88.891%	91.531%	87.703%	89.545%	90.472%	86.812%	89.662%	89.610%
93.792%	92.324%	88.075%	92.062%	92.976%	80.921%	91.573%	89.689%
87.241%	90.024%	88.627%	90.549%	90.050%	86.945%	88.640%	90.614%
93.075%	92.243%	91.075%	92.392%	92.915%	79.648%	92.654%	93.071%
86.079%	92.749%	90.178%	90.308%	91.332%	86.816%	88.975%	91.940%
89.422%	90.036%	90.377%	91.267%	91.746%	80.455%	90.302%	89.656%
85.837%	86.973%	88.305%	88.865%	89.813%	85.520%	89.339%	89.038%
87.276%	90.629%	91.536%	89.555%	92.585%	82.952%	91.869%	90.043%
87.727%	91.924%	90.197%	90.388%	93.279%	87.784%	93.899%	94.000%
84.933%	84.821%	86.689%	84.977%	90.509%	71.416%	88.535%	88.008%
85.622%	91.259%	90.815%	89.741%	93.960%	87.756%	93.887%	93.515%
81.427%	82.004%	86.545%	82.056%	89.996%	68.792%	83.704%	89.768%
73.929%	80.374%	81.143%	78.303%	88.413%	79.564%	79.890%	86.353%
79.741%	76.111%	83.284%	79.933%	86.393%	63.196%	81.007%	87.043%
69.104%	76.650%	57.018%	63.419%	69.411%	77.050%	80.467%	89.495%
73.278%	82.732%	55.949%	61.464%	75.485%	86.403%	82.018%	90.813%
78.968%	83.941%	59.773%	73.411%	81.821%	83.879%	86.071%	90.044%
80.530%	85.115%	61.866%	72.195%	80.648%	84.485%	88.472%	92.494%
82.097%	84.480%	63.264%	74.249%	81.250%	85.758%	86.319%	90.844%
84.372%	85.267%	63.320%	76.336%	80.293%	86.360%	83.803%	91.082%
86.581%	88.221%	67.570%	79.063%	84.814%	84.399%	87.020%	92.175%
83.809%	86.160%	63.753%	77.830%	83.891%	86.822%	86.993%	92.506%
89.190%	90.609%	72.444%	83.750%	86.775%	86.365%	88.063%	92.461%
89.343%	91.120%	70.848%	82.454%	85.600%	87.647%	90.842%	92.731%
89.287%	90.912%	72.751%	85.002%	86.185%	88.679%	82.977%	92.266%
90.761%	91.827%	73.006%	83.558%	88.276%	88.310%	91.179%	93.237%
92.067%	93.648%	77.560%	87.767%	90.896%	89.448%	91.338%	94.047%
88.912%	90.164%	73.142%	84.441%	85.867%	89.830%	88.665%	92.833%

91.010%	92.988%	81.730%	90.423%	89.984%	89.540%	90.336%	93.068%
93.630%	92.943%	78.948%	90.747%	90.627%	87.720%	91.570%	91.287%
91.111%	93.031%	85.883%	92.014%	90.037%	90.298%	91.825%	92.120%
92.384%	91.563%	82.060%	89.492%	89.290%	84.978%	91.259%	90.631%
88.717%	92.130%	87.476%	92.016%	89.463%	89.400%	89.814%	91.281%
91.920%	89.761%	84.935%	91.918%	89.379%	85.976%	91.426%	88.510%
85.520%	90.109%	87.970%	89.640%	86.252%	88.332%	88.036%	88.032%
90.118%	90.265%	87.277%	89.838%	88.745%	82.382%	89.136%	88.690%
83.165%	88.314%	87.935%	87.488%	85.969%	86.629%	89.559%	86.493%
88.181%	87.629%	89.609%	89.975%	88.247%	82.652%	91.113%	88.399%
88.155%	88.709%	91.977%	92.427%	92.779%	90.391%	93.795%	93.917%
85.908%	83.941%	91.633%	90.237%	90.130%	84.748%	90.855%	86.986%
83.889%	86.998%	91.260%	89.807%	91.899%	89.270%	90.637%	90.881%
82.516%	80.546%	90.371%	88.115%	89.156%	81.505%	91.932%	86.352%
95.023%	89.519%	77.021%	88.042%	90.764%	86.423%	93.700%	94.965%
93.080%	90.911%	83.581%	90.040%	93.558%	88.461%	92.223%	92.456%
96.204%	93.084%	81.354%	91.654%	93.932%	90.609%	95.165%	95.520%
95.607%	94.358%	85.410%	93.089%	94.720%	93.409%	95.890%	95.811%
96.307%	95.184%	84.527%	93.917%	95.676%	90.534%	95.444%	95.961%
96.567%	94.900%	88.564%	95.087%	94.575%	93.191%	95.685%	96.463%
95.382%	95.036%	87.281%	93.750%	96.036%	93.601%	95.672%	94.875%
96.077%	95.746%	92.653%	94.878%	96.377%	93.787%	95.662%	95.839%
94.226%	93.560%	90.931%	92.766%	95.776%	94.158%	95.778%	95.139%
96.787%	95.873%	93.158%	95.502%	96.410%	95.357%	96.397%	96.623%
95.142%	94.741%	94.858%	94.100%	95.806%	94.128%	95.825%	96.173%
95.751%	96.100%	96.168%	94.499%	96.159%	95.174%	96.574%	95.887%
94.152%	94.307%	95.274%	93.653%	96.087%	92.879%	96.504%	95.747%
95.306%	94.576%	95.545%	93.863%	95.947%	95.492%	96.107%	96.264%
94.173%	93.053%	94.777%	93.722%	96.137%	95.591%	96.503%	96.151%
94.868%	93.760%	96.447%	94.205%	95.951%	94.507%	96.552%	95.860%
87.635%	90.525%	74.588%	81.866%	86.884%	77.572%	91.003%	85.229%
87.825%	85.792%	69.881%	85.743%	88.962%	57.682%	85.925%	87.213%
85.991%	90.358%	74.977%	82.427%	86.527%	75.690%	86.550%	85.758%
88.199%	87.437%	71.068%	85.591%	89.866%	62.384%	82.403%	89.410%
89.459%	91.227%	80.006%	86.661%	90.498%	78.852%	89.373%	87.683%
88.570%	87.930%	76.751%	84.041%	90.106%	62.986%	82.326%	88.007%
89.121%	91.507%	78.684%	89.458%	90.210%	84.837%	89.457%	88.295%
88.338%	88.393%	77.832%	87.455%	90.522%	61.268%	77.110%	88.261%
90.245%	93.238%	82.607%	89.327%	92.097%	85.869%	91.046%	89.699%
87.265%	86.706%	76.888%	86.508%	86.381%	60.451%	70.764%	88.364%
92.210%	93.632%	80.670%	91.061%	92.363%	83.976%	91.806%	92.422%
86.924%	85.561%	77.825%	87.805%	87.289%	48.862%	63.455%	85.744%
90.539%	92.869%	80.874%	89.941%	91.769%	83.558%	91.745%	91.265%

88.678%	88.483%	82.142%	90.168%	90.791%	56.498%	83.522%	89.940%
93.155%	93.595%	86.956%	93.913%	95.277%	79.947%	92.287%	92.827%
93.869%	91.797%	89.804%	93.463%	93.025%	47.960%	86.120%	94.153%
91.605%	92.978%	87.692%	93.653%	95.185%	77.000%	93.406%	94.310%
90.687%	87.904%	88.360%	89.811%	92.949%	40.412%	87.660%	90.831%
92.239%	89.775%	88.903%	92.772%	95.318%	77.903%	87.734%	93.109%
90.319%	87.814%	87.922%	91.425%	93.753%	41.223%	89.729%	88.893%
85.647%	89.331%	63.387%	77.196%	83.451%	66.645%	90.995%	91.859%
85.298%	84.220%	71.642%	78.101%	88.508%	23.987%	78.116%	83.567%
90.346%	91.013%	63.201%	79.166%	83.846%	55.925%	86.305%	87.890%
87.651%	88.501%	70.226%	82.532%	89.167%	28.157%	79.980%	88.863%
89.132%	91.572%	70.755%	81.663%	85.219%	66.124%	90.372%	92.142%
88.974%	87.205%	73.981%	85.829%	88.022%	26.440%	79.304%	85.687%
91.226%	89.297%	74.453%	82.436%	83.833%	70.346%	89.320%	91.784%
91.812%	87.073%	79.608%	87.338%	91.203%	29.330%	87.783%	89.483%
90.911%	89.113%	77.257%	83.470%	89.001%	60.467%	89.614%	91.125%
90.536%	86.434%	78.010%	88.941%	92.311%	32.505%	89.522%	91.038%
90.926%	90.486%	83.385%	87.209%	91.908%	58.098%	90.121%	93.650%
93.175%	87.760%	83.006%	90.909%	92.132%	32.958%	80.645%	93.134%
90.368%	88.001%	86.985%	86.913%	88.056%	57.589%	87.747%	91.179%
89.533%	87.495%	80.366%	88.659%	91.575%	39.620%	83.425%	86.888%

lel B_Subject			Model C_Subject			
ALLslice_2%	ALLslice_5%	ALLslice_10%	3slice_2%	3slice_5%	3slice_10%	ALLslice_2%
26.143%	71.607%	79.171%	72.912%	84.848%	83.492%	23.136%
31.379%	70.318%	59.770%	60.653%	90.541%	88.486%	29.449%
25.570%	72.424%	80.570%	62.054%	85.197%	84.919%	23.851%
40.909%	77.764%	73.798%	67.913%	90.612%	88.960%	29.900%
30.243%	74.604%	85.233%	66.168%	86.888%	87.850%	25.036%
55.757%	84.716%	79.482%	78.707%	92.000%	92.877%	48.596%
28.855%	77.558%	84.999%	77.349%	86.341%	85.958%	34.691%
54.286%	80.583%	79.299%	77.103%	90.056%	91.339%	49.858%
30.892%	81.579%	86.322%	71.002%	85.710%	88.849%	29.992%
57.437%	84.761%	86.799%	80.675%	88.694%	90.981%	57.295%
36.875%	85.103%	87.873%	85.495%	87.467%	88.900%	41.511%
52.750%	88.371%	81.988%	72.156%	91.703%	93.760%	47.807%
33.902%	86.490%	87.345%	81.318%	91.982%	93.010%	40.303%
46.457%	84.626%	83.448%	68.162%	90.521%	93.230%	36.764%
31.737%	85.609%	89.819%	76.328%	91.260%	93.330%	31.959%
48.808%	84.650%	86.759%	78.370%	92.470%	95.417%	49.036%
36.347%	87.142%	86.509%	82.964%	93.753%	94.354%	41.057%
52.886%	84.323%	80.535%	82.358%	93.652%	95.920%	56.004%
38.085%	85.846%	82.935%	84.006%	93.131%	94.350%	36.086%
47.936%	83.214%	82.228%	86.523%	92.776%	95.680%	52.297%
45.795%	89.019%	89.254%	86.274%	90.375%	90.226%	31.348%
50.949%	87.962%	91.303%	84.572%	89.042%	94.192%	63.859%
43.459%	87.262%	89.154%	84.814%	87.369%	88.678%	32.466%
42.782%	80.901%	85.908%	84.429%	87.595%	93.259%	51.561%
39.260%	87.069%	87.005%	80.924%	88.995%	89.981%	27.153%
42.617%	78.325%	85.824%	81.974%	88.255%	93.208%	45.559%
39.518%	89.149%	91.188%	80.498%	91.941%	91.850%	31.543%
34.287%	84.160%	78.348%	73.966%	84.824%	89.757%	39.625%
39.201%	87.141%	77.289%	73.582%	89.430%	93.043%	26.474%
39.994%	77.362%	76.915%	62.949%	89.491%	92.238%	38.238%
26.759%	87.102%	86.752%	73.287%	89.268%	91.911%	15.720%
30.905%	82.731%	75.974%	66.033%	89.875%	90.860%	31.220%
67.473%	85.148%	85.467%	79.901%	85.052%	85.559%	63.539%
74.460%	81.927%	85.106%	80.329%	84.794%	90.064%	75.847%
71.352%	84.994%	87.807%	83.305%	88.399%	88.362%	71.182%
77.411%	84.467%	86.044%	84.267%	84.183%	91.991%	77.006%
69.590%	85.868%	87.992%	82.921%	87.906%	90.224%	70.444%
81.367%	88.333%	89.258%	85.966%	88.241%	93.906%	81.057%
72.710%	83.326%	84.972%	82.759%	85.353%	89.188%	77.077%
76.246%	86.229%	87.739%	87.200%	86.787%	91.923%	80.616%

80.586%	89.541%	91.452%	90.754%	92.060%	93.335%	81.339%
77.717%	91.217%	93.043%	91.405%	92.583%	94.734%	86.344%
83.378%	90.842%	92.322%	88.481%	92.377%	93.922%	83.864%
75.986%	91.101%	92.857%	91.016%	93.144%	94.921%	84.960%
63.403%	86.818%	91.720%	86.971%	91.883%	93.080%	62.925%
59.687%	69.738%	87.949%	74.321%	91.559%	89.112%	64.334%
67.954%	85.910%	91.493%	84.602%	92.147%	90.087%	61.996%
63.866%	76.892%	89.078%	81.846%	93.060%	92.446%	68.716%
73.687%	86.330%	92.617%	85.568%	91.801%	91.662%	64.324%
62.406%	81.288%	92.685%	76.144%	93.979%	93.550%	66.900%
71.367%	87.828%	92.994%	86.888%	93.509%	93.224%	69.289%
61.524%	85.510%	91.831%	81.332%	93.654%	94.363%	64.880%
62.213%	88.620%	93.734%	85.886%	94.230%	94.149%	60.719%
59.935%	87.662%	90.608%	86.035%	94.161%	94.502%	64.424%
65.560%	89.890%	95.184%	90.131%	95.005%	95.132%	63.021%
59.200%	88.512%	93.478%	85.662%	94.903%	95.622%	68.159%
61.410%	88.221%	93.733%	87.005%	93.797%	95.129%	59.910%
58.963%	90.787%	92.900%	88.194%	93.448%	95.546%	63.197%
43.434%	84.069%	84.893%	87.812%	89.984%	90.526%	70.372%
45.086%	83.682%	88.586%	88.481%	88.013%	88.736%	78.051%
40.364%	84.750%	89.911%	88.159%	90.932%	90.503%	67.576%
45.020%	81.112%	92.238%	88.811%	89.628%	89.669%	76.326%
41.651%	83.126%	87.584%	87.635%	89.401%	88.509%	69.319%
45.623%	79.959%	88.571%	87.787%	88.684%	89.906%	74.836%
44.708%	81.383%	89.858%	87.226%	89.350%	87.899%	69.128%
46.907%	77.026%	90.220%	85.719%	88.686%	90.049%	70.100%
55.463%	81.276%	91.186%	86.573%	89.555%	88.052%	73.830%
51.119%	79.230%	92.328%	86.135%	89.814%	90.932%	74.981%
58.101%	82.305%	89.913%	87.993%	91.705%	88.955%	75.763%
58.697%	84.084%	93.493%	89.227%	90.022%	89.854%	78.575%
61.242%	83.911%	91.662%	88.525%	94.382%	90.883%	76.549%
56.843%	85.410%	93.044%	91.147%	91.176%	91.417%	80.351%
65.737%	85.831%	94.340%	87.119%	94.290%	93.664%	79.338%
64.983%	85.921%	93.625%	90.929%	93.812%	93.778%	80.773%
65.112%	86.374%	93.990%	88.584%	94.718%	93.228%	75.419%
69.835%	87.909%	94.023%	92.259%	94.809%	95.343%	78.273%
42.514%	84.357%	85.710%	76.598%	85.629%	90.201%	45.295%
34.969%	71.654%	53.878%	57.639%	57.685%	87.045%	32.121%
39.421%	85.920%	85.792%	78.008%	87.344%	90.755%	47.019%
28.359%	74.105%	55.110%	52.828%	74.866%	84.752%	27.614%
36.635%	86.248%	88.118%	77.571%	85.881%	89.268%	46.314%
26.290%	73.829%	53.389%	54.975%	77.027%	85.280%	29.734%
45.360%	88.445%	90.492%	81.066%	88.129%	91.010%	50.774%

31.952%	80.778%	80.046%	61.923%	85.636%	86.400%	39.264%
42.862%	86.374%	86.649%	75.927%	85.862%	88.502%	43.323%
36.275%	83.560%	74.586%	68.821%	89.031%	87.727%	50.296%
40.400%	85.398%	90.155%	85.901%	87.009%	87.089%	60.046%
24.153%	73.574%	78.119%	73.195%	90.572%	80.229%	50.104%
40.154%	84.274%	89.672%	82.622%	88.099%	86.500%	54.850%
30.850%	80.020%	84.947%	75.500%	86.661%	85.512%	64.491%
33.583%	85.907%	90.634%	79.507%	91.461%	88.336%	60.558%
21.927%	78.200%	55.724%	70.800%	89.590%	88.723%	50.982%
38.273%	83.545%	85.918%	83.772%	90.770%	87.423%	56.289%
31.671%	79.983%	80.962%	72.700%	86.539%	84.002%	52.611%
39.702%	84.353%	86.457%	85.111%	92.184%	85.270%	50.206%
36.086%	80.923%	78.949%	73.292%	89.270%	88.765%	60.826%
67.955%	87.340%	88.993%	85.165%	88.924%	93.763%	72.739%
80.180%	84.982%	86.229%	80.706%	89.523%	91.979%	78.778%
65.139%	87.884%	90.703%	86.608%	92.052%	93.988%	69.425%
76.621%	83.694%	86.588%	80.695%	88.007%	90.714%	78.559%
64.013%	87.564%	90.189%	88.173%	90.285%	90.914%	71.689%
80.909%	87.217%	90.033%	84.526%	89.689%	91.486%	81.135%
64.211%	87.142%	90.549%	87.419%	90.971%	91.962%	72.000%
75.996%	86.028%	89.974%	83.160%	91.116%	91.764%	76.967%
65.116%	90.011%	91.358%	89.063%	91.632%	91.433%	75.520%
78.513%	89.863%	91.433%	88.446%	91.502%	90.745%	80.489%
54.590%	88.090%	92.650%	89.272%	93.432%	90.754%	69.525%
76.180%	88.800%	93.565%	90.132%	92.227%	93.159%	75.033%
64.894%	83.383%	91.039%	88.970%	92.261%	90.130%	70.318%
71.154%	92.045%	91.989%	89.258%	93.499%	93.539%	76.293%
72.797%	92.089%	93.393%	91.279%	94.586%	92.739%	79.736%
76.869%	89.073%	92.393%	88.635%	93.244%	92.568%	83.168%
71.883%	91.301%	92.391%	89.861%	91.742%	91.504%	79.021%
83.259%	92.048%	93.893%	88.837%	94.596%	94.879%	88.896%
74.275%	91.849%	92.697%	91.881%	91.535%	92.670%	81.367%
77.871%	87.204%	90.714%	89.475%	93.070%	93.941%	65.654%
74.212%	91.941%	93.610%	91.992%	91.873%	95.397%	82.518%
67.480%	88.925%	94.140%	89.435%	94.347%	91.696%	64.181%
73.072%	87.082%	88.894%	84.709%	88.516%	89.287%	63.354%
77.076%	87.485%	84.652%	83.593%	87.394%	90.879%	77.407%
72.047%	87.062%	86.997%	85.133%	88.157%	90.642%	63.532%
72.847%	82.226%	84.462%	81.401%	86.287%	92.339%	75.400%
79.610%	87.310%	87.180%	85.498%	88.494%	88.254%	70.546%
74.391%	82.492%	83.940%	82.416%	86.511%	92.080%	76.036%
80.077%	84.599%	88.303%	86.888%	89.116%	90.128%	79.179%
81.288%	85.617%	86.875%	84.606%	89.383%	91.124%	81.348%

87.245%	89.929%	92.631%	89.790%	92.335%	91.993%	80.866%
83.975%	89.515%	87.081%	88.425%	90.914%	92.122%	83.380%
82.046%	88.604%	92.225%	92.002%	93.557%	92.053%	86.189%
82.543%	91.063%	87.281%	88.393%	90.917%	91.702%	86.153%
81.250%	91.355%	93.378%	93.273%	92.747%	94.605%	85.208%
87.053%	92.998%	91.878%	90.742%	92.722%	92.939%	88.301%
77.099%	94.022%	93.922%	94.183%	95.219%	96.050%	81.986%
89.393%	94.276%	94.866%	93.660%	95.146%	95.478%	89.313%
81.441%	90.356%	92.315%	93.757%	94.210%	95.577%	84.093%
84.209%	92.461%	94.708%	94.766%	95.255%	95.824%	90.239%
83.144%	91.591%	94.276%	94.199%	94.816%	96.087%	86.201%
85.369%	93.344%	95.190%	94.168%	94.992%	95.740%	88.666%
52.244%	72.687%	77.527%	69.730%	77.352%	88.355%	44.613%
63.515%	84.512%	81.090%	78.821%	81.700%	93.121%	53.363%
48.690%	76.506%	80.110%	74.653%	82.347%	86.761%	39.780%
66.569%	83.517%	79.964%	78.775%	79.438%	90.758%	64.145%
52.406%	78.089%	84.630%	77.873%	85.592%	91.221%	48.017%
67.525%	84.747%	85.268%	78.767%	86.214%	88.911%	59.993%
49.297%	79.082%	83.008%	78.128%	85.966%	89.347%	43.211%
64.830%	83.375%	86.073%	77.531%	87.692%	90.383%	64.115%
58.645%	83.941%	87.981%	83.080%	88.347%	90.448%	55.247%
71.623%	82.277%	87.925%	82.328%	88.834%	90.365%	72.355%
66.457%	78.465%	88.854%	84.230%	89.935%	90.843%	64.482%
69.987%	85.308%	88.928%	84.807%	89.041%	90.878%	74.246%
68.733%	84.695%	90.192%	86.642%	89.984%	91.213%	65.010%
77.510%	88.880%	90.364%	87.642%	90.571%	91.701%	81.298%
72.734%	84.976%	88.955%	86.590%	88.629%	91.612%	75.656%
82.890%	88.058%	90.965%	88.970%	91.626%	91.579%	84.402%
76.318%	86.350%	88.878%	87.804%	88.024%	90.275%	82.181%
73.979%	88.237%	90.171%	85.344%	91.232%	91.306%	85.657%
79.541%	88.365%	88.530%	88.140%	88.509%	90.891%	84.899%
82.330%	87.542%	89.294%	85.803%	90.976%	91.392%	85.772%
79.517%	88.572%	89.147%	89.521%	88.845%	90.557%	81.324%
77.804%	86.611%	92.731%	88.233%	92.821%	93.397%	85.220%
79.043%	93.853%	94.144%	93.585%	94.830%	94.877%	82.454%
71.912%	80.953%	93.340%	86.200%	94.533%	94.578%	81.662%
57.378%	81.718%	88.052%	83.201%	88.288%	90.752%	48.673%
67.641%	85.362%	89.771%	80.612%	89.172%	91.832%	65.889%
51.553%	81.375%	86.309%	79.932%	89.438%	91.231%	43.941%
55.961%	86.695%	89.468%	83.010%	90.486%	90.516%	68.502%
54.635%	84.942%	82.722%	71.817%	90.362%	92.862%	47.751%
59.100%	87.499%	87.658%	84.757%	89.582%	91.377%	70.234%
53.759%	83.973%	78.605%	74.746%	91.730%	93.604%	49.044%

62.164%	83.419%	87.870%	88.064%	91.295%	92.771%	71.546%
54.503%	83.190%	77.498%	88.467%	91.816%	93.913%	58.209%
62.653%	88.288%	89.939%	86.447%	94.382%	94.776%	64.227%
51.477%	87.497%	79.087%	76.087%	94.008%	95.246%	57.319%
64.614%	89.179%	92.208%	81.230%	94.784%	95.419%	69.474%
65.696%	76.928%	84.311%	83.684%	85.550%	91.741%	57.507%
77.358%	82.596%	90.218%	75.216%	89.546%	92.439%	58.619%
65.022%	80.472%	87.386%	84.915%	88.552%	92.631%	61.025%
75.344%	83.876%	89.879%	79.444%	90.408%	91.765%	65.796%
67.492%	78.603%	86.823%	84.101%	88.752%	92.084%	62.597%
77.110%	86.493%	89.271%	81.235%	91.792%	93.740%	77.559%
67.798%	82.317%	87.649%	84.924%	89.257%	91.975%	62.730%
84.161%	90.576%	91.061%	85.573%	93.210%	94.373%	86.165%
68.217%	82.608%	89.887%	86.451%	91.637%	91.577%	64.962%
87.824%	88.511%	92.142%	88.888%	93.467%	94.398%	89.346%
73.564%	86.653%	91.804%	87.083%	91.805%	92.168%	68.277%
89.104%	87.675%	92.802%	91.078%	94.183%	94.624%	89.711%
76.002%	86.516%	92.844%	87.030%	90.639%	93.121%	72.860%
89.010%	88.891%	93.335%	92.543%	94.604%	94.691%	92.319%
78.886%	89.273%	94.298%	87.305%	93.681%	94.228%	75.432%
89.606%	90.328%	94.868%	93.328%	95.173%	95.361%	93.761%
74.023%	87.267%	93.538%	86.837%	94.126%	95.040%	76.308%
89.094%	91.441%	95.840%	94.437%	95.890%	96.178%	94.034%
70.623%	88.362%	93.993%	85.837%	94.311%	95.003%	69.402%
88.379%	93.401%	95.587%	92.403%	95.537%	96.102%	92.447%
20.085%	86.044%	89.855%	81.148%	92.733%	92.060%	18.584%
17.508%	78.627%	67.638%	81.283%	88.466%	91.019%	29.462%
19.370%	83.841%	90.400%	72.421%	90.862%	91.899%	20.602%
18.659%	80.525%	82.417%	80.215%	87.498%	93.377%	23.955%
16.606%	85.156%	85.271%	80.888%	91.576%	90.753%	17.202%
15.731%	78.105%	77.099%	79.618%	88.112%	93.600%	23.607%
16.229%	87.282%	89.370%	67.190%	88.833%	91.870%	20.203%
12.293%	84.885%	80.455%	78.399%	89.780%	93.509%	25.116%
20.692%	90.933%	88.604%	81.261%	91.545%	94.685%	22.770%
11.002%	84.486%	78.523%	68.353%	85.128%	91.638%	21.414%
13.367%	89.758%	84.773%	78.967%	90.622%	93.283%	16.151%
12.905%	82.640%	73.613%	70.853%	87.335%	92.453%	20.058%
14.531%	86.855%	92.208%	66.027%	90.959%	92.730%	16.795%
11.861%	81.259%	79.081%	74.602%	91.397%	91.896%	13.957%
64.007%	75.065%	81.650%	83.922%	88.445%	90.065%	61.307%
75.611%	85.048%	86.055%	86.000%	89.624%	90.610%	75.743%
64.276%	79.913%	85.434%	83.845%	89.146%	85.237%	61.586%
67.167%	85.698%	84.276%	86.800%	88.557%	90.321%	68.676%

60.814%	78.524%	86.467%	82.209%	87.894%	86.575%	61.605%
60.043%	78.847%	88.280%	83.443%	89.292%	87.851%	63.355%
61.841%	80.761%	88.690%	84.238%	91.013%	85.398%	65.061%
62.574%	76.508%	86.678%	79.699%	86.319%	82.134%	53.936%
64.104%	83.351%	90.494%	86.413%	91.469%	89.289%	63.660%
65.383%	82.075%	88.774%	85.760%	90.157%	87.401%	57.784%
68.234%	83.437%	88.894%	85.149%	90.538%	90.150%	65.807%
73.007%	86.611%	91.472%	86.527%	93.007%	88.812%	59.598%
68.523%	88.096%	89.140%	87.470%	91.655%	91.121%	64.713%
69.675%	84.957%	89.476%	84.889%	92.598%	89.043%	52.299%
74.688%	86.673%	90.137%	87.620%	90.498%	90.322%	60.409%
70.699%	80.940%	92.853%	87.731%	93.887%	89.312%	60.410%
83.643%	86.797%	90.211%	87.123%	91.849%	88.819%	75.960%
74.307%	79.951%	92.282%	90.368%	92.992%	92.569%	66.937%
79.259%	86.503%	89.307%	90.675%	90.732%	90.930%	83.761%
77.206%	87.211%	93.074%	91.504%	94.432%	92.430%	81.929%
87.499%	92.889%	93.732%	90.914%	94.478%	94.558%	82.114%
74.846%	89.845%	91.512%	88.965%	91.926%	94.445%	78.183%
80.515%	89.018%	91.535%	89.519%	93.347%	94.244%	79.945%
81.740%	91.386%	91.075%	90.702%	92.477%	95.009%	84.147%
55.248%	67.091%	67.810%	72.165%	71.778%	64.217%	54.012%
59.178%	64.982%	73.117%	62.157%	75.366%	70.788%	55.713%
65.254%	76.318%	73.147%	81.011%	79.154%	72.050%	59.190%
64.036%	73.867%	76.197%	75.343%	78.140%	76.405%	60.422%
68.543%	85.664%	88.368%	85.853%	91.458%	83.738%	62.321%
71.600%	86.467%	84.600%	79.866%	85.303%	81.359%	68.694%
69.009%	82.633%	84.315%	84.088%	86.373%	87.117%	64.731%
75.277%	87.733%	86.842%	81.008%	86.735%	88.500%	74.172%
69.753%	85.930%	88.619%	86.203%	89.732%	91.477%	66.654%
67.752%	84.796%	88.598%	82.158%	88.438%	89.708%	68.946%
71.550%	86.601%	88.957%	82.731%	88.869%	87.404%	70.661%
68.858%	86.421%	89.485%	79.703%	88.265%	91.437%	69.829%
71.375%	85.532%	90.219%	84.978%	89.498%	91.263%	68.241%
70.974%	85.509%	92.326%	83.758%	90.747%	93.625%	72.326%
69.763%	82.208%	87.548%	87.156%	86.532%	88.839%	73.648%
74.587%	88.162%	91.571%	85.524%	91.194%	92.684%	78.905%
72.999%	86.888%	89.745%	88.774%	89.424%	91.999%	68.819%
67.124%	88.559%	91.579%	88.598%	90.661%	93.614%	74.938%
72.564%	91.483%	93.984%	93.743%	93.896%	93.746%	77.877%
80.453%	92.251%	94.231%	91.910%	94.481%	95.589%	83.702%
75.901%	88.113%	92.445%	91.062%	93.252%	94.253%	74.357%
78.667%	92.327%	94.108%	91.825%	94.455%	95.582%	84.905%
76.957%	90.568%	93.962%	90.684%	93.098%	95.035%	75.832%

75.124%	91.440%	93.817%	91.699%	94.571%	95.198%	85.661%
13.916%	77.411%	84.450%	81.430%	87.678%	87.180%	15.030%
12.204%	72.841%	61.289%	66.171%	91.096%	88.526%	12.602%
12.842%	75.957%	82.548%	75.018%	84.268%	84.573%	10.950%
15.194%	76.128%	59.990%	68.651%	91.387%	92.744%	14.116%
11.033%	74.219%	81.388%	75.864%	80.545%	81.531%	11.093%
23.287%	80.604%	79.566%	77.549%	91.963%	93.970%	23.131%
12.123%	77.928%	85.278%	81.844%	88.319%	88.471%	12.806%
35.274%	73.510%	77.256%	73.319%	91.954%	90.242%	25.432%
22.811%	79.036%	87.243%	83.750%	89.168%	88.224%	30.823%
14.715%	84.710%	78.335%	77.572%	90.935%	92.815%	20.839%
12.839%	77.581%	88.737%	77.016%	86.527%	89.245%	20.634%
23.931%	80.156%	89.064%	76.130%	91.578%	92.223%	26.832%
24.346%	77.233%	86.793%	78.944%	84.880%	88.012%	26.326%
22.422%	75.472%	91.391%	75.890%	91.122%	90.920%	26.978%
11.108%	76.166%	86.656%	73.798%	84.318%	85.497%	21.260%
18.201%	76.200%	90.509%	70.448%	89.395%	89.847%	26.589%
13.873%	76.641%	87.194%	74.293%	87.087%	90.632%	26.654%
30.324%	76.663%	92.536%	71.472%	93.373%	94.027%	29.489%
19.080%	78.443%	89.464%	81.526%	90.960%	91.660%	33.631%
31.954%	81.287%	90.002%	75.540%	92.291%	92.323%	33.204%
41.455%	87.291%	85.684%	78.839%	83.143%	91.067%	44.622%
54.946%	86.394%	86.159%	83.403%	82.169%	92.709%	60.575%
43.444%	89.494%	86.163%	82.138%	85.289%	89.693%	51.054%
67.224%	90.447%	88.306%	86.177%	81.218%	94.267%	67.166%
47.501%	86.649%	86.505%	84.229%	81.510%	89.789%	54.969%
46.801%	91.374%	81.592%	88.783%	75.516%	93.568%	54.935%
44.842%	89.426%	87.804%	90.199%	84.287%	91.455%	50.945%
55.250%	90.482%	83.905%	88.434%	81.122%	91.947%	58.708%
27.438%	84.676%	87.445%	84.464%	83.818%	93.476%	41.860%
34.114%	87.481%	89.150%	87.650%	88.669%	92.620%	43.224%
34.470%	86.400%	95.641%	91.853%	90.459%	94.140%	44.378%
38.270%	87.058%	87.681%	91.078%	89.704%	93.879%	54.897%
40.776%	88.333%	95.774%	92.978%	93.304%	95.308%	47.791%
24.962%	88.930%	89.789%	88.136%	86.926%	95.252%	43.765%
51.345%	87.315%	91.936%	89.840%	91.432%	92.903%	50.021%
35.726%	87.837%	92.067%	85.725%	91.090%	95.881%	41.429%
82.765%	85.171%	89.683%	82.192%	90.033%	90.962%	75.301%
87.549%	84.045%	79.208%	79.534%	89.418%	86.362%	78.797%
79.967%	87.089%	89.253%	86.849%	88.718%	91.334%	77.836%
83.371%	87.212%	84.094%	82.492%	92.208%	89.608%	76.009%
85.140%	89.442%	90.989%	86.114%	89.145%	92.253%	82.860%
84.767%	89.869%	87.979%	85.452%	91.674%	88.644%	78.009%

84.692%	88.599%	90.284%	86.315%	90.712%	92.967%	78.294%
85.398%	87.125%	90.157%	85.293%	91.188%	92.771%	74.804%
82.070%	89.240%	90.737%	88.023%	91.848%	92.071%	82.411%
86.782%	87.001%	93.162%	84.304%	91.007%	92.995%	72.728%
81.538%	88.927%	92.487%	88.482%	92.416%	93.736%	82.165%
86.046%	86.752%	91.191%	84.280%	91.103%	92.724%	76.602%
84.404%	90.413%	91.593%	88.182%	91.402%	91.647%	85.582%
83.968%	86.418%	91.058%	84.516%	91.492%	93.365%	75.304%
87.112%	90.040%	92.760%	88.315%	92.978%	94.565%	87.307%
86.594%	89.865%	91.707%	87.709%	93.038%	94.661%	78.004%
85.804%	88.843%	92.450%	86.602%	92.611%	94.126%	85.314%
86.614%	89.658%	92.808%	83.760%	93.270%	93.845%	75.704%
90.365%	90.512%	93.879%	87.917%	92.998%	94.474%	89.023%
88.278%	89.301%	92.761%	85.503%	93.605%	94.429%	79.189%
30.192%	81.153%	78.472%	78.692%	82.433%	84.459%	30.102%
19.597%	72.107%	65.830%	76.904%	86.001%	85.202%	14.425%
41.282%	84.880%	85.687%	82.339%	84.506%	85.127%	35.907%
26.103%	74.661%	74.199%	78.278%	82.511%	83.017%	30.709%
35.404%	82.911%	81.232%	84.052%	79.910%	83.995%	32.653%
39.560%	75.474%	83.214%	83.391%	85.877%	85.067%	44.023%
32.814%	82.257%	75.247%	82.012%	83.617%	83.897%	37.955%
29.201%	78.824%	83.874%	82.698%	82.374%	85.429%	46.573%
29.022%	83.099%	80.231%	82.714%	86.281%	86.290%	35.693%
32.497%	78.613%	79.927%	77.831%	77.702%	84.543%	42.583%
27.240%	83.657%	88.829%	82.967%	89.564%	90.070%	32.937%
28.165%	77.949%	89.817%	81.079%	72.699%	85.586%	38.752%
37.922%	85.552%	89.101%	84.420%	85.654%	88.265%	37.565%
34.215%	87.654%	85.059%	79.809%	83.364%	91.628%	39.168%
29.153%	85.749%	90.375%	77.505%	81.848%	89.267%	35.224%
34.361%	86.460%	87.009%	79.659%	86.841%	91.916%	43.990%
23.390%	76.489%	67.119%	47.224%	75.989%	87.318%	26.151%
51.944%	79.850%	84.452%	66.815%	87.610%	88.187%	40.993%
32.370%	79.836%	72.511%	54.651%	83.532%	89.280%	28.860%
51.359%	86.593%	87.593%	64.613%	88.464%	88.922%	44.911%
43.296%	79.410%	67.348%	57.784%	73.942%	92.232%	36.066%
46.766%	86.497%	88.202%	73.767%	89.226%	92.076%	42.245%
35.369%	83.493%	67.275%	58.047%	79.385%	91.900%	30.819%
56.305%	78.616%	82.027%	70.743%	90.231%	91.758%	43.782%
38.602%	84.276%	73.053%	66.285%	87.351%	93.857%	36.088%
54.434%	80.777%	68.360%	73.703%	89.136%	93.252%	53.593%
39.043%	83.454%	68.952%	68.201%	89.941%	93.621%	32.586%
41.884%	74.378%	65.188%	56.485%	87.011%	94.028%	43.211%
19.390%	90.517%	90.665%	89.124%	91.205%	91.176%	51.673%

39.834%	86.397%	89.699%	84.207%	88.237%	93.350%	56.227%
18.336%	92.511%	92.612%	87.720%	90.465%	93.256%	50.476%
47.212%	85.954%	90.878%	87.783%	91.087%	94.421%	64.072%
29.831%	93.554%	93.427%	88.213%	90.703%	94.160%	60.431%
34.456%	89.109%	90.992%	88.354%	91.343%	93.817%	60.821%
42.491%	92.930%	93.146%	89.235%	91.177%	93.817%	67.734%
36.792%	88.735%	92.770%	86.212%	89.850%	93.090%	60.400%
27.147%	90.526%	91.778%	86.174%	92.373%	92.800%	55.547%
13.569%	87.991%	92.094%	82.589%	92.216%	93.968%	51.527%
41.450%	88.001%	90.229%	84.309%	91.581%	90.788%	55.082%
33.167%	83.628%	89.972%	78.550%	92.586%	93.376%	51.654%
46.690%	89.282%	94.197%	84.956%	94.067%	93.328%	52.519%
15.423%	84.309%	88.339%	78.886%	92.970%	92.750%	41.279%
52.971%	87.767%	89.692%	85.051%	91.911%	90.648%	53.255%
29.139%	84.417%	89.594%	81.421%	94.116%	92.561%	45.814%
54.772%	85.242%	87.892%	82.841%	90.136%	88.826%	59.728%
19.761%	83.717%	88.789%	76.927%	94.128%	91.951%	38.924%
59.795%	84.869%	89.492%	81.019%	90.020%	87.439%	58.116%
29.316%	83.127%	89.951%	76.433%	94.132%	92.303%	40.455%
62.409%	87.792%	92.132%	80.498%	89.822%	90.458%	52.058%
30.179%	81.361%	88.238%	75.737%	91.893%	91.184%	39.688%
55.829%	83.973%	88.290%	79.839%	90.203%	89.294%	48.194%
35.003%	84.611%	91.532%	78.325%	91.743%	92.660%	43.194%
61.266%	86.704%	91.501%	78.571%	91.825%	93.782%	49.983%
25.122%	76.977%	85.650%	68.007%	89.597%	90.585%	30.944%
61.158%	83.578%	87.661%	77.677%	93.964%	92.501%	52.894%
44.633%	76.497%	83.819%	69.649%	89.004%	88.596%	42.380%
37.188%	75.432%	80.875%	70.031%	84.044%	81.538%	38.873%
23.044%	72.180%	70.768%	64.072%	85.233%	84.536%	34.826%
38.492%	82.994%	80.604%	84.078%	84.515%	91.081%	51.517%
50.353%	84.341%	78.623%	83.807%	89.337%	92.089%	48.672%
45.774%	88.844%	84.233%	86.682%	87.551%	92.585%	56.192%
49.859%	81.836%	81.824%	82.570%	89.613%	91.008%	57.721%
51.946%	89.612%	86.158%	86.535%	90.434%	92.484%	56.442%
51.294%	85.109%	87.134%	83.882%	91.264%	90.869%	60.574%
54.619%	89.968%	87.375%	89.835%	91.754%	92.445%	59.267%
53.606%	83.660%	89.264%	84.134%	90.759%	92.611%	64.220%
44.752%	89.743%	89.120%	91.056%	92.705%	92.380%	60.567%
55.186%	84.912%	89.165%	85.741%	92.133%	92.787%	64.331%
61.971%	89.399%	88.898%	90.282%	92.483%	90.606%	66.326%
49.392%	84.037%	91.020%	84.548%	92.611%	92.129%	67.296%
63.587%	91.182%	91.250%	87.715%	92.751%	92.024%	65.411%
45.633%	83.535%	87.499%	86.490%	89.016%	91.305%	67.193%

68.891%	87.673%	91.269%	86.286%	89.835%	90.054%	62.741%
55.756%	79.302%	89.177%	84.131%	91.550%	89.926%	66.867%
67.352%	86.250%	91.173%	84.276%	88.999%	88.765%	56.458%
63.724%	79.416%	86.575%	83.024%	89.622%	90.427%	62.159%
61.600%	87.010%	89.492%	84.556%	87.703%	87.296%	49.660%
60.782%	79.453%	86.426%	83.220%	90.094%	91.004%	54.654%
59.991%	82.899%	86.216%	80.052%	87.703%	86.946%	50.950%
60.270%	78.597%	84.471%	81.240%	89.314%	90.889%	57.574%
55.266%	82.137%	84.224%	77.933%	86.525%	85.676%	42.845%
67.675%	79.368%	83.954%	80.161%	88.816%	91.000%	57.271%
62.747%	89.819%	91.890%	83.994%	92.883%	92.724%	55.847%
68.722%	80.501%	85.440%	81.033%	92.149%	92.506%	53.527%
58.548%	84.423%	91.090%	76.910%	92.122%	92.089%	44.409%
63.955%	77.218%	79.517%	79.767%	92.749%	89.154%	52.620%
64.834%	87.690%	94.468%	90.034%	94.955%	94.809%	66.583%
61.732%	87.592%	93.354%	90.069%	95.053%	92.271%	73.893%
63.863%	85.919%	94.743%	91.574%	95.895%	96.013%	65.759%
65.247%	91.304%	94.581%	93.036%	95.019%	95.786%	79.296%
63.552%	85.296%	94.379%	91.686%	95.896%	95.796%	66.888%
65.085%	93.072%	95.728%	91.730%	94.165%	96.083%	73.039%
76.072%	85.622%	95.271%	93.372%	94.981%	95.209%	74.365%
67.583%	93.316%	95.971%	93.028%	95.427%	95.358%	68.502%
75.792%	88.451%	95.852%	94.048%	95.119%	94.889%	76.571%
67.526%	94.802%	96.037%	94.727%	95.771%	96.482%	75.006%
80.964%	89.999%	95.864%	95.334%	95.282%	96.202%	71.408%
66.048%	95.217%	96.014%	95.331%	95.995%	96.123%	70.309%
79.921%	88.518%	96.008%	95.294%	95.853%	96.177%	66.416%
68.077%	94.728%	96.251%	95.217%	94.759%	96.233%	71.073%
79.795%	90.490%	95.982%	94.905%	95.383%	96.341%	64.312%
64.304%	93.480%	95.766%	94.979%	95.296%	95.676%	68.427%
46.798%	85.513%	89.199%	86.779%	91.326%	88.112%	47.065%
49.647%	82.158%	77.949%	82.096%	84.894%	86.829%	37.682%
41.373%	84.165%	89.888%	85.295%	89.678%	85.686%	38.687%
48.689%	79.918%	85.351%	80.849%	83.660%	87.553%	39.118%
32.589%	85.777%	90.146%	80.729%	89.845%	88.284%	26.351%
45.816%	80.163%	87.729%	76.628%	83.821%	86.174%	31.600%
33.627%	86.882%	86.374%	87.249%	90.293%	90.755%	19.230%
28.566%	82.078%	87.543%	77.014%	85.705%	90.035%	23.855%
42.190%	88.475%	91.552%	86.188%	91.147%	91.273%	21.520%
35.746%	81.474%	82.583%	69.761%	82.513%	85.570%	30.175%
45.894%	89.112%	91.128%	80.331%	89.699%	91.881%	30.352%
33.295%	81.380%	84.974%	71.660%	82.778%	83.126%	26.725%
45.629%	83.879%	91.860%	78.878%	89.326%	91.207%	26.857%

41.316%	80.825%	83.518%	77.507%	85.398%	88.292%	35.099%
47.249%	86.411%	91.295%	84.948%	91.218%	93.556%	49.494%
31.164%	86.015%	59.703%	77.534%	88.620%	92.843%	37.402%
45.754%	83.487%	89.569%	76.261%	92.613%	95.286%	55.538%
32.160%	82.236%	40.863%	75.464%	87.039%	91.563%	42.074%
36.096%	80.215%	89.753%	78.101%	92.913%	95.033%	51.217%
34.071%	69.828%	44.619%	61.846%	84.022%	95.099%	41.124%
19.238%	79.289%	72.206%	53.865%	90.233%	86.291%	18.279%
10.763%	70.745%	36.150%	20.458%	58.166%	82.802%	9.713%
12.049%	77.476%	49.120%	32.185%	54.579%	87.569%	10.992%
10.270%	76.832%	50.044%	20.099%	80.391%	89.013%	9.168%
11.083%	83.876%	47.489%	28.567%	86.440%	90.499%	12.673%
12.002%	78.455%	38.523%	25.490%	89.209%	85.047%	10.842%
18.850%	83.108%	57.387%	38.806%	89.802%	89.759%	16.414%
12.948%	76.477%	41.074%	30.718%	90.430%	89.282%	13.232%
18.327%	81.742%	72.537%	59.019%	90.747%	89.998%	22.134%
23.694%	79.462%	54.822%	38.799%	86.893%	90.665%	15.318%
23.224%	82.349%	79.936%	52.457%	92.442%	90.767%	17.881%
21.713%	72.627%	43.743%	41.029%	86.695%	92.629%	21.139%
26.653%	80.777%	60.472%	43.006%	89.744%	90.427%	30.611%
19.061%	74.642%	45.756%	37.895%	84.381%	87.680%	16.171%

ALLslice_5%	ALLslice_10%
51.879%	83.051%
56.444%	90.646%
56.729%	84.598%
80.274%	89.699%
72.400%	85.141%
84.259%	92.958%
80.725%	86.490%
85.922%	91.213%
82.815%	87.321%
85.638%	91.017%
85.485%	87.951%
88.670%	93.617%
89.604%	93.149%
86.204%	92.321%
87.455%	94.077%
90.061%	92.364%
91.392%	94.598%
89.770%	93.534%
91.703%	94.166%
86.753%	92.409%
87.178%	91.865%
90.759%	93.079%
88.302%	90.639%
87.655%	88.883%
88.271%	90.461%
89.695%	90.097%
89.842%	91.536%
92.996%	86.771%
85.609%	91.549%
83.267%	87.689%
86.860%	90.296%
81.642%	87.070%
88.260%	88.167%
83.153%	86.637%
87.826%	87.162%
85.057%	88.961%
88.307%	88.843%
88.347%	90.865%
86.265%	86.435%
88.280%	88.671%

92.358%	93.389%
91.774%	93.412%
92.270%	93.992%
92.579%	93.714%
89.741%	93.359%
77.533%	91.882%
86.613%	93.433%
85.294%	93.074%
88.312%	92.942%
84.567%	93.351%
88.574%	93.929%
86.975%	94.369%
88.349%	94.403%
85.030%	93.316%
89.840%	95.821%
83.231%	94.295%
87.285%	95.113%
87.247%	93.599%
82.970%	88.426%
86.694%	90.575%
83.553%	89.996%
87.115%	91.679%
83.923%	90.981%
86.768%	87.877%
82.990%	91.388%
84.653%	88.441%
83.559%	90.673%
87.506%	91.460%
81.212%	91.557%
88.279%	92.832%
82.323%	94.624%
87.625%	92.848%
84.069%	94.047%
88.687%	94.830%
83.865%	94.214%
89.695%	93.299%
80.313%	89.155%
78.729%	72.172%
85.123%	89.919%
65.719%	78.208%
84.055%	89.834%
64.747%	78.846%
86.682%	89.600%

76.102%	81.462%
83.322%	87.434%
81.945%	85.696%
86.268%	89.692%
82.052%	78.834%
82.890%	87.101%
85.302%	78.752%
84.794%	86.441%
87.266%	83.521%
83.510%	86.686%
79.277%	81.804%
85.248%	90.058%
78.983%	83.035%
88.393%	90.885%
83.359%	88.300%
89.771%	91.847%
84.327%	88.771%
89.147%	90.734%
87.402%	90.493%
89.705%	92.057%
85.217%	90.341%
90.795%	93.359%
89.881%	92.111%
92.311%	93.449%
93.130%	93.867%
90.790%	93.961%
94.614%	94.247%
94.428%	95.371%
92.412%	93.877%
93.514%	94.003%
93.751%	95.246%
93.981%	93.083%
92.623%	94.427%
93.939%	92.952%
92.244%	94.806%
90.719%	89.257%
88.189%	87.256%
91.293%	88.898%
87.031%	85.173%
91.598%	89.815%
88.585%	86.585%
91.341%	90.035%
88.519%	90.166%

93.626%	92.541%
90.231%	90.804%
92.925%	92.940%
89.519%	89.805%
92.899%	92.936%
91.982%	92.856%
95.483%	95.350%
94.824%	95.281%
94.642%	94.415%
94.865%	95.142%
94.916%	95.524%
94.878%	95.825%
79.219%	78.545%
83.915%	81.977%
80.677%	80.475%
83.289%	83.712%
84.093%	85.458%
84.150%	87.452%
85.914%	82.995%
82.164%	88.175%
88.233%	89.513%
85.176%	89.474%
89.469%	90.313%
87.591%	89.979%
89.922%	91.585%
90.191%	90.445%
89.009%	89.117%
91.044%	91.772%
88.840%	88.868%
89.164%	90.926%
89.496%	88.940%
88.749%	90.617%
89.429%	89.380%
89.155%	93.089%
94.431%	94.096%
88.365%	93.850%
90.076%	91.756%
86.349%	91.487%
89.256%	90.093%
88.516%	92.227%
91.360%	92.671%
90.380%	89.767%
91.184%	92.912%

91.172%	90.883%
92.578%	91.172%
90.787%	92.606%
92.540%	95.321%
93.246%	94.425%
82.658%	84.806%
86.639%	87.899%
88.617%	88.481%
87.534%	87.906%
88.420%	87.934%
91.709%	89.467%
88.694%	89.390%
91.292%	90.110%
89.523%	92.607%
91.636%	91.430%
89.696%	92.337%
93.890%	92.976%
88.658%	93.128%
94.522%	94.469%
91.018%	95.295%
94.800%	94.262%
89.662%	94.806%
94.068%	96.017%
91.366%	94.845%
95.772%	95.653%
80.443%	89.274%
64.245%	92.928%
78.585%	86.183%
80.805%	92.291%
84.741%	85.726%
71.593%	92.696%
71.443%	84.631%
85.010%	90.697%
88.540%	91.584%
84.431%	90.339%
86.341%	90.821%
80.485%	92.741%
77.136%	91.179%
85.183%	94.031%
80.638%	87.703%
89.114%	87.950%
85.226%	87.152%
86.089%	85.748%

81.754%	86.167%
86.313%	88.577%
84.434%	92.206%
83.709%	85.572%
84.515%	90.334%
87.255%	87.506%
88.199%	89.596%
89.835%	91.938%
89.637%	91.826%
87.246%	90.949%
89.701%	91.871%
86.836%	93.315%
90.788%	91.879%
90.663%	93.589%
89.379%	89.972%
93.022%	93.266%
94.863%	94.177%
90.168%	91.890%
93.038%	94.389%
91.619%	94.277%
69.304%	68.709%
79.145%	74.373%
77.992%	82.988%
84.546%	85.376%
87.927%	92.211%
88.001%	88.182%
85.933%	88.217%
87.283%	88.428%
89.314%	90.250%
86.286%	89.223%
88.653%	89.075%
85.270%	88.776%
89.307%	89.961%
89.294%	88.913%
86.449%	88.247%
90.590%	91.959%
90.281%	91.836%
91.556%	92.576%
94.147%	94.756%
93.615%	95.259%
92.936%	94.161%
93.187%	95.196%
93.984%	94.655%

92.420%	94.382%
78.806%	88.131%
67.844%	87.510%
63.684%	88.196%
72.440%	90.258%
72.004%	84.627%
76.066%	89.148%
75.514%	91.944%
70.631%	87.788%
80.688%	90.478%
77.621%	90.613%
76.357%	91.108%
76.748%	91.169%
75.625%	86.516%
75.181%	88.778%
72.671%	87.215%
71.317%	88.527%
73.349%	90.471%
67.379%	91.151%
71.826%	91.241%
71.485%	88.758%
85.623%	85.279%
86.081%	87.942%
87.798%	86.905%
89.238%	87.895%
87.226%	86.800%
90.322%	87.302%
89.904%	88.232%
90.942%	90.446%
86.687%	89.064%
89.908%	93.143%
88.353%	94.087%
85.016%	92.802%
89.448%	94.932%
82.896%	91.261%
88.917%	91.961%
85.795%	94.629%
86.690%	91.432%
88.495%	91.191%
87.528%	90.443%
90.660%	93.112%
88.985%	90.590%
89.034%	92.762%

88.993%	92.972%
90.146%	92.585%
89.595%	91.605%
86.687%	92.188%
91.240%	92.691%
86.557%	92.621%
91.200%	91.872%
88.335%	92.825%
91.837%	94.139%
91.897%	93.655%
90.399%	92.322%
89.950%	93.735%
91.358%	94.053%
92.240%	93.124%
79.181%	83.787%
80.698%	85.540%
81.086%	83.812%
79.914%	84.597%
83.263%	85.982%
83.171%	86.344%
83.316%	86.311%
85.007%	86.803%
84.991%	84.801%
84.871%	83.246%
86.795%	90.311%
85.170%	84.156%
87.718%	88.612%
91.601%	89.144%
86.174%	88.211%
91.725%	88.468%
65.118%	80.947%
78.938%	89.176%
69.787%	79.034%
81.406%	90.905%
75.093%	80.134%
87.401%	90.829%
79.723%	71.830%
87.613%	90.551%
82.526%	82.197%
90.168%	89.846%
87.693%	85.009%
92.182%	89.536%
89.081%	91.641%

89.061%	91.168%
90.821%	92.407%
91.027%	93.559%
92.180%	93.041%
91.537%	92.585%
91.828%	91.983%
91.167%	93.196%
89.827%	89.899%
89.341%	91.770%
88.661%	90.783%
87.968%	90.067%
88.235%	90.810%
88.037%	89.342%
90.203%	89.942%
90.063%	89.471%
87.916%	88.953%
85.411%	86.885%
88.631%	88.294%
87.291%	90.064%
90.135%	91.796%
84.961%	88.040%
86.429%	86.324%
85.620%	87.153%
84.759%	87.777%
76.242%	82.388%
83.665%	89.669%
76.017%	83.384%
76.342%	79.425%
68.007%	81.378%
82.036%	76.993%
87.969%	82.559%
86.917%	83.333%
84.871%	83.510%
87.625%	85.786%
87.351%	85.515%
90.732%	86.192%
88.499%	86.165%
90.757%	89.272%
87.173%	87.159%
91.536%	92.099%
88.447%	89.810%
91.186%	91.875%
89.149%	88.181%

87.681%	88.089%
87.647%	85.685%
84.895%	86.050%
85.261%	84.483%
84.918%	84.995%
83.654%	83.638%
81.931%	83.132%
82.289%	84.091%
80.277%	84.205%
84.770%	83.844%
81.856%	89.382%
82.174%	85.167%
81.902%	87.546%
80.599%	84.901%
90.914%	91.785%
91.070%	92.564%
90.546%	94.465%
93.523%	94.821%
92.407%	94.511%
93.774%	95.636%
93.276%	95.840%
93.889%	96.123%
93.451%	96.338%
94.782%	96.622%
94.301%	96.183%
95.290%	96.141%
93.120%	96.425%
92.668%	96.289%
93.611%	96.066%
93.217%	95.251%
90.437%	91.696%
86.930%	85.607%
89.288%	89.619%
86.401%	84.030%
89.829%	91.906%
85.566%	83.864%
90.006%	91.424%
88.138%	86.719%
86.582%	91.941%
84.981%	84.057%
87.489%	91.157%
84.728%	81.169%
88.736%	90.878%

88.352%	86.265%
89.955%	93.253%
88.254%	90.143%
88.409%	94.109%
88.415%	88.354%
87.938%	93.444%
83.458%	87.035%
62.112%	86.668%
35.499%	83.585%
45.180%	88.772%
33.606%	87.867%
58.357%	89.369%
63.162%	83.908%
65.525%	89.472%
68.564%	83.340%
82.048%	90.939%
58.089%	82.267%
71.249%	91.863%
49.996%	86.197%
70.664%	90.384%
48.413%	77.244%
